# Supplementary material for: Characterizing dysbiosis of gut microbiome in PD: evidence for overabundance of opportunistic pathogens
Source: NPJ Parkinsons Dis. 2020 Jun 12;6:11. doi: 10.1038/s41531-020-0112-6 (PMC7293233; doi:10.1038/s41531-020-0112-6)
Supplement: Supplementary file 1 — Supplementary-materials [file 41531_2020_112_MOESM1_ESM.pdf]

Supplementary Material

Characterizing dysbiosis of gut microbiome in PD: Evidence for overabundance of opportunistic pathogens

Zachary Wallen et al.

Corresponding author Haydeh Payami (haydehpayami@uabmc.edu)

Contents

Supplementary Table 1. Subject Data
Supplementary Table 2. MWAS of dataset 1 conducted using ANCOM
Supplementary Table 3. MWAS of dataset 2 conducted using ANCOM
Supplementary Table 4. MWAS of dataset 1 conducted using Kruskal-Wallis
Supplementary Table 5. MWAS of dataset 2 conducted using Kruskal-Wallis
Supplementary Table 6. PubMed search results for *Porphyromonas, Prevotella*, or *Corynebacterium_1*
Supplementary Figure 1. Correlation Network Analysis

Supplementary Table 1. Subject Data

			Dataset 1					Dataset 2				
			PD		Control			PD		Control		
			N with data	Summary statistics	N with data	Summary statistics	P	N with data	Summary statistics	N with data	Summary statistics	P
		Number of subjects enrolled with complete data	212	-	136	-	-	323	-	184	-	-
	Microbiome	Number of subjects whose 16S sequences passed QC	201	-	132	-	-	323	-	184	-	
		Number of unique ASVs detected	201	4,863	132	3,315	-	323	9,188	184	6,667	
		Number of genera detected	201	404	132	333	-	323	527	184	441	
	Metadata	Number of subjects who passed sequence and metadata QC	199	-	132	-	-	323	-	184	-	
1	Age & Sex	Age	199	68.3±9.2	132	70.2±8.6	0.04	323	67.7±9.0	184	66.4±8.3	0.05
2		Sex (N & % male)	199	133 (67%)	132	52 (39%)	1E-06	323	206 (64%)	184	55 (30%)	2E-13
3	Geography	Seattle, WA	199	93	132	58	-	323	0	184	0	-
		Albany, NY		75		62	-		0		0	-
		Atlanta, GA		31		12	-		0		0	-
		Birmingham, AL		0		0	-		323		184	-
4		Stool sample travel time in days	190	3.3±1.9	129	2.6±1.5	2E-03	314	5.2±3.3	183	5.0±2.6	0.73
5	Race	Race (N & %White)	199	196 (98%)	132	132 (100%)	0.28	321	317 (99%)	184	183 (>99%)	0.66
6	Weight	BMI	192	26.6±5.5	128	28.3±5.7	0.02	312	27.4±5.0	180	27.9±5.9	0.62
7		Lost >10 pounds in past year	195	45 (23%)	126	15 (12%)	0.01	316	79 (25%)	181	21 (12%)	3E-04
8		Gained >10 pounds in past year	196	26 (13%)	129	10 (8%)	0.15	309	45 (15%)	179	20 (11%)	0.33
9	Diet	Fruits or vegetables daily	194	151 (78%)	131	116 (89%)	0.02	-	-	-	-	-
10		Meat, fish, poultry daily	193	110 (57%)	131	82 (63%)	0.36	-	-	-	-	-
11		Nuts daily	194	43 (22%)	130	36 (28%)	0.29	-	-	-	-	-
12		Yogurt at least a few times a week	191	68 (36%)	128	57 (45%)	0.13	-	-	-	-	-
13		Grains daily	192	132 (69%)	129	86 (67%)	0.72	-	-	-	-	-
14		Alcohol	194	116 (60%)	131	93 (71%)	0.05	320	133 (42%)	181	101 (56%)	3E-03

15		Tobacco	196	14 (7%)	131	5 (4%)	0.24	321	13 (4%)	183	13 (7%)	0.15
16		Caffeine	193	137 (71%)	131	100 (76%)	0.31	319	273 (86%)	183	161 (88%)	0.50
17	GI Health	Constipation (no bowel movement) in ≥3 days prior to stool collection	196	29 (15%)	129	2 (2%)	3E-05	302	54 (18%)	176	8 (5%)	2E-05
18		Diarrhea on the day of stool collection	196	6 (3%)	129	2 (2%)	0.49	301	12 (4%)	178	5 (3%)	0.61
19		GI pain on the day of stool collection	164	14 (9%)	120	8 (7%)	0.66	303	27 (9%)	179	3 (2%)	1E-03
20		Excess gas on the day of stool collection	196	27 (14%)	130	2 (2%)	9E-05	303	47 (16%)	180	8 (4%)	2E-04
21		Bloating on the day of stool collection	197	20 (10%)	131	3 (2%)	7E-03	305	36 (12%)	179	9 (5%)	0.01
22		GI discomfort on the day of stool collection (yes to any item 17-21)	183	104 (57%)	119	26 (22%)	2E-09	305	103 (34%)	176	26 (15%)	4E-06
23		Constipation (<3 bowel movements per week) in the past 3 months	191	82 (43%)	130	6 (5%)	6E-16	312	138 (44%)	180	31 (17%)	6E-10
24		Diarrhea in the past 3 months	189	32 (17%)	127	28 (22%)	0.31	306	80 (26%)	181	54 (30%)	0.40
25		Colitis	192	9 (5%)	130	2 (2%)	0.21	316	54 (17%)	180	24 (13%)	0.31
26		IBS	191	14 (7%)	130	8 (6%)	0.82	312	17 (5%)	178	14 (8%)	0.34
27		Crohn's disease	193	4 (2%)	131	1 (1%)	0.65	314	3 (1%)	180	0 (0%)	0.56
28		IBD	193	5 (3%)	130	2 (2%)	0.71	307	9 (3%)	178	4 (2%)	0.78
29		Ulcers	192	18 (9%)	130	9 (7%)	0.54	314	6 (2%)	180	4 (2%)	1.00
30		SIBO	-	-	-	-	-	305	0 (0%)	177	0 (0%)	1.00
31		Celiac	-	-	-	-	-	314	0 (0%)	177	0 (0%)	1.00
32		GI cancer	-	-	-	-	-	315	1 (<1%)	179	1 (<1%)	1.00
33		Intestinal disease (yes to any item 25-32)	193	38 (20%)	131	19 (15%)	0.24	298	80 (27%)	173	41 (24%)	0.51
34	Medications	Currently taking digestive medication	192	60 (31%)	126	22 (17%)	6E-03	-	-	-	-	-
35		Currently taking antibiotics	193	8 (4%)	130	3 (2%)	0.54	315	13 (4%)	179	7 (4%)	1.00
36		Taken antibiotics in past 3 months	190	24 (13%)	130	22 (17%)	0.33	308	63 (20%)	170	33 (19%)	0.81
37		Currently taking anti-inflammatory drugs	190	77 (41%)	128	56 (44%)	0.64	-	-	-	-	-
38		Currently taking probiotics	184	42 (23%)	128	33 (26%)	0.59	-	-	-	-	-
39	Parkinson Disease	Disease duration in years	199	13.8±6.7	-	-	-	323	9.2±7.1	-	-	-
40		Patients on carbidopa/levodopa	187	170 (91%)	-	-	-	313	266 (85%)	-	-	-
41		Levodopa dose, mg/day	181	764±574	-	-	-	313	563±443	-	-	-
42		Patients on dopamine agonist	187	99 (53%)	-	-	-	303	153 (50%)	-	-	-

43	Duration & Medications	Patients on MAO-B inhibitor	187	71 (38%)	-	-	-	318	86 (27%)	-	-	-
44		Patients on amantadine	187	49 (26%)	-	-	-	315	60 (19%)	-	-	-
45		Patients on COMT inhibitor	187	37 (20%)	-	-	-	320	13 (4%)	-	-	-
46		Patients on anticholinergics	187	8 (4%)	-	-	-	322	10 (3%)	-	-	-
47		Patients not on PD medication	187	3 (2%)	-	-	-	316	17 (5%)	-	-	-

Column “N with data” shows the number of individuals for whom data on the specified variable was available; for all metadata, only subjects who passed both sequence and metadata quality control (QC) were considered. “Summary statistics” for metadata are shown as mean±SD for quantitative traits, and number and percentage of individuals with positive response (yes) for dichotomous traits. 15 samples (all in dataset 1) yielded no or too few 16S sequences to be analyzed and were removed. Two subjects had unreliable self-reported metadata; they were included in analyses that required only sequences and case-control status but were excluded from all analyses that required any metadata (these subjects are identified as 10122.FP0016201 and 10122.GMWA.1090 in the dataset on NCBI SRA). P-values are two-sided testing the difference in the distribution of each variable in PD vs. control. Variables that differed in PD vs. control at a conservatively uncorrected two-sided $P<0.05$ were carried forward and included with case-control status in PERMANOVA and tested for their effects on inter-individual differences in microbiome composition (β diversity). Constipation (no bowel movement) in ≥3 days prior to stool collection, GI pain on day of stool collection, Excess gas on day of stool collection, and Bloating on day of stool collection were captured by GI discomfort on day of stool collection, hence only GI discomfort on day of stool collection was carried forward to PERMANOVA. Currently taking digestive medication (mainly laxatives or antacid) was not carried to PERMANOVA because it was no longer significant when adjusted for GI discomfort on day of stool collection.

Supplementary Table 2. MWAS of dataset 1 conducted using ANCOM

Sample size for ANCOM included subset of samples that had complete data on all covariates tested: N= 171 cases and 117 controls in dataset 1.
W= ANCOM score indicating the number of times a genus achieved FDR<0.05 as compared to other genera (maximum W possible: 444 in dataset 1, 560 in dataset 2).
0.8= Threshold at which results were considered significant (TRUE).

W	0.8	Kingdom	Phylum	Class	Order	Family	Genus
441	TRUE	Bacteria	Firmicutes	Clostridia	Clostridiales	Lachnospiraceae	Agathobacter
426	TRUE	Bacteria	Firmicutes	Clostridia	Clostridiales	Lachnospiraceae	Lachnospira
418	TRUE	Bacteria	Firmicutes	Clostridia	Clostridiales	Lachnospiraceae	Lachnospiraceae_ND3007_group
411	TRUE	Bacteria	Firmicutes	Clostridia	Clostridiales	Ruminococcaceae	Faecalibacterium
410	TRUE	Bacteria	Actinobacteria	Actinobacteria	Bifidobacteriales	Bifidobacteriaceae	Bifidobacterium
410	TRUE	Bacteria	Firmicutes	Clostridia	Clostridiales	Lachnospiraceae	Blautia
407	TRUE	Bacteria	Firmicutes	Bacilli	Lactobacillales	Lactobacillaceae	Lactobacillus
406	TRUE	Bacteria	Bacteroidetes	Bacteroidia	Bacteroidales	Porphyromonadaceae	Porphyromonas
400	TRUE	Bacteria	Bacteroidetes	Bacteroidia	Bacteroidales	Prevotellaceae	Prevotella
393	TRUE	Bacteria	Firmicutes	Clostridia	Clostridiales	Lachnospiraceae	Hungatella
391	TRUE	Bacteria	Firmicutes	Clostridia	Clostridiales	Lachnospiraceae	Roseburia
388	TRUE	Bacteria	Firmicutes	Clostridia	Clostridiales	Lachnospiraceae	Fusicatenibacter
384	TRUE	Bacteria	Firmicutes	Clostridia	Clostridiales	Lachnospiraceae	Lachnospiraceae_UCG-004
382	TRUE	Bacteria	Firmicutes	Clostridia	Clostridiales	Ruminococcaceae	Butyricicoccus
378	TRUE	Bacteria	Firmicutes	Clostridia	Clostridiales	Family_XI	Ezakiella
376	TRUE	Bacteria	Synergistetes	Synergistia	Synergistales	Synergistaceae	Cloacibacillus
374	TRUE	Bacteria	Firmicutes	Negativicutes	Selenomonadales	Veillonellaceae	Megasphaera
372	TRUE	Bacteria	Firmicutes	Clostridia	Clostridiales	Lachnospiraceae	Coprococcus_3
368	TRUE	Bacteria	Firmicutes	Erysipelotrichia	Erysipelotrichales	Erysipelotrichaceae	Coprobacillus
367	TRUE	Bacteria	Firmicutes	Clostridia	Clostridiales	Ruminococcaceae	Oscillospira
365	TRUE	Bacteria	Verrucomicrobia	Verrucomicrobiae	Verrucomicrobiales	Akkermansiaceae	Akkermansia
360	TRUE	Bacteria	Actinobacteria	Actinobacteria	Corynebacteriales	Corynebacteriaceae	Corynebacterium_1
356	TRUE	Bacteria	Proteobacteria	Gammaproteobacteria	Pasteurellales	Pasteurellaceae	Haemophilus
347	FALSE	Bacteria	Firmicutes	Clostridia	Clostridiales	Lachnospiraceae	Anaerostipes
331	FALSE	Bacteria	Firmicutes	Erysipelotrichia	Erysipelotrichales	Erysipelotrichaceae	NA
327	FALSE	Archaea	Euryarchaeota	Methanobacteria	Methanobacteriales	Methanobacteriaceae	Methanobrevibacter
326	FALSE	Bacteria	Firmicutes	Clostridia	Clostridiales	Ruminococcaceae	UBA1819
323	FALSE	Bacteria	Firmicutes	Clostridia	Clostridiales	Ruminococcaceae	Ruminococcaceae_UCG-013
319	FALSE	Bacteria	Firmicutes	Clostridia	Clostridiales	Family_XI	Anaerococcus
318	FALSE	Bacteria	Firmicutes	Clostridia	Clostridiales	Ruminococcaceae	Ruminococcaceae_UCG-004
306	FALSE	Bacteria	Firmicutes	Clostridia	Clostridiales	Ruminococcaceae	Anaerotruncus

302	FALSE	Bacteria	Actinobacteria	Actinobacteria	Actinomycetales	Actinomycetaceae	Varibaculum
293	FALSE	Bacteria	Firmicutes	Clostridia	Clostridiales	Ruminococcaceae	NA
275	FALSE	Bacteria	Actinobacteria	Actinobacteria	Actinomycetales	Actinomycetaceae	Mobiluncus
263	FALSE	Bacteria	Firmicutes	Clostridia	Clostridiales	NA	NA
252	FALSE	Bacteria	Firmicutes	Clostridia	Clostridiales	Lachnospiraceae	Lachnospiraceae_NK4B4_group
249	FALSE	Bacteria	Firmicutes	Clostridia	Clostridiales	Family_XI	Peptoniphilus
65	FALSE	Bacteria	Actinobacteria	Actinobacteria	Bifidobacteriales	Bifidobacteriaceae	NA
31	FALSE	Bacteria	Tenericutes	Mollicutes	Anaeroplasmatales	Anaeroplasmataceae	Anaeroplasma
20	FALSE	NA	NA	NA	NA	NA	NA
19	FALSE	Bacteria	Firmicutes	Clostridia	Clostridiales	Ruminococcaceae	Fournierella
17	FALSE	Bacteria	Proteobacteria	Gammaproteobacteria	Betaproteobacteriales	Burkholderiaceae	Alcaligenes
16	FALSE	Bacteria	Bacteroidetes	Bacteroidia	Bacteroidales	Prevotellaceae	Prevotellaceae_UCG-001
15	FALSE	Bacteria	Bacteroidetes	Bacteroidia	Bacteroidales	Prevotellaceae	Prevotella_6
15	FALSE	Bacteria	Bacteroidetes	Bacteroidia	Bacteroidales	NA	NA
15	FALSE	Bacteria	Firmicutes	Bacilli	Lactobacillales	Aerococcaceae	NA
14	FALSE	Bacteria	Firmicutes	Clostridia	Clostridiales	Lachnospiraceae	NA
14	FALSE	Bacteria	Proteobacteria	Deltaproteobacteria	Desulfovibrionales	Desulfovibrionaceae	Bilophila
14	FALSE	Bacteria	Firmicutes	Clostridia	Clostridiales	Peptostreptococcaceae	Peptoclostridium
14	FALSE	Bacteria	Actinobacteria	Coriobacteriia	Coriobacteriales	Eggerthellaceae	CHKCI002
14	FALSE	Bacteria	Proteobacteria	Gammaproteobacteria	Betaproteobacteriales	Burkholderiaceae	Paenalcaligenes
14	FALSE	Bacteria	Bacteroidetes	Bacteroidia	Sphingobacteriales	env.OPS_17	NA
14	FALSE	Bacteria	Proteobacteria	Gammaproteobacteria	Enterobacteriales	Enterobacteriaceae	Pseudocitrobacter
14	FALSE	Bacteria	Firmicutes	Bacilli	Lactobacillales	Leuconostocaceae	Leuconostoc
14	FALSE	Bacteria	Actinobacteria	Actinobacteria	Propionibacteriales	Nocardioidaceae	Nocardioides
14	FALSE	Bacteria	Firmicutes	Clostridia	Clostridiales	Ruminococcaceae	Ruminiclostridium
14	FALSE	Bacteria	Proteobacteria	Gammaproteobacteria	Betaproteobacteriales	Burkholderiaceae	Pelomonas
14	FALSE	Bacteria	Proteobacteria	Gammaproteobacteria	Pseudomonadales	Moraxellaceae	Enhydrobacter
14	FALSE	Bacteria	Firmicutes	Erysipelotrichia	Erysipelotrichales	Erysipelotrichaceae	Erysipelothrix
14	FALSE	Bacteria	Dependentiae	Babeliae	Babeliales	Vermiphilaceae	NA
14	FALSE	Bacteria	Actinobacteria	Actinobacteria	Micrococcales	Intrasporangiaceae	Ornithinimicrobium
14	FALSE	Bacteria	Firmicutes	Bacilli	Lactobacillales	Enterococcaceae	Melissococcus
14	FALSE	Bacteria	Proteobacteria	Gammaproteobacteria	Betaproteobacteriales	Methylophilaceae	Methylobacillus
14	FALSE	Bacteria	Actinobacteria	Actinobacteria	Corynebacteriales	Nocardiaceae	Rhodococcus
14	FALSE	Bacteria	Firmicutes	Clostridia	Clostridiales	Eubacteriaceae	Anaerofustis
14	FALSE	Bacteria	Bacteroidetes	Bacteroidia	Bacteroidales	Tannerellaceae	NA
13	FALSE	Bacteria	Bacteroidetes	Bacteroidia	Bacteroidales	Rikenellaceae	Alistipes
13	FALSE	Bacteria	Firmicutes	Clostridia	Clostridiales	Lachnospiraceae	Sellimonas
13	FALSE	Bacteria	Bacteroidetes	Bacteroidia	Flavobacteriales	Crocinitomicaceae	NA
13	FALSE	Bacteria	Proteobacteria	Gammaproteobacteria	Enterobacteriales	Enterobacteriaceae	Yersinia
13	FALSE	Bacteria	Actinobacteria	Actinobacteria	Streptosporangiales	Nocardiopsaceae	Nocardiopsis
13	FALSE	Bacteria	Proteobacteria	Gammaproteobacteria	Enterobacteriales	Enterobacteriaceae	Hafnia-Obesumbacterium

13	FALSE	Bacteria	Actinobacteria	Actinobacteria	Micrococcales	Micrococcaceae	Glutamicibacter
13	FALSE	Bacteria	Firmicutes	Clostridia	Clostridiales	Family_XI	Parvimonas
13	FALSE	Bacteria	Proteobacteria	Gammaproteobacteria	Betaproteobacteriales	Burkholderiaceae	Xylophilus
13	FALSE	Bacteria	Actinobacteria	Actinobacteria	Micrococcales	Microbacteriaceae	Pseudoclavibacter
13	FALSE	Bacteria	Bacteroidetes	Bacteroidia	Bacteroidales	Prevotellaceae	Prevotellaceae_Ga6A1_group
13	FALSE	Bacteria	Proteobacteria	Alphaproteobacteria	Rhizobiales	Xanthobacteraceae	Bradyrhizobium
13	FALSE	Bacteria	Actinobacteria	Actinobacteria	Micrococcales	Microbacteriaceae	Leucobacter
13	FALSE	Bacteria	Actinobacteria	Actinobacteria	Pseudonocardiales	Pseudonocardiaceae	Pseudonocardia
13	FALSE	Bacteria	Actinobacteria	Actinobacteria	NA	NA	NA
13	FALSE	Bacteria	Actinobacteria	Actinobacteria	Micrococcales	Micrococcaceae	Paenarthrobacter
13	FALSE	Bacteria	Bacteroidetes	Bacteroidia	Bacteroidales	Marinifilaceae	NA
13	FALSE	Bacteria	Actinobacteria	Actinobacteria	Frankiales	Geodermatophilaceae	Blastococcus
13	FALSE	Bacteria	Firmicutes	Bacilli	Bacillales	Family_X	Thermicanus
13	FALSE	Bacteria	Bacteroidetes	Bacteroidia	Flavobacteriales	Weeksellaceae	Empedobacter
13	FALSE	Eukaryota	NA	NA	NA	NA	NA
13	FALSE	Bacteria	Firmicutes	Bacilli	Lactobacillales	Enterococcaceae	NA
13	FALSE	Bacteria	Proteobacteria	Deltaproteobacteria	NA	NA	NA
13	FALSE	Bacteria	Entotheonellaeota	Entotheonellia	Entotheonellales	Entotheonellaceae	NA
13	FALSE	Bacteria	Proteobacteria	Gammaproteobacteria	Pseudomonadales	Moraxellaceae	NA
13	FALSE	Bacteria	Cyanobacteria	Melainabacteria	Obscuribacterales	NA	NA
12	FALSE	Bacteria	Firmicutes	Clostridia	NA	NA	NA
12	FALSE	Bacteria	Epsilonbacteraeota	Campylobacteria	Campylobacterales	Campylobacteraceae	Campylobacter
12	FALSE	Bacteria	Proteobacteria	Gammaproteobacteria	Betaproteobacteriales	Burkholderiaceae	Comamonas
12	FALSE	Bacteria	Firmicutes	Bacilli	Bacillales	Paenibacillaceae	Paenibacillus
12	FALSE	Bacteria	Proteobacteria	Alphaproteobacteria	Paracaedibacterales	Paracaedibacteraceae	Candidatus_Odyssella
12	FALSE	Bacteria	Actinobacteria	Actinobacteria	Corynebacteriales	Corynebacteriaceae	Lawsonella
12	FALSE	Bacteria	Bacteroidetes	Bacteroidia	Sphingobacteriales	Sphingobacteriaceae	NA
12	FALSE	Bacteria	Firmicutes	Bacilli	Bacillales	Bacillaceae	NA
12	FALSE	Bacteria	Firmicutes	Erysipelotrichia	Erysipelotrichales	Erysipelotrichaceae	Catenisphaera
12	FALSE	Bacteria	Proteobacteria	Gammaproteobacteria	Betaproteobacteriales	Rhodocyclaceae	NA
12	FALSE	Bacteria	Proteobacteria	Gammaproteobacteria	Betaproteobacteriales	Methylophilaceae	Methylophilus
12	FALSE	Bacteria	Proteobacteria	Gammaproteobacteria	Betaproteobacteriales	Burkholderiaceae	Herminiimonas
12	FALSE	Bacteria	Firmicutes	Clostridia	Clostridiales	Ruminococcaceae	Phocea
12	FALSE	Bacteria	Firmicutes	Clostridia	Clostridiales	Family_XIII	Family_XIII_UCG-001
12	FALSE	Bacteria	Bacteroidetes	Bacteroidia	Sphingobacteriales	Sphingobacteriaceae	Pedobacter
12	FALSE	Bacteria	Firmicutes	Clostridia	Clostridiales	Lachnospiraceae	Moryella
12	FALSE	Bacteria	Proteobacteria	Alphaproteobacteria	Sphingomonadales	Sphingomonadaceae	Altererythrobacter
12	FALSE	Bacteria	Firmicutes	Negativicutes	Selenomonadales	Veillonellaceae	Mitsuokella
12	FALSE	Bacteria	Fusobacteria	Fusobacteriia	Fusobacteriales	Fusobacteriaceae	Cetobacterium
12	FALSE	Bacteria	Proteobacteria	Deltaproteobacteria	Bdellovibrionales	Bdellovibrionaceae	Bdellovibrio
12	FALSE	Bacteria	Firmicutes	Clostridia	Clostridiales	Lachnospiraceae	Lachnospiraceae_FCS020_group

12	FALSE	Bacteria	Proteobacteria	Alphaproteobacteria	Rhizobiales	Rhizobiaceae	Allorhizobium-Neorhizobium-Pararhizobium-Rhizobium
12	FALSE	Bacteria	Proteobacteria	Deltaproteobacteria	Bdellovibrionales	Bacteriovoracaceae	Peredibacter
12	FALSE	Bacteria	Firmicutes	Clostridia	Clostridiales	Ruminococcaceae	Ruminococcaceae_UCG-008
12	FALSE	Bacteria	Bacteroidetes	Bacteroidia	Bacteroidales	Prevotellaceae	Prevotellaceae_UCG-003
12	FALSE	Bacteria	Actinobacteria	Actinobacteria	Actinomycetales	Actinomycetaceae	Actinotignum
12	FALSE	Bacteria	Proteobacteria	Alphaproteobacteria	Rhizobiales	Rhizobiaceae	Ochrobactrum
12	FALSE	Bacteria	Firmicutes	Bacilli	Lactobacillales	Aerococcaceae	Facklamia
12	FALSE	Bacteria	Proteobacteria	Gammaproteobacteria	Salinisphaerales	Solimonadaceae	Nevskia
12	FALSE	Bacteria	Proteobacteria	Gammaproteobacteria	Betaproteobacteriales	Burkholderiaceae	Simplicispira
12	FALSE	Bacteria	Firmicutes	Clostridia	Clostridiales	Peptostreptococcaceae	Peptostreptococcus
12	FALSE	Bacteria	Actinobacteria	Coriobacteriia	Coriobacteriales	Eggerthellaceae	Enterorhabdus
12	FALSE	Bacteria	Bacteroidetes	Bacteroidia	Bacteroidales	Rikenellaceae	Rikenella
12	FALSE	Bacteria	Proteobacteria	Alphaproteobacteria	Caulobacterales	Caulobacteraceae	NA
12	FALSE	Bacteria	Tenericutes	Mollicutes	NA	NA	NA
12	FALSE	Bacteria	Lentisphaerae	Oligosphaeria	Oligosphaerales	Oligosphaeraceae	Z20
12	FALSE	Bacteria	Firmicutes	Clostridia	Clostridiales	Lachnospiraceae	Herbinix
12	FALSE	Bacteria	Bacteroidetes	Bacteroidia	Flavobacteriales	Weeksellaceae	Elizabethkingia
12	FALSE	Bacteria	Firmicutes	Clostridia	Clostridiales	Family_XI	Helcococcus
12	FALSE	Bacteria	Actinobacteria	Actinobacteria	Actinomycetales	Actinomycetaceae	NA
12	FALSE	Bacteria	Proteobacteria	Gammaproteobacteria	Betaproteobacteriales	Rhodocyclaceae	Methyloversatilis
12	FALSE	Bacteria	Firmicutes	Clostridia	Clostridiales	Heliobacteriaceae	Hydrogenispora
12	FALSE	Bacteria	Cyanobacteria	Oxyphotobacteria	Chloroplast	NA	NA
12	FALSE	Bacteria	Proteobacteria	Gammaproteobacteria	Betaproteobacteriales	Burkholderiaceae	Verticia
12	FALSE	Bacteria	Spirochaetes	Brachyspirae	Brachyspirales	Brachyspiraceae	Brachyspira
12	FALSE	Bacteria	Firmicutes	Negativicutes	Selenomonadales	Veillonellaceae	Anaerovibrio
12	FALSE	Bacteria	Proteobacteria	Gammaproteobacteria	Betaproteobacteriales	Burkholderiaceae	Bordetella
12	FALSE	Bacteria	Firmicutes	Clostridia	Clostridiales	Lachnospiraceae	Robinsoniella
12	FALSE	Bacteria	Actinobacteria	Actinobacteria	Micrococcales	Micrococcaceae	NA
12	FALSE	Bacteria	Actinobacteria	Actinobacteria	Actinomycetales	Actinomycetaceae	Arcanobacterium
12	FALSE	Bacteria	Proteobacteria	Alphaproteobacteria	Rhizobiales	Rhizobiaceae	Brucella
12	FALSE	Bacteria	Actinobacteria	Actinobacteria	Micrococcales	Micrococcaceae	Nesterenkonia
12	FALSE	Bacteria	Firmicutes	Clostridia	Clostridiales	Lachnospiraceae	GCA-900066755
12	FALSE	Bacteria	Proteobacteria	Gammaproteobacteria	JTB23	NA	NA
12	FALSE	Bacteria	Firmicutes	Clostridia	Clostridiales	Family_XIII	Anaerovorax
12	FALSE	Bacteria	Kiritimatiellaeota	Kiritimatiellae	WCHB1-41	NA	NA
12	FALSE	Bacteria	Bacteroidetes	Bacteroidia	Cytophagales	Spirosomaceae	Dyadobacter
12	FALSE	Bacteria	Bacteroidetes	Rhodothermia	Rhodothermales	Rhodothermaceae	NA
12	FALSE	Bacteria	Actinobacteria	Actinobacteria	Micrococcales	Microbacteriaceae	Amnibacterium
12	FALSE	Bacteria	Firmicutes	Bacilli	Lactobacillales	Carnobacteriaceae	Granulicatella
12	FALSE	Bacteria	Firmicutes	Bacilli	Lactobacillales	NA	NA
12	FALSE	Bacteria	Actinobacteria	Actinobacteria	Actinomycetales	Actinomycetaceae	Trueperella

12	FALSE	Bacteria	Actinobacteria	Actinobacteria	Micrococcales	Promicromonosporaceae	Cellulosimicrobium
12	FALSE	Bacteria	Firmicutes	Clostridia	Clostridiales	Ruminococcaceae	Harryflintia
12	FALSE	Bacteria	Patescibacteria	Saccharimonadia	Saccharimonadales	Saccharimonadaceae	NA
12	FALSE	Bacteria	Proteobacteria	Gammaproteobacteria	Betaproteobacteriales	Neisseriaceae	Neisseria
12	FALSE	Bacteria	Firmicutes	Clostridia	Clostridiales	Lachnospiraceae	Epulopiscium
12	FALSE	Bacteria	Bacteroidetes	Bacteroidia	Flavobacteriales	Weeksellaceae	NA
12	FALSE	Bacteria	Actinobacteria	Actinobacteria	Micrococcales	Micrococcaceae	Micrococcus
12	FALSE	Bacteria	Firmicutes	Clostridia	Clostridiales	Peptostreptococcaceae	Clostridioides
12	FALSE	Bacteria	Firmicutes	Clostridia	Clostridiales	Lachnospiraceae	Cuneatibacter
12	FALSE	Bacteria	Proteobacteria	Gammaproteobacteria	Pasteurellales	Pasteurellaceae	NA
12	FALSE	Bacteria	Firmicutes	Clostridia	Clostridiales	Ruminococcaceae	Acetanaerobacterium
12	FALSE	Bacteria	Proteobacteria	Alphaproteobacteria	Sphingomonadales	Sphingomonadaceae	NA
12	FALSE	Bacteria	Proteobacteria	Alphaproteobacteria	Rhizobiales	Rhizobiaceae	Paenochrobactrum
12	FALSE	Bacteria	Proteobacteria	Alphaproteobacteria	Rhizobiales	Rhizobiaceae	Shinella
12	FALSE	Bacteria	Actinobacteria	Coriobacteriia	Coriobacteriales	Atopobiaceae	NA
12	FALSE	Bacteria	Firmicutes	Clostridia	Clostridiales	Ruminococcaceae	Candidatus_Soleaferrea
12	FALSE	Bacteria	Bacteroidetes	Bacteroidia	Chitinophagales	Saprospiraceae	NA
12	FALSE	Bacteria	Proteobacteria	Gammaproteobacteria	Betaproteobacteriales	Burkholderiaceae	Parapusillimonas
12	FALSE	Bacteria	Firmicutes	Negativicutes	Selenomonadales	Acidaminococcaceae	Succiniclasticum
12	FALSE	Bacteria	Verrucomicrobia	Verrucomicrobiae	Opitutales	Puniceicoccaceae	NA
12	FALSE	Bacteria	Proteobacteria	Gammaproteobacteria	Betaproteobacteriales	Burkholderiaceae	Ralstonia
12	FALSE	Bacteria	Firmicutes	Bacilli	Bacillales	Paenibacillaceae	Ammoniphilus
12	FALSE	Bacteria	Proteobacteria	Gammaproteobacteria	Betaproteobacteriales	NA	NA
12	FALSE	Bacteria	Proteobacteria	Gammaproteobacteria	Xanthomonadales	Xanthomonadaceae	Thermomonas
12	FALSE	Bacteria	Firmicutes	Clostridia	Clostridiales	Peptococcaceae	Desulfotomaculum
12	FALSE	Archaea	Euryarchaeota	Methanobacteria	Methanobacteriales	Methanobacteriaceae	Methanobacterium
12	FALSE	Bacteria	Actinobacteria	Actinobacteria	Micrococcales	Dermabacteraceae	Dermabacter
12	FALSE	Bacteria	Fusobacteria	Fusobacteriia	Fusobacteriales	Fusobacteriaceae	NA
12	FALSE	Bacteria	Firmicutes	Bacilli	Bacillales	Staphylococcaceae	Jeotgalicoccus
12	FALSE	Bacteria	Planctomycetes	Planctomycetacia	Pirellulales	Pirellulaceae	Rhodopirellula
12	FALSE	Bacteria	Proteobacteria	Alphaproteobacteria	Rhizobiales	Rhizobiaceae	Neorhizobium
12	FALSE	Bacteria	Firmicutes	Clostridia	Clostridiales	Family_XI	NA
12	FALSE	Bacteria	Proteobacteria	Alphaproteobacteria	Rhizobiales	Beijerinckiaceae	Methylobacterium
12	FALSE	Bacteria	Firmicutes	Clostridia	Clostridiales	Peptostreptococcaceae	Paeniclostridium
12	FALSE	Bacteria	Proteobacteria	Gammaproteobacteria	Aeromonadales	Aeromonadaceae	Tolumonas
12	FALSE	Bacteria	Chloroflexi	Dehalococcoidia	SAR202_clade	NA	NA
12	FALSE	Bacteria	Firmicutes	Bacilli	Bacillales	Bacillaceae	Oceanobacillus
12	FALSE	Bacteria	Bacteroidetes	Bacteroidia	Bacteroidales	Dysgonomonadaceae	Proteiniphilum
12	FALSE	Bacteria	Firmicutes	Clostridia	Clostridiales	Eubacteriaceae	Pseudoramibacter
12	FALSE	Bacteria	Proteobacteria	Alphaproteobacteria	Rhizobiales	Xanthobacteraceae	NA
12	FALSE	Bacteria	Firmicutes	Bacilli	Lactobacillales	Aerococcaceae	Aerococcus

12	FALSE	Bacteria	Firmicutes	Bacilli	NA	NA	NA
12	FALSE	Bacteria	Bacteroidetes	NA	NA	NA	NA
12	FALSE	Bacteria	Proteobacteria	Gammaproteobacteria	Xanthomonadales	Rhodanobacteraceae	Rhodanobacter
12	FALSE	Bacteria	Bacteroidetes	Bacteroidia	Flavobacteriales	Weeksellaceae	Moheibacter
12	FALSE	Bacteria	Proteobacteria	Alphaproteobacteria	Rhizobiales	NA	NA
12	FALSE	Bacteria	Firmicutes	Negativicutes	Selenomonadales	Veillonellaceae	Selenomonas_4
12	FALSE	Bacteria	Proteobacteria	Alphaproteobacteria	NA	NA	NA
12	FALSE	Bacteria	Firmicutes	Clostridia	Clostridiales	Defluviitaleaceae	NA
12	FALSE	Bacteria	Bacteroidetes	Bacteroidia	Flavobacteriales	Flavobacteriaceae	NA
12	FALSE	Bacteria	Proteobacteria	Deltaproteobacteria	Myxococcales	mle1-27	NA
12	FALSE	Bacteria	Firmicutes	Clostridia	Clostridiales	Syntrophomonadaceae	NA
12	FALSE	Bacteria	Actinobacteria	Actinobacteria	Actinomycetales	Actinomycetaceae	Actinobaculum
12	FALSE	Bacteria	Bacteroidetes	Bacteroidia	Cytophagales	Spirosomaceae	Rhabdobacter
12	FALSE	Bacteria	Actinobacteria	NA	NA	NA	NA
12	FALSE	Bacteria	Firmicutes	Clostridia	Clostridiales	Ruminococcaceae	Pseudoflavonifractor
12	FALSE	Bacteria	Patescibacteria	Saccharimonadia	Saccharimonadales	NA	NA
12	FALSE	Archaea	Euryarchaeota	Methanobacteria	Methanobacteriales	Methanobacteriaceae	NA
12	FALSE	Bacteria	Firmicutes	Erysipelotrichia	Erysipelotrichales	Erysipelotrichaceae	Asteroleplasma
12	FALSE	Bacteria	Proteobacteria	Alphaproteobacteria	Rhizobiales	Rhizobiaceae	Mesorhizobium
12	FALSE	Bacteria	Proteobacteria	Alphaproteobacteria	Rickettsiales	Mitochondria	NA
12	FALSE	Bacteria	Proteobacteria	Gammaproteobacteria	Betaproteobacteriales	Rhodocyclaceae	Dechloromonas
12	FALSE	Bacteria	Bacteroidetes	Bacteroidia	Chitinophagales	Chitinophagaceae	Flavihumibacter
12	FALSE	Bacteria	Verrucomicrobia	Verrucomicrobiae	NA	NA	NA
11	FALSE	Bacteria	Proteobacteria	Gammaproteobacteria	Enterobacteriales	Enterobacteriaceae	Providencia
11	FALSE	Bacteria	Proteobacteria	Gammaproteobacteria	Aeromonadales	Aeromonadaceae	Aeromonas
11	FALSE	Bacteria	Proteobacteria	Gammaproteobacteria	Betaproteobacteriales	Burkholderiaceae	Achromobacter
11	FALSE	Bacteria	Firmicutes	Clostridia	Clostridiales	Family_XIII	S5-A14a
11	FALSE	Bacteria	Epsilonbacteraeota	Campylobacteria	Campylobacterales	Arcobacteraceae	Arcobacter
11	FALSE	Bacteria	Firmicutes	Clostridia	Clostridiales	Lachnospiraceae	UC5-1-2E3
11	FALSE	Bacteria	Actinobacteria	Actinobacteria	Micrococcales	Micrococcaceae	Pseudoglutamicibacter
11	FALSE	Bacteria	Firmicutes	Clostridia	Clostridiales	Lachnospiraceae	Shuttleworthia
11	FALSE	Bacteria	Proteobacteria	Alphaproteobacteria	Sphingomonadales	Sphingomonadaceae	Sphingopyxis
11	FALSE	Bacteria	Actinobacteria	Actinobacteria	Streptomycetales	Streptomycetaceae	Streptomyces
11	FALSE	Bacteria	Synergistetes	Synergistia	Synergistales	Synergistaceae	Synergistes
11	FALSE	Bacteria	Firmicutes	Clostridia	Clostridiales	Lachnospiraceae	Anaerosporobacter
11	FALSE	Bacteria	Proteobacteria	Gammaproteobacteria	Betaproteobacteriales	Burkholderiaceae	Massilia
11	FALSE	Bacteria	Proteobacteria	Alphaproteobacteria	Rhizobiales	Rhizobiaceae	Pseudochrobactrum
11	FALSE	Bacteria	Bacteroidetes	Bacteroidia	Sphingobacteriales	Sphingobacteriaceae	Nubsella
11	FALSE	Bacteria	Firmicutes	Bacilli	Lactobacillales	Leuconostocaceae	Weissella
11	FALSE	Bacteria	Actinobacteria	Actinobacteria	Bifidobacteriales	Bifidobacteriaceae	Gardnerella
11	FALSE	Bacteria	Proteobacteria	Alphaproteobacteria	Caulobacterales	Caulobacteraceae	Caulobacter

11	FALSE	Bacteria	Firmicutes	Erysipelotrichia	Erysipelotrichales	Erysipelotrichaceae	Solobacterium
11	FALSE	Bacteria	Actinobacteria	Coriobacteriia	Coriobacteriales	Eggerthellaceae	Senegalimassilia
11	FALSE	Bacteria	Firmicutes	Clostridia	Clostridiales	Ruminococcaceae	Ruminococcaceae_UCG-011
11	FALSE	Bacteria	Proteobacteria	Gammaproteobacteria	Betaproteobacteriales	Burkholderiaceae	Cupriavidus
11	FALSE	Bacteria	Firmicutes	Clostridia	Clostridiales	Defluviitaleaceae	Defluviitaleaceae_UCG-011
11	FALSE	Bacteria	Actinobacteria	Actinobacteria	Micrococcales	Micrococcaceae	Rothia
11	FALSE	Bacteria	Proteobacteria	Alphaproteobacteria	Sphingomonadales	Sphingomonadaceae	Novosphingobium
11	FALSE	Bacteria	Proteobacteria	Gammaproteobacteria	Betaproteobacteriales	Burkholderiaceae	Aquabacterium
11	FALSE	Bacteria	Actinobacteria	Actinobacteria	Micrococcales	Microbacteriaceae	NA
11	FALSE	Bacteria	Verrucomicrobia	Verrucomicrobiae	Verrucomicrobiales	NA	NA
10	FALSE	Bacteria	Proteobacteria	Gammaproteobacteria	Aeromonadales	Succinivibrionaceae	Succinivibrio
10	FALSE	Bacteria	Proteobacteria	Gammaproteobacteria	Aeromonadales	Succinivibrionaceae	NA
10	FALSE	Bacteria	Firmicutes	Clostridia	Clostridiales	Peptococcaceae	NA
10	FALSE	Bacteria	Firmicutes	Clostridia	Clostridiales	Lachnospiraceae	Tyzzerella_3
10	FALSE	Bacteria	Firmicutes	Bacilli	Bacillales	Planococcaceae	Lysinibacillus
10	FALSE	Bacteria	Fusobacteria	Fusobacteriia	Fusobacteriales	Leptotrichiaceae	Sneathia
10	FALSE	Bacteria	Proteobacteria	Alphaproteobacteria	Sphingomonadales	Sphingomonadaceae	Sphingobium
10	FALSE	Bacteria	Firmicutes	Clostridia	Clostridiales	Ruminococcaceae	Caproiciproducens
10	FALSE	Bacteria	Actinobacteria	Actinobacteria	Bifidobacteriales	Bifidobacteriaceae	Alloscardovia
10	FALSE	Bacteria	Actinobacteria	Coriobacteriia	Coriobacteriales	Eggerthellaceae	Adlercreutzia
10	FALSE	Bacteria	Bacteroidetes	Bacteroidia	Bacteroidales	Rikenellaceae	Millionella
10	FALSE	Bacteria	Firmicutes	Clostridia	Clostridiales	Clostridiaceae_1	Sarcina
10	FALSE	Bacteria	Lentisphaerae	Lentisphaeria	Victivallales	NA	NA
10	FALSE	Bacteria	Synergistetes	Synergistia	Synergistales	Synergistaceae	Pyramidobacter
10	FALSE	Bacteria	Synergistetes	Synergistia	Synergistales	Synergistaceae	Jonquetella
10	FALSE	Bacteria	Actinobacteria	Coriobacteriia	Coriobacteriales	Atopobiaceae	Olsenella
10	FALSE	Bacteria	Firmicutes	Bacilli	Bacillales	Family_XI	Gemella
10	FALSE	Bacteria	Elusimicrobia	Elusimicrobia	Elusimicrobiales	Elusimicrobiaceae	Elusimicrobium
10	FALSE	Bacteria	Proteobacteria	Alphaproteobacteria	Rhodobacterales	Rhodobacteraceae	Paracoccus
10	FALSE	Bacteria	Firmicutes	Negativicutes	Selenomonadales	Acidaminococcaceae	NA
10	FALSE	Bacteria	Firmicutes	Negativicutes	Selenomonadales	NA	NA
10	FALSE	Bacteria	Proteobacteria	Gammaproteobacteria	Enterobacteriales	Enterobacteriaceae	Cosenzaea
10	FALSE	Bacteria	Actinobacteria	Actinobacteria	Corynebacteriales	NA	NA
10	FALSE	Bacteria	Proteobacteria	NA	NA	NA	NA
10	FALSE	Bacteria	Proteobacteria	Alphaproteobacteria	Rhodobacterales	Rhodobacteraceae	NA
10	FALSE	Bacteria	Firmicutes	Bacilli	Lactobacillales	Streptococcaceae	NA
9	FALSE	Bacteria	Bacteroidetes	Bacteroidia	Bacteroidales	Bacteroidaceae	Bacteroides
9	FALSE	Bacteria	Bacteroidetes	Bacteroidia	Bacteroidales	Prevotellaceae	Alloprevotella
9	FALSE	Bacteria	Firmicutes	Clostridia	Clostridiales	Lachnospiraceae	Lachnospiraceae_UCG-003
9	FALSE	Bacteria	Bacteroidetes	Bacteroidia	Bacteroidales	Barnesiellaceae	Coprobacter
9	FALSE	Bacteria	Firmicutes	Clostridia	Clostridiales	Family_XI	Murdochiella

9	FALSE	Bacteria	Proteobacteria	Gammaproteobacteria	Betaproteobacteriales	Burkholderiaceae	Ottowia
9	FALSE	Bacteria	Firmicutes	Erysipelotrichia	Erysipelotrichales	Erysipelotrichaceae	Merdibacter
9	FALSE	Bacteria	Firmicutes	Clostridia	DTU014	NA	NA
9	FALSE	Bacteria	Firmicutes	Clostridia	Clostridiales	Ruminococcaceae	Anaerofilum
9	FALSE	Archaea	Euryarchaeota	Methanobacteria	Methanobacteriales	Methanobacteriaceae	Methanosphaera
9	FALSE	Bacteria	Bacteroidetes	Bacteroidia	Bacteroidales	Rikenellaceae	NA
9	FALSE	Bacteria	Actinobacteria	Actinobacteria	Corynebacteriales	Corynebacteriaceae	Corynebacterium
8	FALSE	Bacteria	Firmicutes	Clostridia	Clostridiales	Ruminococcaceae	Ruminococcaceae_UCG-005
8	FALSE	Bacteria	Proteobacteria	Gammaproteobacteria	Betaproteobacteriales	Burkholderiaceae	Variovorax
8	FALSE	Bacteria	Proteobacteria	Gammaproteobacteria	Betaproteobacteriales	Burkholderiaceae	Oligella
8	FALSE	Bacteria	Proteobacteria	Gammaproteobacteria	Xanthomonadales	Xanthomonadaceae	NA
8	FALSE	Bacteria	Bacteroidetes	Bacteroidia	Bacteroidales	Muribaculaceae	CAG-873
8	FALSE	Bacteria	Proteobacteria	Alphaproteobacteria	Rhizobiales	Rhizobiaceae	NA
8	FALSE	Bacteria	Firmicutes	Bacilli	Bacillales	Paenibacillaceae	Brevibacillus
8	FALSE	Bacteria	Proteobacteria	Gammaproteobacteria	Betaproteobacteriales	Burkholderiaceae	Oxalobacter
8	FALSE	Bacteria	Firmicutes	Clostridia	Clostridiales	Christensenellaceae	NA
8	FALSE	Bacteria	Firmicutes	Bacilli	Bacillales	Planococcaceae	Rummeliibacillus
8	FALSE	Bacteria	Firmicutes	Erysipelotrichia	Erysipelotrichales	Erysipelotrichaceae	Faecalicoccus
8	FALSE	Bacteria	Firmicutes	Clostridia	Clostridiales	Ruminococcaceae	Ruminococcaceae_UCG-009
8	FALSE	Bacteria	Firmicutes	Clostridia	Clostridiales	Clostridiaceae_1	Clostridium_sensu_stricto_13
8	FALSE	Bacteria	Firmicutes	Clostridia	Clostridiales	Lachnospiraceae	Cellulosilyticum
8	FALSE	Bacteria	Proteobacteria	Deltaproteobacteria	Desulfovibrionales	Desulfovibrionaceae	Mailhella
8	FALSE	Bacteria	Actinobacteria	Coriobacteriia	Coriobacteriales	Eggerthellaceae	Gordonibacter
8	FALSE	Bacteria	Firmicutes	Clostridia	Clostridiales	Lachnospiraceae	Oribacterium
8	FALSE	Bacteria	Firmicutes	Bacilli	Bacillales	Staphylococcaceae	Nosocomiicoccus
8	FALSE	Bacteria	Actinobacteria	Coriobacteriia	Coriobacteriales	NA	NA
8	FALSE	Bacteria	Lentisphaerae	Lentisphaeria	Victivallales	Victivallaceae	NA
8	FALSE	Bacteria	Firmicutes	Erysipelotrichia	Erysipelotrichales	Erysipelotrichaceae	Allobaculum
8	FALSE	Bacteria	Proteobacteria	Alphaproteobacteria	Sphingomonadales	Sphingomonadaceae	Sphingomonas
8	FALSE	Bacteria	Firmicutes	Clostridia	Clostridiales	Clostridiaceae_1	NA
7	FALSE	Bacteria	Firmicutes	Clostridia	Clostridiales	Lachnospiraceae	Dorea
7	FALSE	Bacteria	Bacteroidetes	Bacteroidia	Bacteroidales	Prevotellaceae	Prevotellaceae_NK3B31_group
7	FALSE	Bacteria	Proteobacteria	Deltaproteobacteria	Desulfovibrionales	Desulfovibrionaceae	Desulfovibrio
7	FALSE	Bacteria	Firmicutes	Erysipelotrichia	Erysipelotrichales	Erysipelotrichaceae	Erysipelotrichaceae_UCG-004
7	FALSE	Bacteria	Firmicutes	Erysipelotrichia	Erysipelotrichales	Erysipelotrichaceae	Candidatus_Stoquefichus
7	FALSE	Bacteria	Firmicutes	Erysipelotrichia	Erysipelotrichales	Erysipelotrichaceae	Holdemania
7	FALSE	Bacteria	Proteobacteria	Gammaproteobacteria	Betaproteobacteriales	Burkholderiaceae	Acidovorax
7	FALSE	Bacteria	Firmicutes	Clostridia	Clostridiales	Lachnospiraceae	GCA-900066575
7	FALSE	Bacteria	Firmicutes	Clostridia	Clostridiales	Family_XI	W5053
7	FALSE	Bacteria	Firmicutes	Negativicutes	Selenomonadales	Veillonellaceae	Negativicoccus
7	FALSE	Bacteria	Firmicutes	Negativicutes	Selenomonadales	Veillonellaceae	Anaeroglobus

7	FALSE	Bacteria	Bacteroidetes	Bacteroidia	Bacteroidales	Marinifilaceae	Sanguibacteroides
7	FALSE	Bacteria	Firmicutes	Clostridia	Clostridiales	Peptostreptococcaceae	NA
6	FALSE	Bacteria	Firmicutes	Clostridia	Clostridiales	Ruminococcaceae	Intestinimonas
6	FALSE	Bacteria	Actinobacteria	Actinobacteria	Corynebacteriales	Corynebacteriaceae	NA
6	FALSE	Bacteria	Firmicutes	Bacilli	Lactobacillales	Lactobacillaceae	Pediococcus
6	FALSE	Bacteria	Firmicutes	Clostridia	Clostridiales	Ruminococcaceae	Fastidiosipila
6	FALSE	Bacteria	Firmicutes	Clostridia	Clostridiales	Family_XI	Gallicola
6	FALSE	Archaea	Euryarchaeota	Thermoplasmata	Methanomassiliicoccales	Methanomassiliicoccaceae	Methanomassiliicoccus
5	FALSE	Bacteria	Firmicutes	Clostridia	Clostridiales	Lachnospiraceae	Lachnoclostridium
5	FALSE	Bacteria	Bacteroidetes	Bacteroidia	Flavobacteriales	Flavobacteriaceae	Flavobacterium
5	FALSE	Bacteria	Proteobacteria	Gammaproteobacteria	Betaproteobacteriales	Burkholderiaceae	NA
5	FALSE	Bacteria	Firmicutes	Clostridia	Clostridiales	Clostridiales_vadinBB60_group	NA
5	FALSE	Bacteria	Firmicutes	Negativicutes	Selenomonadales	Veillonellaceae	Megamonas
5	FALSE	Bacteria	Firmicutes	Erysipelotrichia	Erysipelotrichales	Erysipelotrichaceae	Erysipelatoclostridium
5	FALSE	Bacteria	Firmicutes	Negativicutes	Selenomonadales	Veillonellaceae	NA
5	FALSE	Bacteria	Bacteroidetes	Bacteroidia	Bacteroidales	Dysgonomonadaceae	Dysgonomonas
5	FALSE	Bacteria	Firmicutes	Clostridia	Clostridiales	Peptostreptococcaceae	Terrisporobacter
5	FALSE	Bacteria	Firmicutes	Clostridia	Clostridiales	Ruminococcaceae	Ruminococcaceae_UCG-010
5	FALSE	Bacteria	Bacteroidetes	Bacteroidia	Bacteroidales	Barnesiellaceae	NA
5	FALSE	Bacteria	Firmicutes	Clostridia	Clostridiales	Ruminococcaceae	GCA-900066225
5	FALSE	Bacteria	Proteobacteria	Gammaproteobacteria	Xanthomonadales	Xanthomonadaceae	Pseudoxanthomonas
5	FALSE	Bacteria	Firmicutes	Clostridia	Clostridiales	Lachnospiraceae	Marvinbryantia
5	FALSE	Bacteria	Firmicutes	Bacilli	Lactobacillales	Streptococcaceae	Lactococcus
5	FALSE	Bacteria	Actinobacteria	Coriobacteriia	Coriobacteriales	Eggerthellaceae	NA
5	FALSE	Bacteria	Firmicutes	Clostridia	Clostridiales	Ruminococcaceae	Angelakisella
5	FALSE	Bacteria	Firmicutes	Negativicutes	Selenomonadales	Veillonellaceae	Allisonella
5	FALSE	Bacteria	Firmicutes	Clostridia	Clostridiales	Peptococcaceae	Peptococcus
5	FALSE	Bacteria	Firmicutes	Bacilli	Lactobacillales	Lactobacillaceae	NA
5	FALSE	Archaea	Euryarchaeota	Thermoplasmata	Methanomassiliicoccales	Methanomethylophilaceae	NA
5	FALSE	Bacteria	Firmicutes	Clostridia	Clostridiales	Lachnospiraceae	Howardella
5	FALSE	Bacteria	Actinobacteria	Actinobacteria	Micrococcales	Brevibacteriaceae	Brevibacterium
5	FALSE	Bacteria	Actinobacteria	Actinobacteria	Actinomycetales	Actinomycetaceae	Actinomyces
5	FALSE	Bacteria	Firmicutes	Clostridia	Clostridiales	Eubacteriaceae	Eubacterium
5	FALSE	Bacteria	Actinobacteria	Coriobacteriia	Coriobacteriales	Atopobiaceae	Atopobium
5	FALSE	Bacteria	Actinobacteria	Coriobacteriia	Coriobacteriales	Eggerthellaceae	Slackia
4	FALSE	Bacteria	Firmicutes	Clostridia	Clostridiales	Ruminococcaceae	Oscillibacter
4	FALSE	Bacteria	Firmicutes	Clostridia	Clostridiales	Clostridiaceae_1	Clostridium_sensu_stricto_1
4	FALSE	Bacteria	Firmicutes	Clostridia	Clostridiales	Family_XI	Finegoldia
4	FALSE	Bacteria	Firmicutes	Clostridia	Clostridiales	Family_XIII	Family_XIII_AD3011_group
4	FALSE	Bacteria	Firmicutes	Clostridia	Clostridiales	Lachnospiraceae	CAG-56
4	FALSE	Bacteria	Firmicutes	Erysipelotrichia	Erysipelotrichales	Erysipelotrichaceae	Dielma

4	FALSE	Bacteria	Firmicutes	Clostridia	Clostridiales	Ruminococcaceae	Ruminiclostridium_1
4	FALSE	Bacteria	Firmicutes	Clostridia	Clostridiales	Family_XIII	NA
4	FALSE	Bacteria	Bacteroidetes	Bacteroidia	Sphingobacteriales	Sphingobacteriaceae	Sphingobacterium
4	FALSE	Bacteria	Bacteroidetes	Bacteroidia	NA	NA	NA
4	FALSE	Bacteria	Lentisphaerae	Lentisphaeria	Victivallales	Victivallaceae	Victivallis
4	FALSE	Bacteria	Actinobacteria	Coriobacteriia	Coriobacteriales	Coriobacteriales_Incertae_Sedis	NA
4	FALSE	Bacteria	Bacteroidetes	Bacteroidia	Flavobacteriales	Weeksellaceae	Cloacibacterium
4	FALSE	Bacteria	Firmicutes	Clostridia	Clostridiales	Ruminococcaceae	Hydrogenoanaerobacterium
4	FALSE	Bacteria	Actinobacteria	Coriobacteriia	Coriobacteriales	Eggerthellaceae	Eggerthella
4	FALSE	Bacteria	Bacteroidetes	Bacteroidia	Bacteroidales	Prevotellaceae	NA
4	FALSE	Bacteria	Firmicutes	NA	NA	NA	NA
4	FALSE	Bacteria	Firmicutes	Clostridia	Clostridiales	Family_XIII	Mogibacterium
4	FALSE	Bacteria	Firmicutes	Bacilli	Bacillales	Staphylococcaceae	Staphylococcus
4	FALSE	Bacteria	Proteobacteria	Gammaproteobacteria	Pseudomonadales	Pseudomonadaceae	NA
3	FALSE	Bacteria	Proteobacteria	Gammaproteobacteria	Enterobacteriales	Enterobacteriaceae	Proteus
3	FALSE	Bacteria	Firmicutes	Negativicutes	Selenomonadales	Acidaminococcaceae	Acidaminococcus
3	FALSE	Bacteria	Firmicutes	Clostridia	Clostridiales	Christensenellaceae	Christensenellaceae_R-7_group
3	FALSE	Bacteria	Firmicutes	Clostridia	Clostridiales	Ruminococcaceae	Ruminococcaceae_UCG-003
3	FALSE	Bacteria	Proteobacteria	Gammaproteobacteria	Enterobacteriales	Enterobacteriaceae	Morganella
3	FALSE	Bacteria	Firmicutes	Clostridia	Clostridiales	Ruminococcaceae	Ruminococcaceae_NK4A214_group
3	FALSE	Bacteria	Bacteroidetes	Bacteroidia	Bacteroidales	Marinifilaceae	Odoribacter
3	FALSE	Bacteria	Firmicutes	Clostridia	Clostridiales	Peptostreptococcaceae	Intestinibacter
3	FALSE	Bacteria	Firmicutes	Clostridia	Clostridiales	Ruminococcaceae	CAG-352
3	FALSE	Bacteria	Bacteroidetes	Bacteroidia	Bacteroidales	Prevotellaceae	Prevotella_7
3	FALSE	Bacteria	Bacteroidetes	Bacteroidia	Bacteroidales	Rikenellaceae	Rikenellaceae_RC9_gut_group
3	FALSE	Bacteria	Firmicutes	Erysipelotrichia	Erysipelotrichales	Erysipelotrichaceae	Catenibacterium
3	FALSE	Bacteria	Firmicutes	Clostridia	Clostridiales	Lachnospiraceae	Coprococcus_2
3	FALSE	Bacteria	Firmicutes	Erysipelotrichia	Erysipelotrichales	Erysipelotrichaceae	Faecalitalea
3	FALSE	Bacteria	Bacteroidetes	Bacteroidia	Bacteroidales	Marinifilaceae	Butyricimonas
3	FALSE	Bacteria	Bacteroidetes	Bacteroidia	Flavobacteriales	Weeksellaceae	Chryseobacterium
3	FALSE	Bacteria	Bacteroidetes	Bacteroidia	Bacteroidales	Prevotellaceae	Prevotella_2
3	FALSE	Bacteria	Firmicutes	Negativicutes	Selenomonadales	Veillonellaceae	Veillonella
3	FALSE	Bacteria	Firmicutes	Bacilli	Bacillales	Planococcaceae	NA
3	FALSE	Bacteria	Firmicutes	Bacilli	Bacillales	NA	NA
3	FALSE	Bacteria	Lentisphaerae	Lentisphaeria	Victivallales	vadinBE97	NA
3	FALSE	Bacteria	Proteobacteria	Deltaproteobacteria	Desulfovibrionales	Desulfovibrionaceae	NA
2	FALSE	Bacteria	Firmicutes	Negativicutes	Selenomonadales	Acidaminococcaceae	Phascolarctobacterium
2	FALSE	Bacteria	Firmicutes	Clostridia	Clostridiales	Peptostreptococcaceae	Romboutsia
2	FALSE	Bacteria	Firmicutes	Clostridia	Clostridiales	Ruminococcaceae	Ruminiclostridium_5
2	FALSE	Bacteria	Firmicutes	Erysipelotrichia	Erysipelotrichales	Erysipelotrichaceae	Turicibacter
2	FALSE	Bacteria	Proteobacteria	Gammaproteobacteria	Xanthomonadales	Xanthomonadaceae	Stenotrophomonas

2	FALSE	Bacteria	Firmicutes	Clostridia	Clostridiales	Lachnospiraceae	Lachnospiraceae_UCG-001
2	FALSE	Bacteria	Firmicutes	Clostridia	Clostridiales	Ruminococcaceae	DTU089
2	FALSE	Bacteria	Proteobacteria	Gammaproteobacteria	NA	NA	NA
1	FALSE	Bacteria	Bacteroidetes	Bacteroidia	Bacteroidales	Tannerellaceae	Parabacteroides
1	FALSE	Bacteria	Firmicutes	Negativicutes	Selenomonadales	Veillonellaceae	Dialister
1	FALSE	Bacteria	Firmicutes	Clostridia	Clostridiales	Ruminococcaceae	Ruminococcaceae_UCG-002
1	FALSE	Bacteria	Proteobacteria	Gammaproteobacteria	Betaproteobacteriales	Burkholderiaceae	Parasutterella
1	FALSE	Bacteria	Firmicutes	Clostridia	Clostridiales	Lachnospiraceae	Butyrivibrio
1	FALSE	Bacteria	Firmicutes	Clostridia	Clostridiales	Lachnospiraceae	Lachnospiraceae_NK4A136_group
1	FALSE	Bacteria	Firmicutes	Clostridia	Clostridiales	Lachnospiraceae	Eisenbergiella
1	FALSE	Bacteria	Firmicutes	Erysipelotrichia	Erysipelotrichales	Erysipelotrichaceae	Holdemanella
1	FALSE	Bacteria	Actinobacteria	Coriobacteriia	Coriobacteriales	Coriobacteriaceae	Collinsella
1	FALSE	Bacteria	Tenericutes	Mollicutes	Izimaplasmatales	NA	NA
1	FALSE	Bacteria	Fusobacteria	Fusobacteriia	Fusobacteriales	Fusobacteriaceae	Fusobacterium
1	FALSE	Bacteria	Proteobacteria	Alphaproteobacteria	Caulobacterales	Caulobacteraceae	Brevundimonas
1	FALSE	Bacteria	Firmicutes	Clostridia	Clostridiales	Lachnospiraceae	Coprococcus_1
1	FALSE	Bacteria	Cyanobacteria	Melainabacteria	Gastranaerophilales	NA	NA
1	FALSE	Bacteria	NA	NA	NA	NA	NA
0	FALSE	Bacteria	Proteobacteria	Gammaproteobacteria	Enterobacteriales	Enterobacteriaceae	Escherichia/Shigella
0	FALSE	Bacteria	Proteobacteria	Gammaproteobacteria	Enterobacteriales	Enterobacteriaceae	NA
0	FALSE	Bacteria	Firmicutes	Clostridia	Clostridiales	Ruminococcaceae	Ruminococcus_2
0	FALSE	Bacteria	Firmicutes	Clostridia	Clostridiales	Ruminococcaceae	Subdoligranulum
0	FALSE	Bacteria	Bacteroidetes	Bacteroidia	Bacteroidales	Prevotellaceae	Prevotella_9
0	FALSE	Bacteria	Proteobacteria	Gammaproteobacteria	Pseudomonadales	Pseudomonadaceae	Pseudomonas
0	FALSE	Bacteria	Proteobacteria	Gammaproteobacteria	Pseudomonadales	Moraxellaceae	Acinetobacter
0	FALSE	Bacteria	Firmicutes	Clostridia	Clostridiales	Lachnospiraceae	Tyzzerella_4
0	FALSE	Bacteria	Proteobacteria	Gammaproteobacteria	Enterobacteriales	Enterobacteriaceae	Klebsiella
0	FALSE	Bacteria	Firmicutes	Clostridia	Clostridiales	Ruminococcaceae	Ruminiclostridium_6
0	FALSE	Bacteria	Firmicutes	Clostridia	Clostridiales	Ruminococcaceae	Ruminococcus_1
0	FALSE	Bacteria	Firmicutes	Erysipelotrichia	Erysipelotrichales	Erysipelotrichaceae	Erysipelotrichaceae_UCG-003
0	FALSE	Bacteria	Firmicutes	Clostridia	Clostridiales	Ruminococcaceae	Flavonifractor
0	FALSE	Bacteria	Firmicutes	Bacilli	Lactobacillales	Streptococcaceae	Streptococcus
0	FALSE	Bacteria	Firmicutes	Clostridia	Clostridiales	Lachnospiraceae	Tyzzerella
0	FALSE	Bacteria	Proteobacteria	Gammaproteobacteria	Betaproteobacteriales	Burkholderiaceae	Delftia
0	FALSE	Bacteria	Proteobacteria	Gammaproteobacteria	Betaproteobacteriales	Burkholderiaceae	Sutterella
0	FALSE	Bacteria	Bacteroidetes	Bacteroidia	Bacteroidales	Barnesiellaceae	Barnesiella
0	FALSE	Bacteria	Proteobacteria	Alphaproteobacteria	Rhodospirillales	NA	NA
0	FALSE	Bacteria	Firmicutes	Clostridia	Clostridiales	Ruminococcaceae	Ruminiclostridium_9
0	FALSE	Bacteria	Firmicutes	Clostridia	Clostridiales	Ruminococcaceae	Ruminococcaceae_UCG-014
0	FALSE	Bacteria	Bacteroidetes	Bacteroidia	Bacteroidales	Prevotellaceae	Paraprevotella
0	FALSE	Bacteria	Firmicutes	Clostridia	Clostridiales	Ruminococcaceae	Negativibacillus

0	FALSE	Bacteria	Tenericutes	Mollicutes	Mollicutes_RF39	NA	NA
0	FALSE	Bacteria	Firmicutes	Bacilli	Lactobacillales	Enterococcaceae	Enterococcus
0	FALSE	Bacteria	Bacteroidetes	Bacteroidia	Bacteroidales	Muribaculaceae	NA
0	FALSE	Bacteria	Firmicutes	Clostridia	Clostridiales	Lachnospiraceae	Lachnospiraceae_UCG-010

Supplementary Table 3. MWAS of dataset 2 conducted using ANCOM

Sample size for ANCOM included subset of samples that had complete data on all covariates tested: N= 306 cases and 177 controls in dataset 2.

W= ANCOM score indicating the number of times a genus achieved FDR<0.05 as compared to other genera (maximum W possible: 444 in dataset 1, 560 in dataset 2).

0.8= Threshold at which results were considered significant (TRUE).

W	0.8	Kingdom	Phylum	Class	Order	Family	Genus
553	TRUE	Bacteria	Actinobacteria	Actinobacteria	Bifidobacteriales	Bifidobacteriaceae	Bifidobacterium
545	TRUE	Bacteria	Firmicutes	Clostridia	Clostridiales	Lachnospiraceae	Agathobacter
544	TRUE	Bacteria	Firmicutes	Clostridia	Clostridiales	Lachnospiraceae	Lachnospiraceae_UCG-004
541	TRUE	Bacteria	Firmicutes	Clostridia	Clostridiales	Lachnospiraceae	Roseburia
541	TRUE	Bacteria	Firmicutes	Clostridia	Clostridiales	Ruminococcaceae	Ruminococcaceae_UCG-013
538	TRUE	Bacteria	Firmicutes	Clostridia	Clostridiales	Lachnospiraceae	Lachnospiraceae_ND3007_group
536	TRUE	Bacteria	Firmicutes	Clostridia	Clostridiales	Lachnospiraceae	Anaerostipes
535	TRUE	Bacteria	Firmicutes	Clostridia	Clostridiales	Ruminococcaceae	Faecalibacterium
533	TRUE	Bacteria	Firmicutes	Clostridia	Clostridiales	Lachnospiraceae	Blautia
530	TRUE	Bacteria	Firmicutes	Clostridia	Clostridiales	Eubacteriaceae	Eubacterium
525	TRUE	Bacteria	Firmicutes	Clostridia	Clostridiales	Ruminococcaceae	Oscillospira
524	TRUE	Bacteria	Firmicutes	Clostridia	Clostridiales	Ruminococcaceae	Ruminococcus_2
521	TRUE	Bacteria	Firmicutes	Clostridia	Clostridiales	Lachnospiraceae	Fusicatenibacter
521	TRUE	Bacteria	Firmicutes	Clostridia	Clostridiales	Lachnospiraceae	Lachnospira
521	TRUE	Bacteria	Firmicutes	Clostridia	Clostridiales	Ruminococcaceae	Ruminiclostridium_6
521	TRUE	Bacteria	Firmicutes	Clostridia	Clostridiales	Ruminococcaceae	Ruminococcus_1
505	TRUE	Bacteria	Firmicutes	Clostridia	Clostridiales	Ruminococcaceae	Butyricicoccus
505	TRUE	Bacteria	Proteobacteria	Gammaproteobacteria	Pseudomonadales	Pseudomonadaceae	Pseudomonas
503	TRUE	Bacteria	Firmicutes	Clostridia	Clostridiales	Lachnospiraceae	Lachnospiraceae_UCG-001
496	TRUE	Bacteria	Actinobacteria	Actinobacteria	Corynebacteriales	Corynebacteriaceae	Lawsonella
493	TRUE	Bacteria	Proteobacteria	Deltaproteobacteria	Desulfovibrionales	Desulfovibrionaceae	Desulfovibrio
493	TRUE	Bacteria	Firmicutes	Erysipelotrichia	Erysipelotrichales	Erysipelotrichaceae	Turicibacter
491	TRUE	Archaea	Euryarchaeota	Methanobacteria	Methanobacteriales	Methanobacteriaceae	Methanobrevibacter
479	TRUE	Bacteria	Firmicutes	Clostridia	Clostridiales	Ruminococcaceae	DTU089
477	TRUE	Bacteria	Firmicutes	Erysipelotrichia	Erysipelotrichales	Erysipelotrichaceae	Erysipelotrichaceae_UCG-003
468	TRUE	Bacteria	Bacteroidetes	Bacteroidia	Bacteroidales	Porphyromonadaceae	Porphyromonas

465	TRUE	Bacteria	Actinobacteria	Actinobacteria	Corynebacteriales	Corynebacteriaceae	Corynebacterium_1
463	TRUE	Bacteria	Bacteroidetes	Bacteroidia	Bacteroidales	Prevotellaceae	Prevotella
459	TRUE	Bacteria	Firmicutes	Clostridia	Clostridiales	Lachnospiraceae	Lachnoclostridium
458	TRUE	Bacteria	Firmicutes	Bacilli	Lactobacillales	Lactobacillaceae	Lactobacillus
458	TRUE	Bacteria	Firmicutes	Clostridia	Clostridiales	Ruminococcaceae	Ruminococcaceae_UCG-014
454	TRUE	Bacteria	Firmicutes	Negativicutes	Selenomonadales	Veillonellaceae	Veillonella
452	TRUE	Bacteria	Firmicutes	Clostridia	Clostridiales	Lachnospiraceae	NA
449	TRUE	Bacteria	Firmicutes	Clostridia	Clostridiales	Ruminococcaceae	Candidatus_Soleaferrea
440	FALSE	Bacteria	Proteobacteria	Gammaproteobacteria	Pseudomonadales	Moraxellaceae	Acinetobacter
439	FALSE	Bacteria	Firmicutes	Erysipelotrichia	Erysipelotrichales	Erysipelotrichaceae	NA
438	FALSE	Bacteria	Firmicutes	Clostridia	Clostridiales	Ruminococcaceae	Intestinimonas
433	FALSE	Bacteria	Firmicutes	Clostridia	Clostridiales	Ruminococcaceae	Ruminiclostridium_9
428	FALSE	Bacteria	Firmicutes	Clostridia	Clostridiales	Family_XI	Anaerococcus
425	FALSE	Bacteria	Firmicutes	Erysipelotrichia	Erysipelotrichales	Erysipelotrichaceae	Erysipelatoclostridium
422	FALSE	Bacteria	Firmicutes	Clostridia	Clostridiales	Family_XIII	Family_XIII_UCG-001
411	FALSE	Bacteria	Firmicutes	Clostridia	Clostridiales	Ruminococcaceae	Phocea
408	FALSE	Bacteria	Cyanobacteria	Oxyphotobacteria	Chloroplast	NA	NA
408	FALSE	Bacteria	Firmicutes	Clostridia	Clostridiales	Family_XIII	S5-A14a
407	FALSE	Bacteria	Bacteroidetes	Bacteroidia	Bacteroidales	Bacteroidaceae	Bacteroides
405	FALSE	Bacteria	Firmicutes	Erysipelotrichia	Erysipelotrichales	Erysipelotrichaceae	Holdemania
399	FALSE	Bacteria	Firmicutes	Bacilli	Lactobacillales	Carnobacteriaceae	Granulicatella
395	FALSE	Bacteria	Firmicutes	Clostridia	Clostridiales	Lachnospiraceae	Cuneatibacter
388	FALSE	Bacteria	Firmicutes	Clostridia	Clostridiales	Lachnospiraceae	Lachnospiraceae_UCG-003
386	FALSE	Bacteria	Firmicutes	Clostridia	Clostridiales	Lachnospiraceae	GCA-900066575
364	FALSE	Bacteria	Proteobacteria	Gammaproteobacteria	Betaproteobacteriales	Burkholderiaceae	Delftia
356	FALSE	Bacteria	Firmicutes	Clostridia	Clostridiales	Family_XI	Peptoniphilus
350	FALSE	Bacteria	Firmicutes	Clostridia	Clostridiales	Lachnospiraceae	Lachnospiraceae_UCG-008
348	FALSE	Bacteria	Firmicutes	Clostridia	Clostridiales	Family_XI	Parvimonas
344	FALSE	Bacteria	Firmicutes	Bacilli	Bacillales	Family_XI	Gemella
307	FALSE	Bacteria	Actinobacteria	Actinobacteria	Actinomycetales	Actinomycetaceae	Varibaculum
299	FALSE	Bacteria	Proteobacteria	Gammaproteobacteria	Xanthomonadales	Xanthomonadaceae	Stenotrophomonas
268	FALSE	Bacteria	Firmicutes	Bacilli	Bacillales	Bacillaceae	Bacillus
218	FALSE	Bacteria	Firmicutes	Negativicutes	Selenomonadales	Veillonellaceae	Selenomonas_3
209	FALSE	Bacteria	Firmicutes	Erysipelotrichia	Erysipelotrichales	Erysipelotrichaceae	Asteroleplasma
62	FALSE	Bacteria	Lentisphaerae	Lentisphaeria	Victivallales	Victivallaceae	Victivallis

58	FALSE	Bacteria	Actinobacteria	Coriobacteriia	Coriobacteriales	Atopobiaceae	Olsenella
54	FALSE	Bacteria	Actinobacteria	Actinobacteria	Propionibacteriales	Propionibacteriaceae	Tessaracoccus
50	FALSE	Bacteria	Epsilonbacteraeota	Campylobacteria	Campylobacterales	Campylobacteraceae	Campylobacter
49	FALSE	Bacteria	Firmicutes	Clostridia	Clostridiales	Family_XI	Ezakiella
47	FALSE	Bacteria	Proteobacteria	Alphaproteobacteria	Rhizobiales	Beijerinckiaceae	Bosea
47	FALSE	Bacteria	Proteobacteria	Alphaproteobacteria	Rhizobiales	Beijerinckiaceae	Methylobacterium
46	FALSE	Bacteria	Firmicutes	Clostridia	Clostridiales	Peptostreptococcaceae	NA
44	FALSE	Bacteria	Actinobacteria	Actinobacteria	Propionibacteriales	Propionibacteriaceae	Cutibacterium
44	FALSE	Bacteria	Proteobacteria	Alphaproteobacteria	Rhizobiales	Rhizobiaceae	NA
43	FALSE	Bacteria	Firmicutes	Clostridia	Clostridiales	Clostridiaceae_1	Clostridium_sensu_stricto_13
41	FALSE	Bacteria	Firmicutes	Bacilli	Lactobacillales	Lactobacillaceae	Pediococcus
40	FALSE	Bacteria	Firmicutes	Clostridia	Clostridiales	Ruminococcaceae	Fastidiosipila
40	FALSE	Bacteria	Actinobacteria	Actinobacteria	Actinomycetales	Actinomycetaceae	Mobiluncus
40	FALSE	Bacteria	Actinobacteria	Actinobacteria	Micrococcales	Microbacteriaceae	Pseudoclavibacter
39	FALSE	Bacteria	Firmicutes	Clostridia	Clostridiales	Family_XI	Gallicola
39	FALSE	Bacteria	Firmicutes	Bacilli	Lactobacillales	Lactobacillaceae	NA
38	FALSE	Bacteria	Proteobacteria	Alphaproteobacteria	Sphingomonadales	Sphingomonadaceae	NA
38	FALSE	Bacteria	Bacteroidetes	Bacteroidia	Sphingobacteriales	Sphingobacteriaceae	Sphingobacterium
37	FALSE	Bacteria	Firmicutes	Clostridia	Clostridiales	Family_XI	Murdochiella
37	FALSE	Bacteria	Firmicutes	Clostridia	Clostridiales	Ruminococcaceae	Ruminococcaceae_UCG-009
37	FALSE	Bacteria	Firmicutes	Clostridia	Clostridiales	Ruminococcaceae	Ruminococcaceae_UCG-011
37	FALSE	Bacteria	Actinobacteria	Actinobacteria	Bifidobacteriales	Bifidobacteriaceae	Scardovia
36	FALSE	Bacteria	Proteobacteria	Alphaproteobacteria	Sphingomonadales	Sphingomonadaceae	Sphingopyxis
35	FALSE	Bacteria	Proteobacteria	Gammaproteobacteria	Betaproteobacteriales	Burkholderiaceae	Achromobacter
35	FALSE	Bacteria	Firmicutes	Clostridia	Clostridiales	Peptostreptococcaceae	Clostridioides
35	FALSE	Bacteria	Actinobacteria	Actinobacteria	Micrococcales	Dermabacteraceae	Dermabacter
35	FALSE	Bacteria	Synergistetes	Synergistia	Synergistales	Synergistaceae	Jonquetella
34	FALSE	Bacteria	Bacteroidetes	Bacteroidia	Flavobacteriales	Weeksellaceae	Chryseobacterium
34	FALSE	Bacteria	Firmicutes	Clostridia	Clostridiales	Ruminococcaceae	Ruminococcaceae_UCG-008
33	FALSE	Bacteria	Tenericutes	Mollicutes	Anaeroplasmatales	Anaeroplasmataceae	Anaeroplasma
32	FALSE	Bacteria	Firmicutes	Clostridia	Clostridiales	Ruminococcaceae	Anaerotruncus
32	FALSE	Bacteria	Firmicutes	Clostridia	Clostridiales	Family_XI	Finegoldia
32	FALSE	Bacteria	Proteobacteria	Gammaproteobacteria	Enterobacteriales	Enterobacteriaceae	Hafnia-Obesumbacterium
31	FALSE	Bacteria	Firmicutes	Bacilli	Lactobacillales	Carnobacteriaceae	Carnobacterium
31	FALSE	Bacteria	Firmicutes	Clostridia	Clostridiales	Lachnospiraceae	Howardella

31	FALSE	Bacteria	Firmicutes	Clostridia	Clostridiales	Lachnospiraceae	Hungatella
30	FALSE	Bacteria	Actinobacteria	Coriobacteriia	Coriobacteriales	Coriobacteriales_Incertae_Sedis	Raoultibacter
30	FALSE	Bacteria	Firmicutes	Clostridia	Clostridiales	Ruminococcaceae	UBA1819
29	FALSE	Bacteria	Firmicutes	Clostridia	Clostridiales	Lachnospiraceae	Anaerosporobacter
29	FALSE	Bacteria	Proteobacteria	Gammaproteobacteria	Enterobacteriales	Enterobacteriaceae	Citrobacter
29	FALSE	Bacteria	Actinobacteria	Coriobacteriia	Coriobacteriales	Eggerthellaceae	Cryptobacterium
29	FALSE	Bacteria	Actinobacteria	Acidimicrobiia	Microtrichales	NA	NA
29	FALSE	Bacteria	Actinobacteria	Actinobacteria	Corynebacteriales	Nocardiaceae	Rhodococcus
29	FALSE	Bacteria	Proteobacteria	Alphaproteobacteria	Acetobacterales	Acetobacteraceae	Roseomonas
28	FALSE	Bacteria	Firmicutes	Clostridia	Clostridiales	Lachnospiraceae	28-4
28	FALSE	Bacteria	Firmicutes	Clostridia	Clostridiales	Lachnospiraceae	Acetatifactor
28	FALSE	Bacteria	Actinobacteria	Actinobacteria	Propionibacteriales	Propionibacteriaceae	Acidipropionibacterium
28	FALSE	Bacteria	Proteobacteria	Gammaproteobacteria	Betaproteobacteriales	Burkholderiaceae	Acidovorax
28	FALSE	Bacteria	Firmicutes	Bacilli	Bacillales	Paenibacillaceae	Ammoniphilus
28	FALSE	Bacteria	Proteobacteria	Alphaproteobacteria	Caulobacterales	Caulobacteraceae	Brevundimonas
28	FALSE	Bacteria	Firmicutes	Bacilli	Bacillales	Paenibacillaceae	Cohnella
28	FALSE	Bacteria	Firmicutes	Bacilli	Bacillales	Planococcaceae	Domibacillus
28	FALSE	Bacteria	Firmicutes	Bacilli	Lactobacillales	Aerococcaceae	Facklamia
28	FALSE	Bacteria	Proteobacteria	Gammaproteobacteria	Betaproteobacteriales	Burkholderiaceae	Kerstersia
28	FALSE	Bacteria	Firmicutes	Clostridia	Clostridiales	Lachnospiraceae	Lachnospiraceae_UCG-006
28	FALSE	Bacteria	Actinobacteria	Actinobacteria	Micromonosporales	Micromonosporaceae	Micromonospora
28	FALSE	Bacteria	Bacteroidetes	Bacteroidia	Bacteroidales	Muribaculaceae	Muribaculum
28	FALSE	Bacteria	Actinobacteria	Coriobacteriia	Coriobacteriales	NA	NA
28	FALSE	Bacteria	Proteobacteria	Alphaproteobacteria	Acetobacterales	Acetobacteraceae	NA
28	FALSE	Bacteria	Actinobacteria	Coriobacteriia	Coriobacteriales	Atopobiaceae	NA
28	FALSE	Bacteria	Firmicutes	Bacilli	NA	NA	NA
28	FALSE	Bacteria	Verrucomicrobia	Verrucomicrobiae	Opitutales	NA	NA
28	FALSE	Bacteria	Cyanobacteria	Oxyphotobacteria	Phormidesmiales	Nodosilineaceae	NA
28	FALSE	Bacteria	Firmicutes	Negativicutes	Selenomonadales	NA	NA
28	FALSE	Bacteria	Actinobacteria	Actinobacteria	Corynebacteriales	NA	NA
28	FALSE	Bacteria	Acidobacteria	FFCH5909	NA	NA	NA
28	FALSE	Bacteria	Actinobacteria	Actinobacteria	Propionibacteriales	Nocardioidaceae	Nocardioides
28	FALSE	Bacteria	Firmicutes	Clostridia	Clostridiales	Peptostreptococcaceae	Paraclostridium
28	FALSE	Bacteria	Proteobacteria	Alphaproteobacteria	Rhizobiales	Beijerinckiaceae	Psychroglaciecola
28	FALSE	Bacteria	Proteobacteria	Gammaproteobacteria	Xanthomonadales	Xanthomonadaceae	SN8

28	FALSE	Bacteria	Firmicutes	Bacilli	Bacillales	Bacillaceae	Terribacillus	
28	FALSE	Bacteria	Actinobacteria	Actinobacteria	Actinomycetales	Actinomycetaceae	Trueperella	
27	FALSE	Bacteria	Proteobacteria	Gammaproteobacteria	Aeromonadales	Aeromonadaceae	Aeromonas	
27	FALSE	Bacteria	Proteobacteria	Gammaproteobacteria	Oceanospirillales	Alcanivoracaceae	Alcanivorax	
27	FALSE	Bacteria	Firmicutes	Erysipelotrichia	Erysipelotrichales	Erysipelotrichaceae	Allobaculum	
27	FALSE	Bacteria	Firmicutes	Bacilli	Lactobacillales	Carnobacteriaceae	Alloiococcus	
27	FALSE	Bacteria	Proteobacteria	Alphaproteobacteria	Rhizobiales	Rhizobiaceae	Aminobacter	
27	FALSE	Bacteria	Proteobacteria	Gammaproteobacteria	Aeromonadales	Succinivibrionaceae	Anaerobiospirillum	
27	FALSE	Bacteria	Firmicutes	Clostridia	Clostridiales	Lachnospiraceae	Anaerocolumna	
27	FALSE	Bacteria	Actinobacteria	Actinobacteria	Actinomycetales	Actinomycetaceae	Arcanobacterium	
27	FALSE	Bacteria	Proteobacteria	Gammaproteobacteria	Enterobacteriales	Enterobacteriaceae	ATCC-39006	
27	FALSE	Bacteria	Proteobacteria	Alphaproteobacteria	Rhizobiales	Rhizobiaceae	Aureimonas	
27	FALSE	Bacteria	Proteobacteria	Alphaproteobacteria	Sphingomonadales	Sphingomonadaceae	Blastomonas	
27	FALSE	Bacteria	Spirochaetes	Brachyspirae	Brachyspirales	Brachyspiraceae	Brachyspira	
27	FALSE	Bacteria	Firmicutes	Clostridia	Clostridiales	Caldicoprobacteraceae	Caldicoprobacter	
27	FALSE	Bacteria	Proteobacteria	Alphaproteobacteria	Caedibacterales	Caedibacteraceae	Candidatus_Nucleicultrix	
27	FALSE	Bacteria	Firmicutes	Negativicutes	Selenomonadales	Veillonellaceae	Centipeda	
27	FALSE	Bacteria	Firmicutes	Clostridia	Clostridiales	Christensenellaceae	Christensenella	
27	FALSE	Bacteria	Cyanobacteria	Oxyphotobacteria	Nostocales	Chroococcidiopsaceae	Chroococcidiopsis_SAG_2023	
27	FALSE	Bacteria	Bacteroidetes	Bacteroidia	Flavobacteriales	Weeksellaceae	Cloacibacterium	
27	FALSE	Bacteria	Proteobacteria	Gammaproteobacteria	Enterobacteriales	Enterobacteriaceae	Cosenzaea	
27	FALSE	Bacteria	Proteobacteria	Gammaproteobacteria	Betaproteobacteriales	Burkholderiaceae	Cupriavidus	
27	FALSE	Bacteria	Deinococcus-Thermus	Deinococci	Deinococcales	Deinococcaceae	Deinococcus	
27	FALSE	Bacteria	Actinobacteria	Coriobacteriia	Coriobacteriales	Eggerthellaceae	Denitrobacterium	
27	FALSE	Bacteria	Firmicutes	Clostridia	Clostridiales	Peptococcaceae	Desulfitibacter	
27	FALSE	Bacteria	Proteobacteria	Deltaproteobacteria	Desulfobacterales	Desulfobulbaceae	Desulfobulbus	
27	FALSE	Bacteria	Proteobacteria	Alphaproteobacteria	Rhizobiales	Devosiaceae	Devosia	
27	FALSE	Bacteria	Proteobacteria	Gammaproteobacteria	Xanthomonadales	Rhodanobacteraceae	Dokdonella	
27	FALSE	Bacteria	Bacteroidetes	Bacteroidia	Cytophagales	Spirosomaceae	Dyadobacter	
27	FALSE	Bacteria	Proteobacteria	Gammaproteobacteria	Pseudomonadales	Moraxellaceae	Enhydrobacter	
27	FALSE	Bacteria	Firmicutes	Erysipelotrichia	Erysipelotrichales	Erysipelotrichaceae	Faecalicoccus	
27	FALSE	Bacteria	Firmicutes	Bacilli	Bacillales	Bacillaceae	Fictibacillus	
27	FALSE	Bacteria	Chloroflexi	Anaerolineae	Anaerolineales	Anaerolineaceae	Flexilinea	
27	FALSE	Bacteria	Synergistetes	Synergistia	Synergistales	Synergistaceae	Fretibacterium	

27	FALSE	Bacteria	Firmicutes	Bacilli	Lactobacillales	Aerococcaceae	Globicatella
27	FALSE	Bacteria	Actinobacteria	Actinobacteria	Micrococcales	Micrococcaceae	Glutamicibacter
27	FALSE	Bacteria	Proteobacteria	Alphaproteobacteria	Rhodobacterales	Rhodobacteraceae	Haematobacter
27	FALSE	Bacteria	Proteobacteria	Gammaproteobacteria	Oceanospirillales	Halomonadaceae	Halomonas
27	FALSE	Bacteria	Firmicutes	Clostridia	Clostridiales	Family_XI	Helcococcus
27	FALSE	Bacteria	Proteobacteria	Gammaproteobacteria	Betaproteobacteriales	Burkholderiaceae	Hydrogenophaga
27	FALSE	Bacteria	Proteobacteria	Alphaproteobacteria	Rhizobiales	Hyphomicrobiaceae	Hyphomicrobium
27	FALSE	Bacteria	Firmicutes	Bacilli	Lactobacillales	Aerococcaceae	Ignavigranum
27	FALSE	Bacteria	Actinobacteria	Actinobacteria	Micrococcales	Intrasporangiaceae	Janibacter
27	FALSE	Bacteria	Firmicutes	Clostridia	Clostridiales	Lachnospiraceae	Johnsonella
27	FALSE	Bacteria	Firmicutes	Clostridia	Clostridiales	Lachnospiraceae	Lachnoanaerobaculum
27	FALSE	Bacteria	Bacteroidetes	Bacteroidia	Chitinophagales	Chitinophagaceae	Lacibacter
27	FALSE	Bacteria	Actinobacteria	Actinobacteria	Micrococcales	Microbacteriaceae	Leucobacter
27	FALSE	Bacteria	Proteobacteria	Gammaproteobacteria	Betaproteobacteriales	Burkholderiaceae	Massilia
27	FALSE	Bacteria	Firmicutes	Erysipelotrichia	Erysipelotrichales	Erysipelotrichaceae	Merdibacter
27	FALSE	Bacteria	Actinobacteria	Actinobacteria	Propionibacteriales	Propionibacteriaceae	Micropruina
27	FALSE	Bacteria	Proteobacteria	Gammaproteobacteria	Pseudomonadales	Moraxellaceae	Moraxella
27	FALSE	Bacteria	Actinobacteria	Actinobacteria	Corynebacteriales	Mycobacteriaceae	Mycobacterium
27	FALSE	Bacteria	Firmicutes	Clostridia	Clostridiales	Family_XI	NA
27	FALSE	Bacteria	Firmicutes	Bacilli	Bacillales	Paenibacillaceae	NA
27	FALSE	Bacteria	Proteobacteria	Gammaproteobacteria	Betaproteobacteriales	NA	NA
27	FALSE	Bacteria	Actinobacteria	Coriobacteriia	Coriobacteriales	Coriobacteriaceae	NA
27	FALSE	Bacteria	Firmicutes	Clostridia	Clostridiales	Syntrophomonadaceae	NA
27	FALSE	Bacteria	Chloroflexi	Chloroflexia	Thermomicrobiales	JG30-KF-CM45	NA
27	FALSE	Bacteria	Actinobacteria	Actinobacteria	Propionibacteriales	Propionibacteriaceae	NA
27	FALSE	Bacteria	Verrucomicrobia	Verrucomicrobiae	NA	NA	NA
27	FALSE	Bacteria	Actinobacteria	Actinobacteria	Micromonosporales	Micromonosporaceae	NA
27	FALSE	Bacteria	Actinobacteria	Actinobacteria	Streptomycetales	Streptomycetaceae	NA
27	FALSE	Bacteria	Firmicutes	Bacilli	Lactobacillales	Streptococcaceae	NA
27	FALSE	Bacteria	Proteobacteria	Alphaproteobacteria	NA	NA	NA
27	FALSE	Archaea	Euryarchaeota	Methanobacteria	Methanobacteriales	Methanobacteriaceae	NA
27	FALSE	Bacteria	Actinobacteria	Actinobacteria	NA	NA	NA
27	FALSE	Bacteria	Proteobacteria	Alphaproteobacteria	Micavibrionales	NA	NA
27	FALSE	Bacteria	Actinobacteria	Actinobacteria	Micrococcales	NA	NA
27	FALSE	Bacteria	Chloroflexi	Chloroflexia	Kallotenuales	NA	NA

27	FALSE	Bacteria	Tenericutes	Mollicutes	NA	NA	NA
27	FALSE	Bacteria	Proteobacteria	Gammaproteobacteria	Betaproteobacteriales	Rhodocyclaceae	NA
27	FALSE	Bacteria	Proteobacteria	Gammaproteobacteria	Betaproteobacteriales	Neisseriaceae	NA
27	FALSE	Eukaryota	NA	NA	NA	NA	NA
27	FALSE	Bacteria	Verrucomicrobia	Verrucomicrobiae	Verrucomicrobiales	NA	NA
27	FALSE	Bacteria	Actinobacteria	Actinobacteria	Micrococcales	Micrococcaceae	NA
27	FALSE	Bacteria	Bacteroidetes	Bacteroidia	Flavobacteriales	Weeksellaceae	NA
27	FALSE	Bacteria	Proteobacteria	Gammaproteobacteria	Betaproteobacteriales	Neisseriaceae	Neisseria
27	FALSE	Bacteria	Actinobacteria	Actinobacteria	Streptosporangiales	Nocardiopsaceae	Nocardiopsis
27	FALSE	Bacteria	Proteobacteria	Alphaproteobacteria	Sphingomonadales	Sphingomonadaceae	Novosphingobium
27	FALSE	Bacteria	Bacteroidetes	Bacteroidia	Sphingobacteriales	Sphingobacteriaceae	Nubsella
27	FALSE	Bacteria	Bacteroidetes	Bacteroidia	Sphingobacteriales	Sphingobacteriaceae	Pedobacter
27	FALSE	Bacteria	Proteobacteria	Alphaproteobacteria	Caulobacterales	Caulobacteraceae	Phenylobacterium
27	FALSE	Bacteria	Proteobacteria	Alphaproteobacteria	Rhizobiales	Rhizobiaceae	Phyllobacterium
27	FALSE	Bacteria	Proteobacteria	Gammaproteobacteria	Betaproteobacteriales	Burkholderiaceae	Pigmentiphaga
27	FALSE	Bacteria	Planctomycetes	Planctomycetacia	Pirellulales	Pirellulaceae	Pirellula
27	FALSE	Bacteria	Firmicutes	Clostridia	Clostridiales	Eubacteriaceae	Pseudoramibacter
27	FALSE	Bacteria	Firmicutes	Bacilli	Bacillales	Planococcaceae	Psychrobacillus
27	FALSE	Bacteria	Proteobacteria	Gammaproteobacteria	Betaproteobacteriales	Burkholderiaceae	Pusillimonas
27	FALSE	Bacteria	Firmicutes	Clostridia	Clostridiales	Ruminococcaceae	Pygmaiobacter
27	FALSE	Bacteria	Proteobacteria	Gammaproteobacteria	Betaproteobacteriales	Burkholderiaceae	Ralstonia
27	FALSE	Bacteria	Proteobacteria	Alphaproteobacteria	Reyranellales	Reyranellaceae	Reyranella
27	FALSE	Bacteria	Firmicutes	Bacilli	Bacillales	Planococcaceae	Rummeliibacillus
27	FALSE	Bacteria	Firmicutes	Negativicutes	Selenomonadales	Veillonellaceae	Selenomonas
27	FALSE	Bacteria	Firmicutes	Bacilli	Bacillales	Planococcaceae	Solibacillus
27	FALSE	Bacteria	Proteobacteria	Alphaproteobacteria	Sphingomonadales	Sphingomonadaceae	Sphingomonas
27	FALSE	Bacteria	Bacteroidetes	Bacteroidia	Cytophagales	Spirosomaceae	Spirosoma
27	FALSE	Bacteria	Firmicutes	Clostridia	Clostridiales	Lachnospiraceae	Stomatobaculum
27	FALSE	Bacteria	Proteobacteria	Gammaproteobacteria	Aeromonadales	Succinivibrionaceae	Succinivibrio
27	FALSE	Bacteria	Firmicutes	Bacilli	Lactobacillales	Enterococcaceae	Tetragenococcus
27	FALSE	Bacteria	Firmicutes	Clostridia	Clostridiales	Family_XI	Tissierella
27	FALSE	Bacteria	Spirochaetes	Spirochaetia	Spirochaetales	Spirochaetaceae	Treponema_2
27	FALSE	Bacteria	Tenericutes	Mollicutes	Mycoplasmatales	Mycoplasmataceae	Ureaplasma
27	FALSE	Bacteria	Proteobacteria	Gammaproteobacteria	Cardiobacteriales	Wohlfahrtiimonadaceae	Wohlfahrtiimonas
27	FALSE	Bacteria	Proteobacteria	Alphaproteobacteria	Rhizobiales	Xanthobacteraceae	Xanthobacter

27	FALSE	Bacteria	Firmicutes	Clostridia	Clostridiales	Lachnospiraceae	XBB1006
27	FALSE	Bacteria	Proteobacteria	Gammaproteobacteria	Betaproteobacteriales	Rhodocyclaceae	Zoogloea
26	FALSE	Bacteria	Firmicutes	Bacilli	Lactobacillales	Aerococcaceae	Abiotrophia
26	FALSE	Bacteria	Proteobacteria	Gammaproteobacteria	Gammaproteobacteria_Incertae_Sedis	Unknown_Family	Acidibacter
26	FALSE	Bacteria	Actinobacteria	Actinobacteria	Streptosporangiales	Thermomonosporaceae	Actinomadura
26	FALSE	Bacteria	Actinobacteria	Actinobacteria	Actinomycetales	Actinomycetaceae	Actinomyces
26	FALSE	Bacteria	Actinobacteria	Actinobacteria	Propionibacteriales	Nocardioidaceae	Aeromicrobium
26	FALSE	Bacteria	Proteobacteria	Gammaproteobacteria	Pasteurellales	Pasteurellaceae	Aggregatibacter
26	FALSE	Bacteria	Proteobacteria	Gammaproteobacteria	Betaproteobacteriales	Burkholderiaceae	Alcaligenes
26	FALSE	Bacteria	Firmicutes	Bacilli	Lactobacillales	Carnobacteriaceae	Allofustis
26	FALSE	Bacteria	Proteobacteria	Alphaproteobacteria	Sphingomonadales	Sphingomonadaceae	Altererythrobacter
26	FALSE	Bacteria	Firmicutes	Bacilli	Bacillales	Bacillaceae	Anaerobacillus
26	FALSE	Bacteria	Firmicutes	Bacilli	Bacillales	Paenibacillaceae	Aneurinibacillus
26	FALSE	Bacteria	Epsilonbacteraeota	Campylobacteria	Campylobacterales	Arcobacteraceae	Arcobacter
26	FALSE	Bacteria	Actinobacteria	Coriobacteriia	Coriobacteriales	Atopobiaceae	Atopobium
26	FALSE	Bacteria	Bacteroidetes	Bacteroidia	Flavobacteriales	Weeksellaceae	Bergeyella
26	FALSE	Bacteria	Proteobacteria	Alphaproteobacteria	Rhizobiales	Xanthobacteraceae	Bradyrhizobium
26	FALSE	Bacteria	Bacteroidetes	Bacteroidia	Cytophagales	Amoebophilaceae	Candidatus_Amoebophilus
26	FALSE	Bacteria	Bacteroidetes	Bacteroidia	Flavobacteriales	Flavobacteriaceae	Capnocytophaga
26	FALSE	Bacteria	Proteobacteria	Gammaproteobacteria	Cardiobacteriales	Cardiobacteriaceae	Cardiobacterium
26	FALSE	Bacteria	Firmicutes	Erysipelotrichia	Erysipelotrichales	Erysipelotrichaceae	Catenisphaera
26	FALSE	Bacteria	Actinobacteria	Actinobacteria	Micrococcales	Cellulomonadaceae	Cellulomonas
26	FALSE	Bacteria	Firmicutes	Clostridia	Clostridiales	Lachnospiraceae	Cellulosilyticum
26	FALSE	Bacteria	Fusobacteria	Fusobacteriia	Fusobacteriales	Fusobacteriaceae	Cetobacterium
26	FALSE	Bacteria	Actinobacteria	Coriobacteriia	Coriobacteriales	Eggerthellaceae	CHKCI002
26	FALSE	Bacteria	Firmicutes	Clostridia	Clostridiales	Clostridiaceae_1	Clostridium_sensu_stricto_11
26	FALSE	Bacteria	Firmicutes	Clostridia	Clostridiales	Clostridiaceae_1	Clostridium_sensu_stricto_7
26	FALSE	Bacteria	Actinobacteria	Coriobacteriia	Coriobacteriales	Atopobiaceae	Coriobacteriaceae_UCG-003
26	FALSE	Bacteria	Actinobacteria	Actinobacteria	Corynebacteriales	Dietziaceae	Dietzia
26	FALSE	Bacteria	Proteobacteria	Gammaproteobacteria	Betaproteobacteriales	Neisseriaceae	Eikenella
26	FALSE	Bacteria	Proteobacteria	Alphaproteobacteria	Rhizobiales	Rhizobiaceae	Ensifer
26	FALSE	Bacteria	Actinobacteria	Actinobacteria	Actinomycetales	Actinomycetaceae	F0332
26	FALSE	Bacteria	Bacteroidetes	Bacteroidia	Cytophagales	Spirosomaceae	Flectobacillus
26	FALSE	Bacteria	Firmicutes	Bacilli	Bacillales	Bacillaceae	Geobacillus
26	FALSE	Bacteria	Firmicutes	Bacilli	Bacillales	Bacillaceae	Gracilibacillus

26	FALSE	Bacteria	Firmicutes	Clostridia	Clostridiales	Clostridiaceae_1	Hathewaya
26	FALSE	Bacteria	Firmicutes	Bacilli	Bacillales	Staphylococcaceae	Jeotgalicoccus
26	FALSE	Bacteria	Firmicutes	Bacilli	Bacillales	Thermoactinomycetaceae	Kroppenstedtia
26	FALSE	Bacteria	Verrucomicrobia	Verrucomicrobiae	Chthoniobacterales	Chthoniobacteraceae	LD29
26	FALSE	Bacteria	Actinobacteria	Actinobacteria	Micrococcales	Microbacteriaceae	Leifsonia
26	FALSE	Bacteria	Fusobacteria	Fusobacteriia	Fusobacteriales	Leptotrichiaceae	Leptotrichia
26	FALSE	Bacteria	Actinobacteria	Coriobacteriia	Coriobacteriales	Atopobiaceae	Libanicoccus
26	FALSE	Bacteria	Firmicutes	Bacilli	Bacillales	Staphylococcaceae	Macrococcus
26	FALSE	Bacteria	Proteobacteria	Alphaproteobacteria	Rhizobiales	Rhizobiaceae	Mesorhizobium
26	FALSE	Bacteria	Tenericutes	Mollicutes	Mycoplasmatales	Mycoplasmataceae	Mycoplasma
26	FALSE	NA	NA	NA	NA	NA	NA
26	FALSE	Bacteria	Proteobacteria	Gammaproteobacteria	Betaproteobacteriales	Burkholderiaceae	NA
26	FALSE	Bacteria	Firmicutes	Bacilli	Lactobacillales	Enterococcaceae	NA
26	FALSE	Bacteria	Actinobacteria	Actinobacteria	Corynebacteriales	Nocardiaceae	NA
26	FALSE	Bacteria	Proteobacteria	Alphaproteobacteria	Rickettsiales	Mitochondria	NA
26	FALSE	Bacteria	Actinobacteria	Actinobacteria	Micrococcales	Microbacteriaceae	NA
26	FALSE	Bacteria	Actinobacteria	Actinobacteria	Bifidobacteriales	Bifidobacteriaceae	NA
26	FALSE	Bacteria	Bacteroidetes	Bacteroidia	Bacteroidales	Dysgonomonadaceae	NA
26	FALSE	Bacteria	Actinobacteria	NA	NA	NA	NA
26	FALSE	Bacteria	Verrucomicrobia	Verrucomicrobiae	Opitutales	Puniceicoccaceae	NA
26	FALSE	Bacteria	Proteobacteria	Alphaproteobacteria	Rhizobiales	Xanthobacteraceae	NA
26	FALSE	Bacteria	Firmicutes	Bacilli	Bacillales	Staphylococcaceae	NA
26	FALSE	Bacteria	Spirochaetes	Spirochaetia	Spirochaetales	Spirochaetaceae	NA
26	FALSE	Bacteria	Firmicutes	Bacilli	Lactobacillales	Carnobacteriaceae	NA
26	FALSE	Bacteria	Bacteroidetes	Bacteroidia	Sphingobacteriales	Sphingobacteriaceae	NA
26	FALSE	Bacteria	Proteobacteria	Gammaproteobacteria	NA	NA	NA
26	FALSE	Bacteria	Kiritimatiellaeota	Kiritimatiellae	WCHB1-41	NA	NA
26	FALSE	Bacteria	Synergistetes	Synergistia	Synergistales	Synergistaceae	NA
26	FALSE	Bacteria	Bacteroidetes	Bacteroidia	Bacteroidales	Tannerellaceae	NA
26	FALSE	Bacteria	Proteobacteria	Alphaproteobacteria	Rhizobiales	Rhizobiaceae	Neorhizobium
26	FALSE	Bacteria	Firmicutes	Clostridia	Clostridiales	Ruminococcaceae	Papillibacter
26	FALSE	Bacteria	Actinobacteria	Coriobacteriia	Coriobacteriales	Eggerthellaceae	Paraeggerthella
26	FALSE	Bacteria	Firmicutes	Bacilli	Bacillales	Bacillaceae	Paucisalibacillus
26	FALSE	Bacteria	Firmicutes	Negativicutes	Selenomonadales	Veillonellaceae	Pectinatus
26	FALSE	Bacteria	Proteobacteria	Gammaproteobacteria	Betaproteobacteriales	Burkholderiaceae	Pelomonas

26	FALSE	Bacteria	Proteobacteria	Gammaproteobacteria	Enterobacteriales	Enterobacteriaceae	Plesiomonas
26	FALSE	Bacteria	Proteobacteria	Gammaproteobacteria	Enterobacteriales	Enterobacteriaceae	Pluralibacter
26	FALSE	Bacteria	Bacteroidetes	Bacteroidia	Bacteroidales	Prevotellaceae	Prevotellaceae_Ga6A1_group
26	FALSE	Bacteria	Actinobacteria	Actinobacteria	Propionibacteriales	Propionibacteriaceae	Propioniferax
26	FALSE	Bacteria	Proteobacteria	Alphaproteobacteria	Rhizobiales	Rhizobiaceae	Pseudochrobactrum
26	FALSE	Bacteria	Firmicutes	Clostridia	Clostridiales	Ruminococcaceae	Pseudoflavonifractor
26	FALSE	Bacteria	Actinobacteria	Actinobacteria	Propionibacteriales	Propionibacteriaceae	Pseudopropionibacterium
26	FALSE	Bacteria	Proteobacteria	Gammaproteobacteria	Betaproteobacteriales	Burkholderiaceae	Pseudorhodoferax
26	FALSE	Bacteria	Firmicutes	Clostridia	Clostridiales	Lachnospiraceae	Robinsoniella
26	FALSE	Bacteria	Firmicutes	Negativicutes	Selenomonadales	Veillonellaceae	Selenomonas_4
26	FALSE	Bacteria	Proteobacteria	Alphaproteobacteria	Rhizobiales	Rhizobiaceae	Shinella
26	FALSE	Bacteria	Proteobacteria	Alphaproteobacteria	Azospirillales	Azospirillaceae	Skermanella
26	FALSE	Bacteria	Spirochaetes	Spirochaetia	Spirochaetales	Spirochaetaceae	Sphaerochaeta
26	FALSE	Bacteria	Proteobacteria	Alphaproteobacteria	Sphingomonadales	Sphingomonadaceae	Sphingobium
26	FALSE	Bacteria	Firmicutes	Clostridia	Clostridiales	Lachnospiraceae	Sporobacterium
26	FALSE	Bacteria	Firmicutes	Bacilli	Bacillales	Planococcaceae	Sporosarcina
26	FALSE	Bacteria	Proteobacteria	Gammaproteobacteria	Aeromonadales	Succinivibrionaceae	Succinatimonas
26	FALSE	Bacteria	Firmicutes	Clostridia	Clostridiales	Syntrophomonadaceae	Syntrophomonas
26	FALSE	Bacteria	Proteobacteria	Gammaproteobacteria	Betaproteobacteriales	Burkholderiaceae	Tepidimonas
26	FALSE	Bacteria	Deinococcus-Thermus	Deinococci	Thermales	Thermaceae	Thermus
26	FALSE	Bacteria	Firmicutes	Bacilli	Lactobacillales	Enterococcaceae	Vagococcus
26	FALSE	Bacteria	Proteobacteria	Gammaproteobacteria	Betaproteobacteriales	Burkholderiaceae	Variovorax
26	FALSE	Bacteria	Firmicutes	Bacilli	Bacillales	Bacillaceae	Virgibacillus
26	FALSE	Bacteria	Firmicutes	Clostridia	Clostridiales	Family_XI	W5053
26	FALSE	Bacteria	Proteobacteria	Gammaproteobacteria	Betaproteobacteriales	Burkholderiaceae	Xylophilus
26	FALSE	Bacteria	Proteobacteria	Gammaproteobacteria	Enterobacteriales	Enterobacteriaceae	Yersinia
25	FALSE	Bacteria	Actinobacteria	Actinobacteria	Actinomycetales	Actinomycetaceae	Actinotignum
25	FALSE	Bacteria	Proteobacteria	Alphaproteobacteria	Rhizobiales	Rhizobiaceae	Allorhizobium-Neorhizobium-Pararhizobium-Rhizobium
25	FALSE	Bacteria	Firmicutes	Clostridia	Clostridiales	Family_XIII	Anaerovorax
25	FALSE	Archaea	Euryarchaeota	Thermoplasmata	Methanomassiliicoccales	Methanomethylophilaceae	Candidatus_Methanomethylophilus
25	FALSE	Bacteria	Patescibacteria	Saccharimonadia	Saccharimonadales	Saccharimonadaceae	Candidatus_Saccharimonas
25	FALSE	Bacteria	Firmicutes	Clostridia	Clostridiales	Clostridiaceae_1	Clostridium_sensu_stricto_2
25	FALSE	Bacteria	Firmicutes	Bacilli	Lactobacillales	Aerococcaceae	Eremococcus
25	FALSE	Bacteria	Firmicutes	Erysipelotrichia	Erysipelotrichales	Erysipelotrichaceae	Erysipelotrichaceae_UCG-004

25	FALSE	Bacteria	Firmicutes	Erysipelotrichia	Erysipelotrichales	Erysipelotrichaceae	Erysipelotrichaceae_UCG-006
25	FALSE	Bacteria	Bacteroidetes	Bacteroidia	Flavobacteriales	Flavobacteriaceae	Flavobacterium
25	FALSE	Bacteria	Actinobacteria	Actinobacteria	Corynebacteriales	Nocardiaceae	Gordonia
25	FALSE	Bacteria	Proteobacteria	Gammaproteobacteria	Betaproteobacteriales	Burkholderiaceae	Herbaspirillum
25	FALSE	Bacteria	Firmicutes	Clostridia	Clostridiales	Lachnospiraceae	Lachnoclostridium_10
25	FALSE	Bacteria	Proteobacteria	Gammaproteobacteria	Betaproteobacteriales	Burkholderiaceae	Lautropia
25	FALSE	Bacteria	Proteobacteria	Deltaproteobacteria	Desulfovibrionales	Desulfovibrionaceae	Mailhella
25	FALSE	Bacteria	Firmicutes	Negativicutes	Selenomonadales	Veillonellaceae	Mitsuokella
25	FALSE	Bacteria	Bacteroidetes	Bacteroidia	Bacteroidales	Rikenellaceae	NA
25	FALSE	Bacteria	Proteobacteria	NA	NA	NA	NA
25	FALSE	Bacteria	Lentisphaerae	Lentisphaeria	Victivallales	NA	NA
25	FALSE	Bacteria	Firmicutes	Bacilli	Lactobacillales	NA	NA
25	FALSE	Bacteria	Bacteroidetes	NA	NA	NA	NA
25	FALSE	Bacteria	Actinobacteria	Actinobacteria	Actinomycetales	Actinomycetaceae	NA
25	FALSE	Bacteria	Firmicutes	Negativicutes	Selenomonadales	Acidaminococcaceae	NA
25	FALSE	Bacteria	Firmicutes	Bacilli	Bacillales	Staphylococcaceae	Nosocomiicoccus
25	FALSE	Bacteria	Proteobacteria	Alphaproteobacteria	Rhizobiales	Rhizobiaceae	Ochrobactrum
25	FALSE	Bacteria	Firmicutes	Clostridia	Clostridiales	Lachnospiraceae	Oribacterium
25	FALSE	Bacteria	Firmicutes	Bacilli	Bacillales	Bacillaceae	Ornithinibacillus
25	FALSE	Bacteria	Proteobacteria	Alphaproteobacteria	Rhodobacterales	Rhodobacteraceae	Paracoccus
25	FALSE	Bacteria	Actinobacteria	Actinobacteria	Propionibacteriales	Propionibacteriaceae	Propionimicrobium
25	FALSE	Bacteria	Firmicutes	Clostridia	Clostridiales	Ruminococcaceae	Ruminococcaceae_V9D2013_group
25	FALSE	Bacteria	Firmicutes	Clostridia	Clostridiales	Clostridiaceae_1	Sarcina
25	FALSE	Bacteria	Proteobacteria	Gammaproteobacteria	Enterobacteriales	Enterobacteriaceae	Shimwellia
25	FALSE	Bacteria	Fusobacteria	Fusobacteriia	Fusobacteriales	Leptotrichiaceae	Sneathia
25	FALSE	Bacteria	Synergistetes	Synergistia	Synergistales	Synergistaceae	Synergistes
24	FALSE	Bacteria	Actinobacteria	Actinobacteria	Actinomycetales	Actinomycetaceae	Actinobaculum
24	FALSE	Bacteria	Firmicutes	Bacilli	Lactobacillales	Aerococcaceae	Aerococcus
24	FALSE	Bacteria	Bacteroidetes	Bacteroidia	Bacteroidales	Prevotellaceae	Alloprevotella
24	FALSE	Bacteria	Actinobacteria	Actinobacteria	Bifidobacteriales	Bifidobacteriaceae	Alloscardovia
24	FALSE	Bacteria	Firmicutes	Clostridia	Clostridiales	Eubacteriaceae	Anaerofustis
24	FALSE	Bacteria	Firmicutes	Negativicutes	Selenomonadales	Veillonellaceae	Anaeroglobus
24	FALSE	Bacteria	Bacteroidetes	Bacteroidia	Bacteroidales	Muribaculaceae	CAG-873
24	FALSE	Bacteria	Actinobacteria	Actinobacteria	Micrococcales	Promicromonosporaceae	Cellulosimicrobium
24	FALSE	Bacteria	Verrucomicrobia	Verrucomicrobiae	Opitutales	Puniceicoccaceae	Cerasicoccus

24	FALSE	Bacteria	Firmicutes	Clostridia	Clostridiales	Clostridiaceae_1	Clostridium_sensu_stricto_3
24	FALSE	Bacteria	Proteobacteria	Gammaproteobacteria	Betaproteobacteriales	Burkholderiaceae	Comamonas
24	FALSE	Bacteria	Actinobacteria	Coriobacteriia	Coriobacteriales	Eggerthellaceae	DNF00809
24	FALSE	Bacteria	Actinobacteria	Coriobacteriia	Coriobacteriales	Coriobacteriaceae	Enorma
24	FALSE	Bacteria	Firmicutes	Clostridia	Clostridiales	Lachnospiraceae	Herbinix
24	FALSE	Bacteria	Proteobacteria	Gammaproteobacteria	Cardiobacteriales	Wohlfahrtiimonadaceae	Ignatzschineria
24	FALSE	Bacteria	Proteobacteria	Gammaproteobacteria	Enterobacteriales	Enterobacteriaceae	Kosakonia
24	FALSE	Bacteria	Actinobacteria	Actinobacteria	Micrococcales	Microbacteriaceae	Microbacterium
24	FALSE	Bacteria	Firmicutes	Clostridia	Clostridiales	Family_XIII	Mogibacterium
24	FALSE	Bacteria	Actinobacteria	Coriobacteriia	Coriobacteriales	Eggerthellaceae	NA
24	FALSE	Bacteria	Lentisphaerae	Lentisphaeria	Victivallales	Victivallaceae	NA
24	FALSE	Bacteria	Firmicutes	Bacilli	Lactobacillales	Aerococcaceae	NA
24	FALSE	Bacteria	Proteobacteria	Gammaproteobacteria	Aeromonadales	Succinivibrionaceae	NA
24	FALSE	Bacteria	Proteobacteria	Alphaproteobacteria	Rhodobacterales	Rhodobacteraceae	NA
24	FALSE	Bacteria	Firmicutes	Clostridia	Clostridiales	Peptostreptococcaceae	Paeniclostridium
24	FALSE	Bacteria	Bacteroidetes	Bacteroidia	Bacteroidales	Prevotellaceae	Prevotellaceae_UCG-001
24	FALSE	Bacteria	Actinobacteria	Actinobacteria	Micrococcales	Micrococcaceae	Pseudoglutamicibacter
24	FALSE	Bacteria	Bacteroidetes	Bacteroidia	Bacteroidales	Rikenellaceae	Rikenella
24	FALSE	Bacteria	Firmicutes	Clostridia	Clostridiales	Ruminococcaceae	Ruminococcaceae_UCG-007
24	FALSE	Bacteria	Firmicutes	Clostridia	Clostridiales	Family_XI	Sedimentibacter
23	FALSE	Bacteria	Firmicutes	Negativicutes	Selenomonadales	Veillonellaceae	Allisonella
23	FALSE	Bacteria	Bacteroidetes	Bacteroidia	Bacteroidales	Dysgonomonadaceae	Dysgonomonas
23	FALSE	Archaea	Euryarchaeota	Thermoplasmata	Methanomassiliicoccales	Methanomassiliicoccaceae	Methanomassiliicoccus
23	FALSE	Bacteria	Firmicutes	Clostridia	Clostridiales	Clostridiaceae_1	NA
23	FALSE	Bacteria	Firmicutes	Clostridia	Clostridiales	Peptostreptococcaceae	Peptostreptococcus
23	FALSE	Bacteria	Bacteroidetes	Bacteroidia	Bacteroidales	Rikenellaceae	Rikenellaceae_RC9_gut_group
23	FALSE	Bacteria	Firmicutes	Bacilli	Lactobacillales	Streptococcaceae	Streptococcus
22	FALSE	Bacteria	Firmicutes	Erysipelotrichia	Erysipelotrichales	Erysipelotrichaceae	Dielma
22	FALSE	Bacteria	Fusobacteria	Fusobacteriia	Fusobacteriales	Fusobacteriaceae	Fusobacterium
22	FALSE	Bacteria	Firmicutes	Erysipelotrichia	Erysipelotrichales	Erysipelotrichaceae	Holdemanella
22	FALSE	Bacteria	Firmicutes	Clostridia	Clostridiales	Ruminococcaceae	NA
22	FALSE	Bacteria	Proteobacteria	Gammaproteobacteria	Pseudomonadales	Pseudomonadaceae	NA
22	FALSE	Bacteria	NA	NA	NA	NA	NA
22	FALSE	Bacteria	Lentisphaerae	Lentisphaeria	Victivallales	vadinBE97	NA
22	FALSE	Bacteria	Bacteroidetes	Bacteroidia	Bacteroidales	Marinifilaceae	Sanguibacteroides

22	FALSE	Bacteria	Firmicutes	Clostridia	Clostridiales	Lachnospiraceae	Tyzzerella_4
21	FALSE	Bacteria	Firmicutes	Clostridia	Clostridiales	Defluviitaleaceae	Defluviitaleaceae_UCG-011
21	FALSE	Bacteria	Actinobacteria	Coriobacteriia	Coriobacteriales	Eggerthellaceae	Enterorhabdus
21	FALSE	Bacteria	Proteobacteria	Gammaproteobacteria	Pasteurellales	Pasteurellaceae	Haemophilus
21	FALSE	Bacteria	Proteobacteria	Gammaproteobacteria	Pasteurellales	Pasteurellaceae	NA
21	FALSE	Bacteria	Firmicutes	Negativicutes	Selenomonadales	Veillonellaceae	Negativicoccus
21	FALSE	Bacteria	Firmicutes	Clostridia	Clostridiales	Peptococcaceae	Peptococcus
21	FALSE	Bacteria	Proteobacteria	Gammaproteobacteria	Xanthomonadales	Xanthomonadaceae	Pseudoxanthomonas
21	FALSE	Bacteria	Proteobacteria	Gammaproteobacteria	Enterobacteriales	Enterobacteriaceae	Salmonella
21	FALSE	Bacteria	Actinobacteria	Coriobacteriia	Coriobacteriales	Eggerthellaceae	Senegalimassilia
21	FALSE	Bacteria	Actinobacteria	Actinobacteria	Streptomycetales	Streptomycetaceae	Streptomyces
20	FALSE	Bacteria	Actinobacteria	Actinobacteria	Micrococcales	Dermabacteraceae	Brachybacterium
20	FALSE	Bacteria	Firmicutes	Bacilli	Bacillales	Paenibacillaceae	Brevibacillus
20	FALSE	Bacteria	Firmicutes	Clostridia	Clostridiales	Lachnospiraceae	Eisenbergiella
20	FALSE	Bacteria	Firmicutes	Clostridia	Clostridiales	Lachnospiraceae	Moryella
20	FALSE	Archaea	Euryarchaeota	Thermoplasmata	Methanomassiliicoccales	Methanomethylophilaceae	NA
20	FALSE	Bacteria	Firmicutes	Clostridia	Clostridiales	Christensenellaceae	NA
20	FALSE	Bacteria	Proteobacteria	Gammaproteobacteria	Betaproteobacteriales	Burkholderiaceae	Oligella
20	FALSE	Bacteria	Proteobacteria	Gammaproteobacteria	Enterobacteriales	Enterobacteriaceae	Providencia
20	FALSE	Bacteria	Firmicutes	Clostridia	Clostridiales	Ruminococcaceae	Ruminiclostridium_1
20	FALSE	Bacteria	Firmicutes	Erysipelotrichia	Erysipelotrichales	Erysipelotrichaceae	Solobacterium
19	FALSE	Bacteria	Actinobacteria	Actinobacteria	Micrococcales	Brevibacteriaceae	Brevibacterium
19	FALSE	Bacteria	Firmicutes	Clostridia	Clostridiales	Ruminococcaceae	CAG-352
19	FALSE	Bacteria	Firmicutes	Clostridia	Clostridiales	Lachnospiraceae	Epulopiscium
19	FALSE	Bacteria	Firmicutes	Clostridia	Clostridiales	Ruminococcaceae	Hydrogenoanaerobacterium
19	FALSE	Bacteria	Bacteroidetes	Bacteroidia	Bacteroidales	Prevotellaceae	Prevotella_6
19	FALSE	Bacteria	Synergistetes	Synergistia	Synergistales	Synergistaceae	Pyramidobacter
18	FALSE	Bacteria	Firmicutes	Erysipelotrichia	Erysipelotrichales	Erysipelotrichaceae	Catenibacterium
18	FALSE	Bacteria	Proteobacteria	Gammaproteobacteria	Enterobacteriales	Enterobacteriaceae	Edwardsiella
18	FALSE	Bacteria	Firmicutes	Clostridia	Clostridiales	Ruminococcaceae	GCA-900066225
18	FALSE	Bacteria	Firmicutes	Bacilli	Bacillales	Planococcaceae	Lysinibacillus
18	FALSE	Bacteria	Actinobacteria	Actinobacteria	Micrococcales	Micrococcaceae	Micrococcus
18	FALSE	Bacteria	Bacteroidetes	Bacteroidia	Bacteroidales	Prevotellaceae	NA
18	FALSE	Bacteria	Bacteroidetes	Bacteroidia	Bacteroidales	Prevotellaceae	Prevotella_2
18	FALSE	Bacteria	Firmicutes	Clostridia	Clostridiales	Lachnospiraceae	Sellimonas

17	FALSE	Bacteria	Bacteroidetes	Bacteroidia	Bacteroidales	Barnesiellaceae	Coprobacter
17	FALSE	Bacteria	Firmicutes	Negativicutes	Selenomonadales	Veillonellaceae	Megasphaera
17	FALSE	Bacteria	Actinobacteria	Actinobacteria	Corynebacteriales	Corynebacteriaceae	NA
17	FALSE	Bacteria	Bacteroidetes	Bacteroidia	Bacteroidales	Muribaculaceae	NA
17	FALSE	Bacteria	Firmicutes	Clostridia	DTU014	NA	NA
17	FALSE	Bacteria	Firmicutes	Bacilli	Bacillales	Bacillaceae	Pseudogracilibacillus
17	FALSE	Bacteria	Firmicutes	Clostridia	Clostridiales	Peptostreptococcaceae	Terrisporobacter
16	FALSE	Bacteria	Firmicutes	Negativicutes	Selenomonadales	Acidaminococcaceae	Acidaminococcus
16	FALSE	Bacteria	Bacteroidetes	Bacteroidia	Bacteroidales	Marinifilaceae	Butyricimonas
16	FALSE	Bacteria	Firmicutes	Clostridia	Clostridiales	Lachnospiraceae	Butyrivibrio
16	FALSE	Bacteria	Actinobacteria	Actinobacteria	Bifidobacteriales	Bifidobacteriaceae	Gardnerella
16	FALSE	Bacteria	Firmicutes	Clostridia	Clostridiales	Peptococcaceae	NA
15	FALSE	Bacteria	Actinobacteria	Actinobacteria	Corynebacteriales	Corynebacteriaceae	Corynebacterium
15	FALSE	Bacteria	Firmicutes	Erysipelotrichia	Erysipelotrichales	Erysipelotrichaceae	Faecalitalea
15	FALSE	Bacteria	Actinobacteria	Actinobacteria	Micrococcales	Micrococcaceae	Kocuria
15	FALSE	Archaea	Euryarchaeota	Methanobacteria	Methanobacteriales	Methanobacteriaceae	Methanosphaera
15	FALSE	Bacteria	Firmicutes	Clostridia	Clostridiales	NA	NA
15	FALSE	Bacteria	Actinobacteria	Actinobacteria	Propionibacteriales	Propionibacteriaceae	Propionibacterium
15	FALSE	Bacteria	Firmicutes	Clostridia	Clostridiales	Ruminococcaceae	Ruminococcaceae_UCG-005
14	FALSE	Bacteria	Firmicutes	Clostridia	Clostridiales	Ruminococcaceae	Acetanaerobacterium
14	FALSE	Bacteria	Firmicutes	Clostridia	Clostridiales	Ruminococcaceae	Anaerofilum
14	FALSE	Bacteria	Synergistetes	Synergistia	Synergistales	Synergistaceae	Cloacibacillus
14	FALSE	Bacteria	Firmicutes	Bacilli	Lactobacillales	Leuconostocaceae	Leuconostoc
14	FALSE	Bacteria	Firmicutes	Clostridia	NA	NA	NA
14	FALSE	Bacteria	Firmicutes	Clostridia	Clostridiales	Peptostreptococcaceae	Peptoclostridium
14	FALSE	Bacteria	Firmicutes	Clostridia	Clostridiales	Ruminococcaceae	Ruminiclostridium_5
14	FALSE	Bacteria	Firmicutes	Negativicutes	Selenomonadales	Acidaminococcaceae	Succiniclasticum
13	FALSE	Bacteria	Proteobacteria	Alphaproteobacteria	Rhizobiales	Rhizobiaceae	Brucella
13	FALSE	Bacteria	Firmicutes	Clostridia	Clostridiales	Lachnospiraceae	Lactonifactor
13	FALSE	Bacteria	Bacteroidetes	Bacteroidia	Bacteroidales	Barnesiellaceae	NA
13	FALSE	Bacteria	Patescibacteria	Saccharimonadia	Saccharimonadales	NA	NA
13	FALSE	Bacteria	Tenericutes	Mollicutes	Mollicutes_RF39	NA	NA
13	FALSE	Bacteria	Bacteroidetes	Bacteroidia	Bacteroidales	Prevotellaceae	Prevotellaceae_NK3B31_group
13	FALSE	Bacteria	Firmicutes	Clostridia	Clostridiales	Ruminococcaceae	Ruminiclostridium
12	FALSE	Bacteria	Firmicutes	Clostridia	Clostridiales	Ruminococcaceae	Angelakisella

12	FALSE	Bacteria	Firmicutes	Clostridia	Clostridiales	Ruminococcaceae	Caproiciproducens
12	FALSE	Bacteria	Firmicutes	Clostridia	Clostridiales	Christensenellaceae	Catabacter
12	FALSE	Bacteria	Firmicutes	Erysipelotrichia	Erysipelotrichales	Erysipelotrichaceae	Coprobacillus
12	FALSE	Bacteria	Firmicutes	Clostridia	Clostridiales	Lachnospiraceae	GCA-900066755
12	FALSE	Bacteria	Firmicutes	Clostridia	Clostridiales	Lachnospiraceae	Lachnospiraceae_NK4B4_group
12	FALSE	Bacteria	Firmicutes	Negativicutes	Selenomonadales	Veillonellaceae	NA
12	FALSE	Bacteria	Firmicutes	Clostridia	Clostridiales	Family_XIII	NA
12	FALSE	Bacteria	Tenericutes	Mollicutes	Izimaplasmatales	NA	NA
12	FALSE	Bacteria	Proteobacteria	Gammaproteobacteria	Betaproteobacteriales	Burkholderiaceae	Oxalobacter
12	FALSE	Bacteria	Firmicutes	Clostridia	Clostridiales	Ruminococcaceae	Ruminococcaceae_UCG-004
12	FALSE	Bacteria	Firmicutes	Bacilli	Lactobacillales	Leuconostocaceae	Weissella
11	FALSE	Bacteria	Actinobacteria	Coriobacteriia	Coriobacteriales	Eggerthellaceae	Adlercreutzia
11	FALSE	Bacteria	Firmicutes	Clostridia	Clostridiales	Christensenellaceae	Christensenellaceae_R-7_group
11	FALSE	Bacteria	Actinobacteria	Coriobacteriia	Coriobacteriales	Eggerthellaceae	Gordonibacter
11	FALSE	Bacteria	Firmicutes	Clostridia	Clostridiales	Ruminococcaceae	Harryflintia
11	FALSE	Bacteria	Firmicutes	Clostridia	Clostridiales	Lachnospiraceae	Lachnospiraceae_FCS020_group
11	FALSE	Bacteria	Firmicutes	Clostridia	Clostridiales	Lachnospiraceae	Lachnospiraceae_UCG-010
11	FALSE	Bacteria	Firmicutes	NA	NA	NA	NA
11	FALSE	Bacteria	Bacteroidetes	Bacteroidia	NA	NA	NA
11	FALSE	Bacteria	Actinobacteria	Actinobacteria	Micrococcales	Micrococcaceae	Rothia
11	FALSE	Bacteria	Firmicutes	Clostridia	Clostridiales	Lachnospiraceae	Shuttleworthia
10	FALSE	Bacteria	Firmicutes	Bacilli	Lactobacillales	Streptococcaceae	Lactococcus
10	FALSE	Bacteria	Firmicutes	Bacilli	Bacillales	Bacillaceae	NA
10	FALSE	Bacteria	Firmicutes	Bacilli	Bacillales	Paenibacillaceae	Paenibacillus
10	FALSE	Bacteria	Actinobacteria	Coriobacteriia	Coriobacteriales	Eggerthellaceae	Slackia
9	FALSE	Bacteria	Firmicutes	Erysipelotrichia	Erysipelotrichales	Erysipelotrichaceae	Candidatus_Stoquefichus
9	FALSE	Bacteria	Firmicutes	Clostridia	Clostridiales	Family_XIII	Family_XIII_AD3011_group
9	FALSE	Bacteria	Firmicutes	Negativicutes	Selenomonadales	Veillonellaceae	Megamonas
9	FALSE	Bacteria	Patescibacteria	Saccharimonadia	Saccharimonadales	Saccharimonadaceae	NA
9	FALSE	Bacteria	Actinobacteria	Coriobacteriia	Coriobacteriales	Coriobacteriales_Incertae_Sedis	NA
9	FALSE	Bacteria	Proteobacteria	Deltaproteobacteria	Desulfovibrionales	Desulfovibrionaceae	NA
9	FALSE	Bacteria	Firmicutes	Bacilli	Bacillales	Bacillaceae	Oceanobacillus
9	FALSE	Bacteria	Firmicutes	Clostridia	Clostridiales	Ruminococcaceae	Oscillibacter
9	FALSE	Bacteria	Firmicutes	Clostridia	Clostridiales	Peptostreptococcaceae	Romboutsia
9	FALSE	Bacteria	Proteobacteria	Gammaproteobacteria	Betaproteobacteriales	Burkholderiaceae	Sutterella

8	FALSE	Bacteria	Actinobacteria	Coriobacteriia	Coriobacteriales	Eggerthellaceae	Eggerthella
8	FALSE	Bacteria	Firmicutes	Clostridia	Clostridiales	Ruminococcaceae	Flavonifractor
8	FALSE	Bacteria	Proteobacteria	Gammaproteobacteria	Enterobacteriales	Enterobacteriaceae	Morganella
8	FALSE	Bacteria	Firmicutes	Bacilli	Bacillales	Planococcaceae	NA
8	FALSE	Bacteria	Bacteroidetes	Bacteroidia	Bacteroidales	NA	NA
8	FALSE	Bacteria	Cyanobacteria	Melainabacteria	Gastranaerophilales	NA	NA
8	FALSE	Bacteria	Firmicutes	Clostridia	Clostridiales	Lachnospiraceae	Tyzzerella_3
7	FALSE	Bacteria	Firmicutes	Clostridia	Clostridiales	Lachnospiraceae	Coprococcus_1
7	FALSE	Bacteria	Firmicutes	Clostridia	Clostridiales	Lachnospiraceae	Marvinbryantia
7	FALSE	Bacteria	Bacteroidetes	Bacteroidia	Bacteroidales	Prevotellaceae	Prevotella_7
7	FALSE	Bacteria	Firmicutes	Clostridia	Clostridiales	Ruminococcaceae	Ruminococcaceae_UCG-010
7	FALSE	Bacteria	Firmicutes	Bacilli	Bacillales	Staphylococcaceae	Staphylococcus
7	FALSE	Bacteria	Firmicutes	Clostridia	Clostridiales	Lachnospiraceae	UC5-1-2E3
6	FALSE	Bacteria	Proteobacteria	Deltaproteobacteria	Desulfovibrionales	Desulfovibrionaceae	Bilophila
6	FALSE	Bacteria	Firmicutes	Clostridia	Clostridiales	Lachnospiraceae	CAG-56
6	FALSE	Bacteria	Firmicutes	Clostridia	Clostridiales	Lachnospiraceae	Coprococcus_2
6	FALSE	Bacteria	Firmicutes	Clostridia	Clostridiales	Clostridiales_vadinBB60_group	NA
6	FALSE	Bacteria	Bacteroidetes	Bacteroidia	Bacteroidales	Tannerellaceae	Parabacteroides
6	FALSE	Bacteria	Bacteroidetes	Bacteroidia	Bacteroidales	Prevotellaceae	Prevotella_9
5	FALSE	Bacteria	Bacteroidetes	Bacteroidia	Bacteroidales	Rikenellaceae	Alistipes
5	FALSE	Bacteria	Firmicutes	Negativicutes	Selenomonadales	Veillonellaceae	Dialister
5	FALSE	Bacteria	Firmicutes	Clostridia	Clostridiales	Lachnospiraceae	Dorea
4	FALSE	Bacteria	Firmicutes	Clostridia	Clostridiales	Lachnospiraceae	Lachnospiraceae_NK4A136_group
4	FALSE	Bacteria	Firmicutes	Negativicutes	Selenomonadales	Acidaminococcaceae	Phascolarctobacterium
3	FALSE	Bacteria	Proteobacteria	Gammaproteobacteria	Enterobacteriales	Enterobacteriaceae	Escherichia/Shigella
3	FALSE	Bacteria	Proteobacteria	Alphaproteobacteria	Rhodospirillales	NA	NA
3	FALSE	Bacteria	Proteobacteria	Gammaproteobacteria	Betaproteobacteriales	Burkholderiaceae	Parasutterella
2	FALSE	Bacteria	Actinobacteria	Coriobacteriia	Coriobacteriales	Coriobacteriaceae	Collinsella
2	FALSE	Bacteria	Firmicutes	Clostridia	Clostridiales	Ruminococcaceae	Fournierella
2	FALSE	Bacteria	Proteobacteria	Gammaproteobacteria	Enterobacteriales	Enterobacteriaceae	Klebsiella
2	FALSE	Bacteria	Bacteroidetes	Bacteroidia	Bacteroidales	Marinifilaceae	Odoribacter
2	FALSE	Bacteria	Bacteroidetes	Bacteroidia	Bacteroidales	Prevotellaceae	Paraprevotella
1	FALSE	Bacteria	Firmicutes	Clostridia	Clostridiales	Clostridiaceae_1	Clostridium_sensu_stricto_1
1	FALSE	Bacteria	Firmicutes	Clostridia	Clostridiales	Lachnospiraceae	Coprococcus_3
1	FALSE	Bacteria	Firmicutes	Bacilli	Lactobacillales	Enterococcaceae	Enterococcus

1	FALSE	Bacteria	Firmicutes	Clostridia	Clostridiales	Peptostreptococcaceae	Intestinibacter
1	FALSE	Bacteria	Firmicutes	Clostridia	Clostridiales	Ruminococcaceae	Negativibacillus
1	FALSE	Bacteria	Proteobacteria	Gammaproteobacteria	Enterobacteriales	Enterobacteriaceae	Proteus
1	FALSE	Bacteria	Firmicutes	Clostridia	Clostridiales	Ruminococcaceae	Ruminococcaceae_NK4A214_group
1	FALSE	Bacteria	Firmicutes	Clostridia	Clostridiales	Ruminococcaceae	Ruminococcaceae_UCG-003
0	FALSE	Bacteria	Verrucomicrobia	Verrucomicrobiae	Verrucomicrobiales	Akkermansiaceae	Akkermansia
0	FALSE	Bacteria	Bacteroidetes	Bacteroidia	Bacteroidales	Barnesiellaceae	Barnesiella
0	FALSE	Bacteria	Proteobacteria	Gammaproteobacteria	Enterobacteriales	Enterobacteriaceae	NA
0	FALSE	Bacteria	Firmicutes	Bacilli	Bacillales	NA	NA
0	FALSE	Bacteria	Firmicutes	Clostridia	Clostridiales	Ruminococcaceae	Ruminococcaceae_UCG-002
0	FALSE	Bacteria	Firmicutes	Clostridia	Clostridiales	Ruminococcaceae	Subdoligranulum
0	FALSE	Bacteria	Firmicutes	Clostridia	Clostridiales	Lachnospiraceae	Tyzzerella

Supplementary Table 4. MWAS of dataset 1 conducted using Kruskal-Wallis

Sample size for KW included all samples: N= 201 cases and 132 controls in dataset 1. MRA= mean relative abundance, FC=fold change in patients (PD MRA/control MRA), P= unadjusted significance, FDR (BH)= false discovery rate, adjusted significance. Unclassified genera and genera present in <10% of subjects were excluded from this analysis.

PD MRA	Control MRA	FC	P	FDR (BH)	Kingdom	Phylum	Class	Order	Family	Genus
0.0005	0.0012	0.37	4E-06	2E-04	Bacteria	Firmicutes	Clostridia	Clostridiales	Lachnospiraceae	Lachnospiraceae_ND3007_group
0.0027	0.0004	6.61	2E-06	2E-04	Bacteria	Firmicutes	Bacilli	Lactobacillales	Lactobacillaceae	Lactobacillus
0.0191	0.0362	0.53	7E-06	2E-04	Bacteria	Firmicutes	Clostridia	Clostridiales	Lachnospiraceae	Agathobacter
0.0153	0.0084	1.83	5E-05	1E-03	Bacteria	Actinobacteria	Actinobacteria	Bifidobacteriales	Bifidobacteriaceae	Bifidobacterium
0.0008	0.0001	13.03	8E-05	1E-03	Bacteria	Synergistetes	Synergistia	Synergistales	Synergistaceae	Cloacibacillus
0.0353	0.0564	0.63	9E-05	1E-03	Bacteria	Firmicutes	Clostridia	Clostridiales	Ruminococcaceae	Faecalibacterium
0.0036	0.0004	8.14	9E-05	1E-03	Bacteria	Firmicutes	Clostridia	Clostridiales	Lachnospiraceae	Hungatella
0.0029	0.0037	0.80	1E-04	1E-03	Bacteria	Firmicutes	Clostridia	Clostridiales	Lachnospiraceae	Lachnospira
0.0047	0.0012	3.77	1E-04	1E-03	Bacteria	Firmicutes	Negativicutes	Selenomonadales	Veillonellaceae	Megasphaera
0.0034	0.0008	4.20	1E-04	1E-03	Bacteria	Bacteroidetes	Bacteroidia	Bacteroidales	Porphyromonadaceae	Porphyromonas
0.0140	0.0205	0.68	2E-04	2E-03	Bacteria	Firmicutes	Clostridia	Clostridiales	Lachnospiraceae	Blautia
0.0017	0.0001	12.72	4E-04	4E-03	Bacteria	Firmicutes	Erysipelotrichia	Erysipelotrichales	Erysipelotrichaceae	Coprobacillus
0.0077	0.0160	0.48	4E-04	4E-03	Bacteria	Firmicutes	Clostridia	Clostridiales	Lachnospiraceae	Roseburia
0.0038	0.0015	2.56	7E-04	6E-03	Bacteria	Bacteroidetes	Bacteroidia	Bacteroidales	Prevotellaceae	Prevotella
0.0541	0.0218	2.48	1E-03	7E-03	Bacteria	Verrucomicrobia	Verrucomicrobiae	Verrucomicrobiales	Akkermansiaceae	Akkermansia
0.0012	0.0019	0.66	1E-03	7E-03	Bacteria	Firmicutes	Clostridia	Clostridiales	Ruminococcaceae	Butyricicoccus
0.0045	0.0023	1.95	1E-03	8E-03	Bacteria	Firmicutes	Clostridia	Clostridiales	Ruminococcaceae	UBA1819
0.0005	0.0001	4.95	2E-03	0.01	Bacteria	Actinobacteria	Actinobacteria	Actinomycetales	Actinomycetaceae	Varibaculum
0.0020	0.0010	1.96	2E-03	0.01	Bacteria	Actinobacteria	Actinobacteria	Corynebacteriales	Corynebacteriaceae	Corynebacterium_1
0.0006	0.0003	1.76	3E-03	0.01	Bacteria	Firmicutes	Clostridia	Clostridiales	Ruminococcaceae	Ruminococcaceae_UCG-004
0.0021	0.0038	0.56	3E-03	0.02	Bacteria	Firmicutes	Clostridia	Clostridiales	Lachnospiraceae	Fusicatenibacter
0.0003	0.0007	0.48	4E-03	0.02	Bacteria	Firmicutes	Clostridia	Clostridiales	Lachnospiraceae	Lachnospiraceae_UCG-004
0.0004	0.0006	0.65	4E-03	0.02	Bacteria	Firmicutes	Clostridia	Clostridiales	Ruminococcaceae	Oscillospira

0.0027	0.0040	0.69	5E-03	0.02	Bacteria	Firmicutes	Clostridia	Clostridiales	Lachnospiraceae	Anaerostipes
0.0015	0.0013	1.16	5E-03	0.02	Bacteria	Proteobacteria	Deltaproteobacteria	Desulfovibrionales	Desulfovibrionaceae	Desulfovibrio
0.0006	0.0002	2.86	6E-03	0.03	Bacteria	Firmicutes	Clostridia	Clostridiales	Ruminococcaceae	Anaerotruncus
0.0006	0.0004	1.37	7E-03	0.03	Archaea	Euryarchaeota	Methanobacteria	Methanobacteriales	Methanobacteriaceae	Methanobrevibacter
0.0071	0.0021	3.32	8E-03	0.03	Bacteria	Firmicutes	Clostridia	Clostridiales	Family_XI	Ezakiella
0.0003	0.0017	0.17	9E-03	0.03	Bacteria	Proteobacteria	Gammaproteobacteria	Pasteurellales	Pasteurellaceae	Haemophilus
0.2148	0.2479	0.87	0.01	0.04	Bacteria	Bacteroidetes	Bacteroidia	Bacteroidales	Bacteroidaceae	Bacteroides
0.0043	0.0029	1.47	0.01	0.05	Bacteria	Proteobacteria	Deltaproteobacteria	Desulfovibrionales	Desulfovibrionaceae	Bilophila
0.0014	0.0018	0.77	0.02	0.07	Bacteria	Firmicutes	Clostridia	Clostridiales	Clostridiaceae_1	Clostridium_sensu_stricto_1
0.0008	0.0013	0.64	0.02	0.07	Bacteria	Firmicutes	Clostridia	Clostridiales	Lachnospiraceae	Coprococcus_3
0.0006	0.0005	1.13	0.03	0.09	Bacteria	Firmicutes	Clostridia	Clostridiales	Family_XIII	Family_XIII_AD3011_group
0.0012	0.0009	1.32	0.03	0.10	Bacteria	Firmicutes	Clostridia	Clostridiales	Ruminococcaceae	Ruminococcaceae_UCG-010
0.0013	0.0014	0.95	0.03	0.10	Bacteria	Firmicutes	Clostridia	Clostridiales	Ruminococcaceae	Ruminococcaceae_UCG-013
0.0021	0.0014	1.42	0.04	0.10	Bacteria	Firmicutes	Clostridia	Clostridiales	Lachnospiraceae	Eisenbergiella
0.0023	0.0009	2.58	0.04	0.10	Bacteria	Firmicutes	Clostridia	Clostridiales	Family_XI	Peptoniphilus
0.0008	0.0002	3.81	0.04	0.10	Bacteria	Epsilonbacteraeota	Campylobacteria	Campylobacterales	Campylobacteraceae	Campylobacter
0.0003	0.0001	2.12	0.04	0.11	Bacteria	Firmicutes	Clostridia	Clostridiales	Family_XI	Murdochiella
0.0052	0.0079	0.66	0.04	0.12	Bacteria	Firmicutes	Negativicutes	Selenomonadales	Acidaminococcaceae	Phascolarctobacterium
0.0060	0.0092	0.65	0.04	0.12	Bacteria	Firmicutes	Clostridia	Clostridiales	Lachnospiraceae	Lachnoclostridium
0.0020	0.0005	4.09	0.05	0.13	Bacteria	Bacteroidetes	Bacteroidia	Bacteroidales	Prevotellaceae	Prevotella_6
0.0009	0.0011	0.86	0.06	0.15	Bacteria	Firmicutes	Clostridia	Clostridiales	Ruminococcaceae	Intestinimonas
0.0302	0.0233	1.29	0.07	0.15	Bacteria	Bacteroidetes	Bacteroidia	Bacteroidales	Tannerellaceae	Parabacteroides
0.0070	0.0067	1.05	0.06	0.15	Bacteria	Firmicutes	Clostridia	Clostridiales	Ruminococcaceae	Ruminococcaceae_UCG-005
0.0006	0.0001	5.47	0.07	0.16	Bacteria	Firmicutes	Clostridia	Clostridiales	Lachnospiraceae	Sellimonas
0.0015	0.0004	3.31	0.08	0.17	Bacteria	Firmicutes	Clostridia	Clostridiales	Family_XI	Anaerococcus
0.0020	0.0031	0.67	0.08	0.17	Bacteria	Firmicutes	Erysipelotrichia	Erysipelotrichales	Erysipelotrichaceae	Erysipelotrichaceae_UCG-003
0.0036	0.0032	1.14	0.08	0.17	Bacteria	Firmicutes	Clostridia	Clostridiales	Ruminococcaceae	Oscillibacter
0.0015	0.0022	0.69	0.09	0.20	Bacteria	Firmicutes	Clostridia	Clostridiales	Ruminococcaceae	Ruminococcaceae_UCG-003
0.0002	0.0002	0.80	0.11	0.23	Bacteria	Actinobacteria	Coriobacteriia	Coriobacteriales	Eggerthellaceae	Eggerthella
0.0002	0.0001	3.08	0.11	0.23	Bacteria	Firmicutes	Clostridia	Clostridiales	Ruminococcaceae	Hydrogenoanaerobacterium

0.0020	0.0016	1.28	0.12	0.24	Bacteria	Bacteroidetes	Bacteroidia	Bacteroidales	Marinifilaceae	Butyricimonas
0.0032	0.0014	2.30	0.13	0.25	Bacteria	Fusobacteria	Fusobacteriia	Fusobacteriales	Fusobacteriaceae	Fusobacterium
0.0027	0.0014	1.96	0.13	0.25	Bacteria	Firmicutes	Erysipelotrichia	Erysipelotrichales	Erysipelotrichaceae	Holdemanella
0.0038	0.0024	1.60	0.14	0.27	Bacteria	Firmicutes	Clostridia	Clostridiales	Ruminococcaceae	Ruminiclostridium_5
0.0039	0.0022	1.74	0.15	0.27	Bacteria	Firmicutes	Negativicutes	Selenomonadales	Acidaminococcaceae	Acidaminococcus
0.0058	0.0034	1.69	0.15	0.27	Bacteria	Firmicutes	Clostridia	Clostridiales	Lachnospiraceae	Tyzzerella_4
0.0386	0.0337	1.15	0.16	0.28	Bacteria	Bacteroidetes	Bacteroidia	Bacteroidales	Rikenellaceae	Alistipes
0.0015	0.0004	3.79	0.16	0.28	Bacteria	Firmicutes	Erysipelotrichia	Erysipelotrichales	Erysipelotrichaceae	Faecalitalea
0.0003	0.0001	4.63	0.16	0.28	Bacteria	Lentisphaerae	Lentisphaeria	Victivallales	Victivallaceae	Victivallis
0.0003	0.0005	0.57	0.17	0.29	Bacteria	Firmicutes	Clostridia	Clostridiales	Lachnospiraceae	CAG-56
0.0006	0.0009	0.64	0.18	0.30	Bacteria	Firmicutes	Clostridia	Clostridiales	Lachnospiraceae	Lachnospiraceae_UCG-010
0.0161	0.0109	1.48	0.19	0.32	Bacteria	Firmicutes	Clostridia	Clostridiales	Ruminococcaceae	Ruminococcaceae_UCG-002
0.0010	0.0007	1.45	0.24	0.39	Bacteria	Firmicutes	Clostridia	Clostridiales	Family_XI	Finegoldia
0.0002	0.0003	0.65	0.25	0.40	Bacteria	Firmicutes	Clostridia	Clostridiales	Ruminococcaceae	GCA-900066225
0.0065	0.0046	1.41	0.26	0.42	Bacteria	Firmicutes	Clostridia	Clostridiales	Christensenellaceae	Christensenellaceae_R-7_group
0.0004	0.0001	3.97	0.27	0.42	Bacteria	Firmicutes	Erysipelotrichia	Erysipelotrichales	Erysipelotrichaceae	Dielma
0.0072	0.0071	1.02	0.28	0.44	Bacteria	Firmicutes	Clostridia	Clostridiales	Ruminococcaceae	Ruminococcus_1
0.0024	0.0029	0.83	0.29	0.45	Bacteria	Firmicutes	Clostridia	Clostridiales	Lachnospiraceae	Dorea
0.0002	0.0003	0.75	0.31	0.48	Bacteria	Firmicutes	Clostridia	Clostridiales	Ruminococcaceae	DTU089
0.0015	0.0013	1.15	0.32	0.48	Bacteria	Firmicutes	Clostridia	Clostridiales	Peptostreptococcaceae	Intestinibacter
0.0038	0.0032	1.19	0.32	0.48	Bacteria	Firmicutes	Clostridia	Clostridiales	Ruminococcaceae	Ruminococcaceae_NK4A214_group
0.0033	0.0040	0.82	0.34	0.50	Bacteria	Proteobacteria	Gammaproteobacteria	Betaproteobacteriales	Burkholderiaceae	Sutterella
0.0077	0.0082	0.94	0.36	0.52	Bacteria	Firmicutes	Clostridia	Clostridiales	Ruminococcaceae	Ruminococcaceae_UCG-014
0.0023	0.0023	0.99	0.39	0.55	Bacteria	Bacteroidetes	Bacteroidia	Bacteroidales	Marinifilaceae	Odoribacter
0.0004	0.0004	0.94	0.43	0.59	Bacteria	Firmicutes	Erysipelotrichia	Erysipelotrichales	Erysipelotrichaceae	Holdemania
0.0015	0.0012	1.26	0.43	0.60	Bacteria	Firmicutes	Clostridia	Clostridiales	Peptostreptococcaceae	Romboutsia
0.0006	0.0005	1.19	0.44	0.60	Bacteria	Firmicutes	Clostridia	Clostridiales	Lachnospiraceae	Coprococcus_1
0.0087	0.0126	0.69	0.45	0.60	Bacteria	Proteobacteria	Gammaproteobacteria	Pseudomonadales	Moraxellaceae	Acinetobacter
0.0005	0.0008	0.66	0.45	0.60	Bacteria	Firmicutes	Erysipelotrichia	Erysipelotrichales	Erysipelotrichaceae	Erysipelatoclostridium
0.0003	0.0005	0.51	0.47	0.61	Bacteria	Bacteroidetes	Bacteroidia	Bacteroidales	Barnesiellaceae	Coprobacter

0.0020	0.0018	1.10	0.47	0.61	Bacteria	Firmicutes	Clostridia	Clostridiales	Ruminococcaceae	Negativibacillus
0.0010	0.0029	0.35	0.46	0.61	Bacteria	Bacteroidetes	Bacteroidia	Bacteroidales	Prevotellaceae	Prevotella_7
0.0042	0.0054	0.79	0.49	0.62	Bacteria	Firmicutes	Clostridia	Clostridiales	Lachnospiraceae	Lachnospiraceae_NK4A136_group
0.0040	0.0023	1.72	0.50	0.63	Bacteria	Firmicutes	Bacilli	Lactobacillales	Streptococcaceae	Streptococcus
0.0064	0.0058	1.12	0.52	0.64	Bacteria	Bacteroidetes	Bacteroidia	Bacteroidales	Barnesiellaceae	Barnesiella
0.0121	0.0133	0.90	0.52	0.64	Bacteria	Firmicutes	Clostridia	Clostridiales	Ruminococcaceae	Subdoligranulum
0.0007	0.0005	1.44	0.56	0.68	Bacteria	Firmicutes	Erysipelotrichia	Erysipelotrichales	Erysipelotrichaceae	Turicibacter
0.0024	0.0026	0.90	0.58	0.69	Bacteria	Bacteroidetes	Bacteroidia	Bacteroidales	Prevotellaceae	Paraprevotella
0.0010	0.0009	1.10	0.59	0.70	Bacteria	Actinobacteria	Coriobacteriia	Coriobacteriales	Coriobacteriaceae	Collinsella
0.0026	0.0024	1.08	0.60	0.70	Bacteria	Firmicutes	Clostridia	Clostridiales	Ruminococcaceae	Ruminiclostridium_9
0.0012	0.0006	1.95	0.61	0.70	Bacteria	Firmicutes	Negativicutes	Selenomonadales	Veillonellaceae	Veillonella
0.0008	0.0005	1.45	0.62	0.71	Bacteria	Firmicutes	Bacilli	Lactobacillales	Enterococcaceae	Enterococcus
0.0001	0.0002	0.82	0.63	0.72	Bacteria	Firmicutes	Clostridia	Clostridiales	Ruminococcaceae	Angelakisella
0.1131	0.1351	0.84	0.64	0.72	Bacteria	Proteobacteria	Gammaproteobacteria	Enterobacteriales	Enterobacteriaceae	Escherichia/Shigella
0.0021	0.0014	1.47	0.64	0.72	Bacteria	Firmicutes	Clostridia	Clostridiales	Lachnospiraceae	Tyzzerella
0.0017	0.0010	1.74	0.74	0.81	Bacteria	Firmicutes	Clostridia	Clostridiales	Ruminococcaceae	CAG-352
0.0211	0.0155	1.37	0.78	0.85	Bacteria	Proteobacteria	Gammaproteobacteria	Pseudomonadales	Pseudomonadaceae	Pseudomonas
0.0079	0.0090	0.89	0.81	0.86	Bacteria	Firmicutes	Negativicutes	Selenomonadales	Veillonellaceae	Dialister
0.0024	0.0026	0.92	0.81	0.86	Bacteria	Firmicutes	Clostridia	Clostridiales	Ruminococcaceae	Flavonifractor
0.0039	0.0047	0.83	0.82	0.86	Bacteria	Proteobacteria	Gammaproteobacteria	Betaproteobacteriales	Burkholderiaceae	Parasutterella
0.0115	0.0080	1.45	0.81	0.86	Bacteria	Firmicutes	Clostridia	Clostridiales	Ruminococcaceae	Ruminococcus_2
0.0040	0.0052	0.76	0.85	0.88	Bacteria	Firmicutes	Clostridia	Clostridiales	Ruminococcaceae	Ruminiclostridium_6
0.0006	0.0007	0.81	0.88	0.90	Bacteria	Firmicutes	Clostridia	Clostridiales	Lachnospiraceae	Lachnospiraceae_UCG-001
0.0106	0.0155	0.68	0.88	0.90	Bacteria	Bacteroidetes	Bacteroidia	Bacteroidales	Prevotellaceae	Prevotella_9
0.0002	0.0002	0.94	0.91	0.92	Bacteria	Firmicutes	Clostridia	Clostridiales	Lachnospiraceae	GCA-900066575
0.0020	0.0015	1.36	1.00	1.00	Bacteria	Firmicutes	Clostridia	Clostridiales	Lachnospiraceae	Coprococcus_2

Supplementary Table 5. MWAS of dataset 2 conducted using Kruskal-Wallis

Sample size for KW included all samples: N= 323 cases and 184 controls in dataset 2. MRA= mean relative abundance, FC=fold change in patients (PD MRA/control MRA), P= unadjusted significance, FDR (BH)= false discovery rate, adjusted significance. Unclassified genera and genera present in <10% of subjects were excluded from this analysis. MRA values of 0.0000 correspond to MRAs that were <0.0001.

PD MRA	Control MRA	FC	P	FDR (BH)	Kingdom	Phylum	Class	Order	Family	Genus
0.0239	0.0088	2.72	4E-09	6E-07	Bacteria	Actinobacteria	Actinobacteria	Bifidobacteriales	Bifidobacteriaceae	Bifidobacterium
0.0004	0.0011	0.38	2E-07	1E-05	Bacteria	Firmicutes	Clostridia	Clostridiales	Lachnospiraceae	Lachnospiraceae_UCG-004
0.0097	0.0172	0.56	1E-06	6E-05	Bacteria	Firmicutes	Clostridia	Clostridiales	Lachnospiraceae	Agathobacter
0.0047	0.0078	0.60	7E-06	3E-04	Bacteria	Firmicutes	Clostridia	Clostridiales	Lachnospiraceae	Roseburia
0.0002	0.0001	2.19	8E-06	3E-04	Bacteria	Firmicutes	Clostridia	Clostridiales	Eubacteriaceae	Eubacterium
0.0007	0.0011	0.59	2E-05	6E-04	Bacteria	Firmicutes	Clostridia	Clostridiales	Lachnospiraceae	Lachnospiraceae_ND3007_group
0.0013	0.0018	0.73	3E-05	7E-04	Bacteria	Firmicutes	Clostridia	Clostridiales	Ruminococcaceae	Ruminococcaceae_UCG-013
0.0039	0.0050	0.78	7E-05	1E-03	Bacteria	Firmicutes	Clostridia	Clostridiales	Lachnospiraceae	Anaerostipes
0.0003	0.0001	4.43	2E-04	3E-03	Bacteria	Actinobacteria	Actinobacteria	Corynebacteriales	Corynebacteriaceae	Lawsonella
0.0276	0.0416	0.66	2E-04	3E-03	Bacteria	Firmicutes	Clostridia	Clostridiales	Ruminococcaceae	Faecalibacterium
0.0006	0.0003	2.11	4E-04	0.01	Bacteria	Firmicutes	Erysipelotrichia	Erysipelotrichales	Erysipelotrichaceae	Turicibacter
0.0208	0.0258	0.81	6E-04	0.01	Bacteria	Proteobacteria	Gammaproteobacteria	Pseudomonadales	Pseudomonadaceae	Pseudomonas
0.0036	0.0026	1.38	7E-04	0.01	Bacteria	Firmicutes	Clostridia	Clostridiales	Ruminococcaceae	UBA1819
0.0039	0.0015	2.53	7E-04	0.01	Bacteria	Actinobacteria	Actinobacteria	Corynebacteriales	Corynebacteriaceae	Corynebacterium_1
0.0001	0.0002	0.58	8E-04	0.01	Bacteria	Firmicutes	Erysipelotrichia	Erysipelotrichales	Erysipelotrichaceae	Erysipelotrichaceae_UCG-003
0.0017	0.0007	2.56	1E-03	0.01	Bacteria	Firmicutes	Clostridia	Clostridiales	Family_XI	Anaerococcus
0.0003	0.0007	0.39	1E-03	0.01	Bacteria	Firmicutes	Clostridia	Clostridiales	Lachnospiraceae	Lachnospiraceae_UCG-001
0.0015	0.0008	1.81	1E-03	0.01	Bacteria	Proteobacteria	Deltaproteobacteria	Desulfovibrionales	Desulfovibrionaceae	Desulfovibrio
0.0056	0.0036	1.57	1E-03	0.01	Bacteria	Firmicutes	Bacilli	Lactobacillales	Lactobacillaceae	Lactobacillus
0.0036	0.0053	0.68	2E-03	0.01	Bacteria	Firmicutes	Clostridia	Clostridiales	Lachnospiraceae	Lachnospira
0.0003	0.0005	0.64	2E-03	0.01	Bacteria	Firmicutes	Clostridia	Clostridiales	Ruminococcaceae	Oscillospira
0.0005	0.0003	1.79	2E-03	0.01	Bacteria	Actinobacteria	Actinobacteria	Actinomycetales	Actinomycetaceae	Varibaculum
0.0027	0.0012	2.14	2E-03	0.01	Bacteria	Firmicutes	Clostridia	Clostridiales	Family_XI	Peptoniphilus
0.0015	0.0008	1.77	2E-03	0.02	Bacteria	Firmicutes	Clostridia	Clostridiales	Lachnospiraceae	Hungatella
0.0045	0.0028	1.61	3E-03	0.02	Archaea	Euryarchaeota	Methanobacteria	Methanobacteriales	Methanobacteriaceae	Methanobrevibacter
0.0022	0.0000	220.2	3E-03	0.02	Bacteria	Proteobacteria	Gammaproteobacteria	Betaproteobacteriales	Burkholderiaceae	Delftia
0.0085	0.0086	0.99	3E-03	0.02	Bacteria	Firmicutes	Bacilli	Lactobacillales	Streptococcaceae	Streptococcus
0.0026	0.0009	2.94	3E-03	0.02	Bacteria	Bacteroidetes	Bacteroidia	Bacteroidales	Porphyromonadaceae	Porphyromonas
0.0025	0.0006	4.39	3E-03	0.02	Bacteria	Bacteroidetes	Bacteroidia	Bacteroidales	Prevotellaceae	Prevotella
0.0031	0.0046	0.69	0.01	0.03	Bacteria	Firmicutes	Clostridia	Clostridiales	Lachnospiraceae	Fusicatenibacter
0.0008	0.0005	1.69	0.01	0.03	Bacteria	Firmicutes	Clostridia	Clostridiales	Ruminococcaceae	Anaerotruncus
0.0079	0.0101	0.78	0.01	0.03	Bacteria	Firmicutes	Clostridia	Clostridiales	Ruminococcaceae	Ruminococcus_2

0.0001	0.0000	34.45	0.01	0.03	Bacteria	Firmicutes	Clostridia	Clostridiales	Family_XI	Parvimonas
0.0003	0.0002	1.72	0.01	0.04	Bacteria	Actinobacteria	Actinobacteria	Actinomycetales	Actinomycetaceae	Mobiluncus
0.0005	0.0003	1.45	0.01	0.04	Bacteria	Actinobacteria	Actinobacteria	Actinomycetales	Actinomycetaceae	Actinomyces
0.0012	0.0005	2.60	0.01	0.04	Bacteria	Firmicutes	Clostridia	Clostridiales	Family_XI	Finegoldia
0.0003	0.0001	2.69	0.01	0.04	Bacteria	Firmicutes	Clostridia	Clostridiales	Family_XIII	S5-A14a
0.0187	0.0237	0.79	0.01	0.04	Bacteria	Firmicutes	Clostridia	Clostridiales	Lachnospiraceae	Blautia
0.0005	0.0002	2.25	0.01	0.04	Bacteria	Firmicutes	Clostridia	Clostridiales	Family_XI	Murdochiella
0.0048	0.0015	3.18	0.01	0.04	Bacteria	Firmicutes	Clostridia	Clostridiales	Family_XI	Ezakiella
0.0004	0.0005	0.78	0.01	0.04	Bacteria	Firmicutes	Clostridia	Clostridiales	Ruminococcaceae	DTU089
0.0000	0.0000	2.06	0.01	0.05	Bacteria	Actinobacteria	Actinobacteria	Propionibacteriales	Propionibacteriaceae	Cutibacterium
0.0013	0.0019	0.68	0.02	0.06	Bacteria	Firmicutes	Clostridia	Clostridiales	Ruminococcaceae	Butyricicoccus
0.0000	0.0000	2.91	0.02	0.07	Bacteria	Actinobacteria	Actinobacteria	Bifidobacteriales	Bifidobacteriaceae	Scardovia
0.0034	0.0053	0.64	0.02	0.07	Bacteria	Firmicutes	Clostridia	Clostridiales	Ruminococcaceae	Ruminiclostridium_6
0.0034	0.0044	0.79	0.02	0.07	Bacteria	Firmicutes	Clostridia	Clostridiales	Ruminococcaceae	Ruminococcus_1
0.0000	0.0000	0.72	0.02	0.07	Bacteria	Firmicutes	Bacilli	Bacillales	Family_XI	Gemella
0.0004	0.0001	2.58	0.03	0.09	Bacteria	Firmicutes	Clostridia	Clostridiales	Ruminococcaceae	Fastidiosipila
0.0038	0.0063	0.61	0.03	0.09	Bacteria	Firmicutes	Clostridia	Clostridiales	Ruminococcaceae	Ruminococcaceae_UCG-014
0.0037	0.0018	2.09	0.03	0.09	Bacteria	Firmicutes	Clostridia	Clostridiales	Lachnospiraceae	Eisenbergiella
0.0008	0.0007	1.23	0.03	0.09	Bacteria	Firmicutes	Clostridia	Clostridiales	Ruminococcaceae	Ruminococcaceae_UCG-004
0.0007	0.0002	4.09	0.03	0.09	Bacteria	Epsilonbacteraeota	Campylobacteria	Campylobacterales	Campylobacteraceae	Campylobacter
0.0027	0.0045	0.61	0.03	0.09	Bacteria	Firmicutes	Negativicutes	Selenomonadales	Veillonellaceae	Veillonella
0.0002	0.0000	11.43	0.03	0.10	Bacteria	Lentisphaerae	Lentisphaeria	Victivallales	Victivallaceae	Victivallis
0.0000	0.0001	0.59	0.04	0.12	Bacteria	Firmicutes	Bacilli	Lactobacillales	Carnobacteriaceae	Granulicatella
0.0000	0.0000	0.95	0.04	0.13	Bacteria	Firmicutes	Clostridia	Clostridiales	Lachnospiraceae	Cuneatibacter
0.0001	0.0001	1.45	0.04	0.13	Bacteria	Firmicutes	Clostridia	Clostridiales	Ruminococcaceae	Ruminococcaceae_UCG-009
0.0000	0.0000	1.58	0.05	0.13	Bacteria	Firmicutes	Clostridia	Clostridiales	Ruminococcaceae	Ruminococcaceae_UCG-011
0.0001	0.0001	0.86	0.05	0.14	Bacteria	Firmicutes	Clostridia	Clostridiales	Ruminococcaceae	Candidatus_Soleaferrea
0.0001	0.0002	0.73	0.05	0.14	Bacteria	Proteobacteria	Gammaproteobacteria	Pasteurellales	Pasteurellaceae	Haemophilus
0.0000	0.0000	0.51	0.05	0.14	Bacteria	Firmicutes	Clostridia	Clostridiales	Ruminococcaceae	Ruminococcaceae_UCG-008
0.0001	0.0004	0.40	0.06	0.14	Bacteria	Firmicutes	Clostridia	Clostridiales	Lachnospiraceae	Tyzzerella_3
0.0004	0.0003	1.40	0.06	0.15	Bacteria	Firmicutes	Clostridia	Clostridiales	Ruminococcaceae	GCA-900066225
0.0001	0.0002	0.58	0.06	0.16	Bacteria	Actinobacteria	Actinobacteria	Micrococcales	Brevibacteriaceae	Brevibacterium
0.0002	0.0001	1.42	0.06	0.16	Bacteria	Firmicutes	Clostridia	Clostridiales	Lachnospiraceae	Howardella
0.0023	0.0017	1.37	0.07	0.16	Bacteria	Bacteroidetes	Bacteroidia	Bacteroidales	Marinifilaceae	Butyricimonas
0.0002	0.0001	2.00	0.07	0.17	Bacteria	Firmicutes	Clostridia	Clostridiales	Family_XIII	Mogibacterium
0.0000	0.0000	1.46	0.07	0.17	Bacteria	Firmicutes	Clostridia	Clostridiales	Eubacteriaceae	Anaerofustis
0.0001	0.0001	0.75	0.07	0.17	Bacteria	Firmicutes	Clostridia	Clostridiales	Family_XIII	Family_XIII_UCG-001
0.0096	0.0047	2.06	0.07	0.17	Bacteria	Firmicutes	Negativicutes	Selenomonadales	Acidaminococcaceae	Acidaminococcus
0.0003	0.0002	1.33	0.07	0.17	Bacteria	Firmicutes	Negativicutes	Selenomonadales	Veillonellaceae	Anaeroglobus
0.0001	0.0001	0.82	0.08	0.17	Bacteria	Firmicutes	Clostridia	Clostridiales	Ruminococcaceae	Phocea
0.0001	0.0002	0.76	0.08	0.18	Bacteria	Firmicutes	Clostridia	Clostridiales	Peptococcaceae	Peptococcus

0.0373	0.0460	0.81	0.08	0.18	Bacteria	Firmicutes	Negativicutes	Selenomonadales	Acidaminococcaceae	Phascolarctobacterium
0.0000	0.0000	0.72	0.09	0.19	Bacteria	Firmicutes	Clostridia	Clostridiales	Defluviitaleaceae	Defluviitaleaceae_UCG-011
0.0007	0.0004	1.68	0.10	0.20	Bacteria	Firmicutes	Clostridia	Clostridiales	Lachnospiraceae	Sellimonas
0.0041	0.0032	1.31	0.10	0.21	Bacteria	Firmicutes	Clostridia	Clostridiales	Ruminococcaceae	Ruminococcaceae_UCG-005
0.0000	0.0000	0.65	0.10	0.21	Bacteria	Firmicutes	Clostridia	Clostridiales	Ruminococcaceae	Ruminococcaceae_UCG-007
0.0001	0.0001	0.79	0.10	0.21	Bacteria	Firmicutes	Erysipelotrichia	Erysipelotrichales	Erysipelotrichaceae	Erysipelatoclostridium
0.0036	0.0008	4.50	0.10	0.21	Bacteria	Firmicutes	Clostridia	Clostridiales	Lachnospiraceae	Tyzzerella_4
0.0055	0.0040	1.36	0.10	0.21	Bacteria	Actinobacteria	Coriobacteriia	Coriobacteriales	Coriobacteriaceae	Collinsella
0.1610	0.1887	0.85	0.12	0.23	Bacteria	Proteobacteria	Gammaproteobacteria	Enterobacteriales	Enterobacteriaceae	Escherichia/Shigella
0.0000	0.0000	0.98	0.12	0.23	Bacteria	Firmicutes	Erysipelotrichia	Erysipelotrichales	Erysipelotrichaceae	Holdemania
0.0028	0.0028	0.99	0.14	0.26	Bacteria	Firmicutes	Clostridia	Clostridiales	Lachnospiraceae	Dorea
0.0003	0.0003	0.90	0.15	0.28	Bacteria	Firmicutes	Clostridia	Clostridiales	Lachnospiraceae	CAG-56
0.0004	0.0009	0.40	0.17	0.32	Bacteria	Firmicutes	Clostridia	Clostridiales	Lachnospiraceae	Coprococcus_2
0.0051	0.0040	1.26	0.17	0.33	Bacteria	Firmicutes	Clostridia	Clostridiales	Ruminococcaceae	Ruminiclostridium_5
0.0002	0.0003	0.86	0.18	0.33	Bacteria	Firmicutes	Clostridia	Clostridiales	Lachnospiraceae	GCA-900066575
0.0032	0.0034	0.93	0.19	0.35	Bacteria	Firmicutes	Clostridia	Clostridiales	Lachnospiraceae	Lachnospiraceae_NK4A136_group
0.0001	0.0001	1.63	0.20	0.37	Bacteria	Firmicutes	Erysipelotrichia	Erysipelotrichales	Erysipelotrichaceae	Faecalitalea
0.0032	0.0033	0.99	0.23	0.41	Bacteria	Firmicutes	Clostridia	Clostridiales	Christensenellaceae	Christensenellaceae_R-7_group
0.0199	0.0149	1.34	0.24	0.42	Bacteria	Verrucomicrobia	Verrucomicrobiae	Verrucomicrobiales	Akkermansiaceae	Akkermansia
0.0018	0.0080	0.22	0.24	0.43	Bacteria	Proteobacteria	Gammaproteobacteria	Enterobacteriales	Enterobacteriaceae	Klebsiella
0.0031	0.0032	0.98	0.26	0.46	Bacteria	Firmicutes	Clostridia	Clostridiales	Ruminococcaceae	Ruminiclostridium_9
0.0001	0.0001	1.04	0.27	0.47	Bacteria	Firmicutes	Clostridia	Clostridiales	Lachnospiraceae	Shuttleworthia
0.0001	0.0000	3.41	0.28	0.48	Bacteria	Actinobacteria	Coriobacteriia	Coriobacteriales	Atopobiaceae	Atopobium
0.0104	0.0114	0.91	0.30	0.50	Bacteria	Firmicutes	Clostridia	Clostridiales	Lachnospiraceae	Lachnoclostridium
0.0058	0.0051	1.14	0.32	0.53	Bacteria	Firmicutes	Clostridia	Clostridiales	Ruminococcaceae	Oscillibacter
0.0001	0.0001	1.54	0.32	0.53	Bacteria	Firmicutes	Bacilli	Lactobacillales	Streptococcaceae	Lactococcus
0.1960	0.2043	0.96	0.33	0.53	Bacteria	Bacteroidetes	Bacteroidia	Bacteroidales	Bacteroidaceae	Bacteroides
0.0006	0.0006	0.93	0.34	0.54	Bacteria	Actinobacteria	Coriobacteriia	Coriobacteriales	Eggerthellaceae	Eggerthella
0.0044	0.0033	1.35	0.34	0.55	Bacteria	Proteobacteria	Gammaproteobacteria	Betaproteobacteriales	Burkholderiaceae	Parasutterella
0.0020	0.0021	0.93	0.35	0.55	Bacteria	Firmicutes	Clostridia	Clostridiales	Ruminococcaceae	Ruminococcaceae_UCG-003
0.0000	0.0001	0.37	0.35	0.55	Bacteria	Firmicutes	Clostridia	Clostridiales	Ruminococcaceae	Ruminiclostridium
0.0008	0.0007	1.16	0.35	0.55	Bacteria	Firmicutes	Clostridia	Clostridiales	Lachnospiraceae	Lachnospiraceae_UCG-010
0.0001	0.0001	1.53	0.36	0.55	Bacteria	Firmicutes	Negativicutes	Selenomonadales	Veillonellaceae	Negativicoccus
0.0042	0.0015	2.80	0.36	0.55	Bacteria	Synergistetes	Synergistia	Synergistales	Synergistaceae	Cloacibacillus
0.0018	0.0017	1.08	0.37	0.55	Bacteria	Proteobacteria	Deltaproteobacteria	Desulfovibrionales	Desulfovibrionaceae	Bilophila
0.0026	0.0027	0.98	0.37	0.55	Bacteria	Firmicutes	Clostridia	Clostridiales	Ruminococcaceae	Ruminococcaceae_NK4A214_group
0.0001	0.0001	0.67	0.37	0.55	Bacteria	Firmicutes	Clostridia	Clostridiales	Lachnospiraceae	Lachnospiraceae_FCS020_group
0.0053	0.0037	1.45	0.38	0.56	Bacteria	Firmicutes	Negativicutes	Selenomonadales	Veillonellaceae	Dialister
0.0006	0.0003	1.97	0.40	0.58	Bacteria	Fusobacteria	Fusobacteriia	Fusobacteriales	Fusobacteriaceae	Fusobacterium
0.0002	0.0001	2.54	0.41	0.59	Bacteria	Firmicutes	Clostridia	Clostridiales	Peptostreptococcaceae	Terrisporobacter
0.0015	0.0009	1.67	0.41	0.59	Bacteria	Proteobacteria	Gammaproteobacteria	Betaproteobacteriales	Burkholderiaceae	Sutterella

0.0222	0.0199	1.11	0.41	0.59	Bacteria	Bacteroidetes	Bacteroidia	Bacteroidales	Tannerellaceae	Parabacteroides
0.0020	0.0024	0.83	0.42	0.59	Bacteria	Firmicutes	Clostridia	Clostridiales	Ruminococcaceae	Intestinimonas
0.0006	0.0002	2.78	0.43	0.60	Bacteria	Bacteroidetes	Bacteroidia	Bacteroidales	Prevotellaceae	Prevotella_6
0.0018	0.0020	0.92	0.43	0.60	Bacteria	Firmicutes	Clostridia	Clostridiales	Ruminococcaceae	Negativibacillus
0.0086	0.0035	2.48	0.43	0.60	Bacteria	Firmicutes	Negativicutes	Selenomonadales	Veillonellaceae	Megasphaera
0.0001	0.0001	0.41	0.45	0.61	Bacteria	Firmicutes	Clostridia	Clostridiales	Lachnospiraceae	Lachnospiraceae_NK4B4_group
0.0000	0.0001	0.76	0.45	0.61	Bacteria	Firmicutes	Erysipelotrichia	Erysipelotrichales	Erysipelotrichaceae	Coprobacillus
0.0138	0.0112	1.24	0.46	0.62	Bacteria	Firmicutes	Clostridia	Clostridiales	Ruminococcaceae	Ruminococcaceae_UCG-002
0.0076	0.0131	0.58	0.49	0.64	Bacteria	Proteobacteria	Gammaproteobacteria	Enterobacteriales	Enterobacteriaceae	Proteus
0.0027	0.0020	1.39	0.49	0.64	Bacteria	Firmicutes	Clostridia	Clostridiales	Peptostreptococcaceae	Romboutsia
0.0001	0.0000	1.32	0.49	0.64	Bacteria	Firmicutes	Clostridia	Clostridiales	Ruminococcaceae	Anaerofilum
0.0008	0.0004	2.10	0.50	0.64	Bacteria	Bacteroidetes	Bacteroidia	Bacteroidales	Barnesiellaceae	Coprobacter
0.0006	0.0005	1.29	0.52	0.67	Bacteria	Firmicutes	Bacilli	Bacillales	Staphylococcaceae	Staphylococcus
0.0000	0.0000	1.08	0.52	0.67	Bacteria	Firmicutes	Clostridia	Clostridiales	Christensenellaceae	Catabacter
0.0000	0.0000	1.19	0.53	0.67	Bacteria	Firmicutes	Clostridia	Clostridiales	Lachnospiraceae	GCA-900066755
0.0004	0.0003	1.56	0.54	0.68	Bacteria	Synergistetes	Synergistia	Synergistales	Synergistaceae	Pyramidobacter
0.0001	0.0000	2.06	0.55	0.68	Bacteria	Firmicutes	Clostridia	Clostridiales	Ruminococcaceae	Ruminiclostridium_1
0.0001	0.0000	1.58	0.55	0.68	Bacteria	Firmicutes	Erysipelotrichia	Erysipelotrichales	Erysipelotrichaceae	Candidatus_Stoquefichus
0.0004	0.0003	1.20	0.57	0.69	Bacteria	Firmicutes	Clostridia	Clostridiales	Ruminococcaceae	Angelakisella
0.0007	0.0006	1.26	0.57	0.70	Bacteria	Firmicutes	Clostridia	Clostridiales	Family_XIII	Family_XIII_AD3011_group
0.0009	0.0010	0.89	0.58	0.70	Bacteria	Firmicutes	Clostridia	Clostridiales	Lachnospiraceae	Coprococcus_3
0.0032	0.0033	0.97	0.59	0.71	Bacteria	Firmicutes	Clostridia	Clostridiales	Ruminococcaceae	Flavonifractor
0.0000	0.0000	1.12	0.62	0.73	Bacteria	Firmicutes	Clostridia	Clostridiales	Ruminococcaceae	Acetanaerobacterium
0.0002	0.0001	1.31	0.62	0.73	Bacteria	Actinobacteria	Coriobacteriia	Coriobacteriales	Eggerthellaceae	Slackia
0.0001	0.0001	0.63	0.62	0.73	Bacteria	Actinobacteria	Actinobacteria	Micrococcales	Micrococcaceae	Rothia
0.0000	0.0000	3.20	0.63	0.73	Bacteria	Firmicutes	Erysipelotrichia	Erysipelotrichales	Erysipelotrichaceae	Dielma
0.0041	0.0055	0.75	0.64	0.74	Bacteria	Bacteroidetes	Bacteroidia	Bacteroidales	Prevotellaceae	Prevotella_9
0.0005	0.0002	2.14	0.65	0.75	Bacteria	Firmicutes	Clostridia	Clostridiales	Lachnospiraceae	Marvinbryantia
0.0004	0.0005	0.92	0.66	0.75	Bacteria	Firmicutes	Clostridia	Clostridiales	Ruminococcaceae	Fournierella
0.0004	0.0003	1.30	0.67	0.75	Bacteria	Firmicutes	Clostridia	Clostridiales	Lachnospiraceae	Coprococcus_1
0.0007	0.0007	0.93	0.68	0.77	Bacteria	Firmicutes	Clostridia	Clostridiales	Peptostreptococcaceae	Intestinibacter
0.0001	0.0001	2.34	0.70	0.78	Bacteria	Firmicutes	Clostridia	Clostridiales	Ruminococcaceae	Hydrogenoanaerobacterium
0.0052	0.0044	1.18	0.72	0.80	Bacteria	Bacteroidetes	Bacteroidia	Bacteroidales	Barnesiellaceae	Barnesiella
0.0000	0.0000	1.48	0.73	0.80	Bacteria	Firmicutes	Clostridia	Clostridiales	Lachnospiraceae	Lactonifactor
0.0001	0.0001	1.16	0.74	0.80	Bacteria	Actinobacteria	Coriobacteriia	Coriobacteriales	Eggerthellaceae	Gordonibacter
0.0016	0.0017	0.98	0.74	0.80	Bacteria	Firmicutes	Bacilli	Lactobacillales	Enterococcaceae	Enterococcus
0.0003	0.0002	1.25	0.75	0.80	Bacteria	Actinobacteria	Coriobacteriia	Coriobacteriales	Eggerthellaceae	Adlercreutzia
0.0002	0.0002	0.79	0.75	0.80	Bacteria	Firmicutes	Clostridia	Clostridiales	Lachnospiraceae	UC5-1-2E3
0.0170	0.0159	1.07	0.79	0.84	Bacteria	Firmicutes	Clostridia	Clostridiales	Ruminococcaceae	Subdoligranulum
0.0028	0.0015	1.82	0.80	0.84	Bacteria	Firmicutes	Clostridia	Clostridiales	Clostridiaceae_1	Clostridium_sensu_stricto_1
0.0015	0.0011	1.33	0.88	0.92	Bacteria	Bacteroidetes	Bacteroidia	Bacteroidales	Prevotellaceae	Prevotella_7

0.0202	0.0206	0.98	0.88	0.92	Bacteria	Bacteroidetes	Bacteroidia	Bacteroidales	Rikenellaceae	Alistipes
0.0006	0.0005	1.05	0.91	0.95	Bacteria	Firmicutes	Clostridia	Clostridiales	Ruminococcaceae	Ruminococcaceae_UCG-010
0.0000	0.0000	1.34	0.95	0.98	Bacteria	Firmicutes	Clostridia	Clostridiales	Ruminococcaceae	Caproiciproducens
0.0002	0.0002	1.32	0.95	0.98	Bacteria	Firmicutes	Bacilli	Lactobacillales	Leuconostocaceae	Weissella
0.0014	0.0009	1.51	0.96	0.98	Bacteria	Firmicutes	Clostridia	Clostridiales	Lachnospiraceae	Tyzzerella
0.0000	0.0000	1.23	0.98	0.99	Bacteria	Firmicutes	Clostridia	Clostridiales	Ruminococcaceae	Harryflintia
0.0053	0.0049	1.09	0.98	0.99	Bacteria	Bacteroidetes	Bacteroidia	Bacteroidales	Marinifilaceae	Odoribacter
0.0012	0.0015	0.80	0.99	0.99	Bacteria	Bacteroidetes	Bacteroidia	Bacteroidales	Prevotellaceae	Paraprevotella

Supplementary Table 6. PubMed search results for *Porphyromonas, Prevotella*, or *Corynebacterium_1*

Species that comprised each genus (and accounted for at least 80% of the ASVs in the genus) were identified based on 100% sequence identity using DADA2-SILVA reference database or 100% or >99% identity and high statistical confidence using NCBI 16S rRNA database. Then each species was searched in PubMed using "genus species" as search term. Search filters: Humans, English, Title/abstract. The citations were tabulated for articles that addressed function, characteristics or relevance to human health; method papers were omitted. All infections are in human samples, except *C. lactis* (newly discovered) was found in an abscess in a companion dog.

Search term	PubMed Return	Subject matter
Corynebacterium amycolatum	https://www.ncbi.nlm.nih.gov/pubmed/10342655	septic arthritis
Corynebacterium amycolatum	https://www.ncbi.nlm.nih.gov/pubmed/10482033	surgical or catheter related infection, pilonidal cyst
Corynebacterium amycolatum	https://www.ncbi.nlm.nih.gov/pubmed/11749760	endocarditis
Corynebacterium amycolatum	https://www.ncbi.nlm.nih.gov/pubmed/12235925	blood cultures
Corynebacterium amycolatum	https://www.ncbi.nlm.nih.gov/pubmed/12439810	mastitis
Corynebacterium amycolatum	https://www.ncbi.nlm.nih.gov/pubmed/12565065	infective endocarditis
Corynebacterium amycolatum	https://www.ncbi.nlm.nih.gov/pubmed/15315020	opportunistic infections
Corynebacterium amycolatum	https://www.ncbi.nlm.nih.gov/pubmed/15786829	Peritonitis
Corynebacterium amycolatum	https://www.ncbi.nlm.nih.gov/pubmed/17284316	endocarditis
Corynebacterium amycolatum	https://www.ncbi.nlm.nih.gov/pubmed/18174873	infections in pediatric oncology
Corynebacterium amycolatum	https://www.ncbi.nlm.nih.gov/pubmed/18809563	endocarditis
Corynebacterium amycolatum	https://www.ncbi.nlm.nih.gov/pubmed/19153032	response to antibiotic tigecycline
Corynebacterium amycolatum	https://www.ncbi.nlm.nih.gov/pubmed/19876565	predominant species in infections in cancer patients
Corynebacterium amycolatum	https://www.ncbi.nlm.nih.gov/pubmed/20624090	resistance to antibiotic macrolide
Corynebacterium amycolatum	https://www.ncbi.nlm.nih.gov/pubmed/22361761	clinical diphtheroid samples
Corynebacterium amycolatum	https://www.ncbi.nlm.nih.gov/pubmed/23806703	surgical site infection
Corynebacterium amycolatum	https://www.ncbi.nlm.nih.gov/pubmed/26324578	vaginosis
Corynebacterium amycolatum	https://www.ncbi.nlm.nih.gov/pubmed/28011352	blood stream infection
Corynebacterium amycolatum	https://www.ncbi.nlm.nih.gov/pubmed/28264610	breast abscess
Corynebacterium amycolatum	https://www.ncbi.nlm.nih.gov/pubmed/28700261	infection of orbital implant
Corynebacterium amycolatum	https://www.ncbi.nlm.nih.gov/pubmed/29793964	respiratory infection after lung transplant
Corynebacterium amycolatum	https://www.ncbi.nlm.nih.gov/pubmed/30102894	Bloodstream and venous catheter-related infections
Corynebacterium amycolatum	https://www.ncbi.nlm.nih.gov/pubmed/30248572	cystic neutrophilic granulomatous mastitis
Corynebacterium amycolatum	https://www.ncbi.nlm.nih.gov/pubmed/30803027	bacteremia
Corynebacterium amycolatum	https://www.ncbi.nlm.nih.gov/pubmed/8727888	clinical isolates, multiple sources
Corynebacterium amycolatum	https://www.ncbi.nlm.nih.gov/pubmed/8874085	sepsis
Corynebacterium amycolatum	https://www.ncbi.nlm.nih.gov/pubmed/9157120	neonatal sepsis fatal in premature infant
Corynebacterium amycolatum	https://www.ncbi.nlm.nih.gov/pubmed/9488824	wound, bloodstream, and urinary tract infections

Corynebacterium amycolatum	https://www.ncbi.nlm.nih.gov/pubmed/9505178	infection after orthopedic surgery
Corynebacterium amycolatum	https://www.ncbi.nlm.nih.gov/pubmed/9868692	Cardioverter-Lead Electrode Infection
Corynebacterium lactis	https://www.ncbi.nlm.nih.gov/pubmed/25937144	Infection in companion dog
Porphyromonas asaccharolytica	https://www.ncbi.nlm.nih.gov/pmc/articles/PMC5896039/	colorectal cancer
Porphyromonas asaccharolytica	https://www.ncbi.nlm.nih.gov/pmc/articles/PMC6247719/	causes Lemierre's syndrome
Porphyromonas asaccharolytica	https://www.ncbi.nlm.nih.gov/pubmed/15528728	clinical isolates
Porphyromonas asaccharolytica	https://www.ncbi.nlm.nih.gov/pubmed/15722627	Clinical isolates, multiple sources
Porphyromonas asaccharolytica	https://www.ncbi.nlm.nih.gov/pubmed/15888469	predominant in polymicrobial flora in 48 inflamed sinuses
Porphyromonas asaccharolytica	https://www.ncbi.nlm.nih.gov/pubmed/15897651	Lemierre's syndrome
Porphyromonas asaccharolytica	https://www.ncbi.nlm.nih.gov/pubmed/16887693	liver abscess
Porphyromonas asaccharolytica	https://www.ncbi.nlm.nih.gov/pubmed/19390440	causes Lemierre's syndrome
Porphyromonas asaccharolytica	https://www.ncbi.nlm.nih.gov/pubmed/21407153	tubo-ovarian abscess
Porphyromonas asaccharolytica	https://www.ncbi.nlm.nih.gov/pubmed/23435719	causes Lemierre's syndrome (acute otopjaryngeal infection)
Porphyromonas asaccharolytica	https://www.ncbi.nlm.nih.gov/pubmed/23474186	pleural empyema in immunocompetent diabetic patient
Porphyromonas asaccharolytica	https://www.ncbi.nlm.nih.gov/pubmed/24679105	polymicrobial foot infection
Porphyromonas asaccharolytica	https://www.ncbi.nlm.nih.gov/pubmed/7548548	extraoral infections
Porphyromonas asaccharolytica	https://www.ncbi.nlm.nih.gov/pubmed/7752213	418 children with infection, found in infections across body sites
Porphyromonas asaccharolytica	https://www.ncbi.nlm.nih.gov/pubmed/7857230	cause chest wall abscess in one woman
Porphyromonas asaccharolytica	https://www.ncbi.nlm.nih.gov/pubmed/8126176	bacterial vaginosis
Porphyromonas asaccharolytica	https://www.ncbi.nlm.nih.gov/pubmed/8518760	male and female genital ulcers
Porphyromonas asaccharolytica	https://www.ncbi.nlm.nih.gov/pubmed/8907604	female genital tract infection
Porphyromonas asaccharolytica	https://www.ncbi.nlm.nih.gov/pubmed/9200028	intravenous catheter related bacteremia in child with cancer
Porphyromonas asaccharolytica	https://www.ncbi.nlm.nih.gov/pubmed/9772922	infected cardiac myxoma
Porphyromonas bennonis	https://www.ncbi.nlm.nih.gov/pubmed/19542133	identification and characterization in clinical specimen from various body sites
Porphyromonas somerae	https://www.ncbi.nlm.nih.gov/pubmed/16145091	chronic skin, soft tissue and bone infections
Porphyromonas somerae	https://www.ncbi.nlm.nih.gov/pubmed/30541687	abscesses, biopsies, wounds
Porphyromonas uenonis	https://www.ncbi.nlm.nih.gov/pubmed/15528728	identification as pathogen
Prevotella bivia	https://www.ncbi.nlm.nih.gov/pubmed/10823756	enhanced HIV expression
Prevotella bivia	https://www.ncbi.nlm.nih.gov/pubmed/10875323	septic arthritis
Prevotella bivia	https://www.ncbi.nlm.nih.gov/pubmed/11368254	bacterial vaginosis
Prevotella bivia	https://www.ncbi.nlm.nih.gov/pubmed/11707013	septic arthritis
Prevotella bivia	https://www.ncbi.nlm.nih.gov/pubmed/14532256	Paronychia
Prevotella bivia	https://www.ncbi.nlm.nih.gov/pubmed/15722627	Clinical specimens
Prevotella bivia	https://www.ncbi.nlm.nih.gov/pubmed/16192439	abdominal cutaneous ulcer
Prevotella bivia	https://www.ncbi.nlm.nih.gov/pubmed/16316686	Lemierre's syndrome
Prevotella bivia	https://www.ncbi.nlm.nih.gov/pubmed/17367470	virulence
Prevotella bivia	https://www.ncbi.nlm.nih.gov/pubmed/1747864	bacterial vaginosis
Prevotella bivia	https://www.ncbi.nlm.nih.gov/pubmed/17982605	penile abscess

Prevotella bivia	https://www.ncbi.nlm.nih.gov/pubmed/18237241	Chorionic plate inflammation
Prevotella bivia	https://www.ncbi.nlm.nih.gov/pubmed/19053926	Oral lichen planus
Prevotella bivia	https://www.ncbi.nlm.nih.gov/pubmed/19271076	septic arthritis
Prevotella bivia	https://www.ncbi.nlm.nih.gov/pubmed/19283879	chest wall abscess
Prevotella bivia	https://www.ncbi.nlm.nih.gov/pubmed/20711427	bacterial vaginosis in HIV infected women
Prevotella bivia	https://www.ncbi.nlm.nih.gov/pubmed/21214658	amniotic fluid infection
Prevotella bivia	https://www.ncbi.nlm.nih.gov/pubmed/21376823	Skin and soft tissue infection
Prevotella bivia	https://www.ncbi.nlm.nih.gov/pubmed/22375046	inguinal bubo
Prevotella bivia	https://www.ncbi.nlm.nih.gov/pubmed/23001520	Abdominal wall phlebitis following renal transplant
Prevotella bivia	https://www.ncbi.nlm.nih.gov/pubmed/24452170	empyema
Prevotella bivia	https://www.ncbi.nlm.nih.gov/pubmed/24787738	Pelvic inflammatory disease
Prevotella bivia	https://www.ncbi.nlm.nih.gov/pubmed/25114266	Necrotizing fasciitis
Prevotella bivia	https://www.ncbi.nlm.nih.gov/pubmed/28008411	Proctitis
Prevotella bivia	https://www.ncbi.nlm.nih.gov/pubmed/28903767	bacterial vaginosis
Prevotella bivia	https://www.ncbi.nlm.nih.gov/pubmed/28931859	bacterial vaginosis
Prevotella bivia	https://www.ncbi.nlm.nih.gov/pubmed/29772525	bacterial vaginosis
Prevotella bivia	https://www.ncbi.nlm.nih.gov/pubmed/29860038	multi-center survey of multi-drug resistant isolates
Prevotella bivia	https://www.ncbi.nlm.nih.gov/pubmed/8013486	endocarditis
Prevotella bivia	https://www.ncbi.nlm.nih.gov/pubmed/8205934	obstetrics gynecology specimen
Prevotella bivia	https://www.ncbi.nlm.nih.gov/pubmed/8270797	association with cervical cancer
Prevotella bivia	https://www.ncbi.nlm.nih.gov/pubmed/8324131	bacterial vaginosis in pregnant women
Prevotella bivia	https://www.ncbi.nlm.nih.gov/pubmed/8677085	bacteremia after C-section
Prevotella bivia	https://www.ncbi.nlm.nih.gov/pubmed/8907604	female genital tract infection
Prevotella bivia	https://www.ncbi.nlm.nih.gov/pubmed/9003606	dog/cat bite wound
Prevotella bivia	https://www.ncbi.nlm.nih.gov/pubmed/9745330	Periodontal abscesses
Prevotella buccalis	https://www.ncbi.nlm.nih.gov/pubmed/14662931	urinary tract infection after renal transplant
Prevotella buccalis	https://www.ncbi.nlm.nih.gov/pubmed/24565649	Endodontic infections
Prevotella buccalis	https://www.ncbi.nlm.nih.gov/pubmed/9266340	Periodontitis
Prevotella disiens	https://www.ncbi.nlm.nih.gov/pubmed/15508748	Periodontitis
Prevotella disiens	https://www.ncbi.nlm.nih.gov/pubmed/1747864	bacterial vaginosis
Prevotella disiens	https://www.ncbi.nlm.nih.gov/pubmed/19161595	bacterial vaginosis
Prevotella disiens	https://www.ncbi.nlm.nih.gov/pubmed/24565649	Endodontic infections
Prevotella disiens	https://www.ncbi.nlm.nih.gov/pubmed/26183701	cranioplasty infection
Prevotella disiens	https://www.ncbi.nlm.nih.gov/pubmed/8205934	obstetrics gynecology specimen
Prevotella disiens	https://www.ncbi.nlm.nih.gov/pubmed/8324131	bacterial vaginosis in pregnant women
Prevotella disiens	https://www.ncbi.nlm.nih.gov/pubmed/8907604	female genital tract infection
Prevotella timonensis	https://www.ncbi.nlm.nih.gov/pubmed/17392225	breast abscess
Prevotella timonensis	https://www.ncbi.nlm.nih.gov/pubmed/29307650	various sites mostly genital and wound

Supplementary Figure 1. Correlation Network Analysis.

Dataset 2 Cases

a

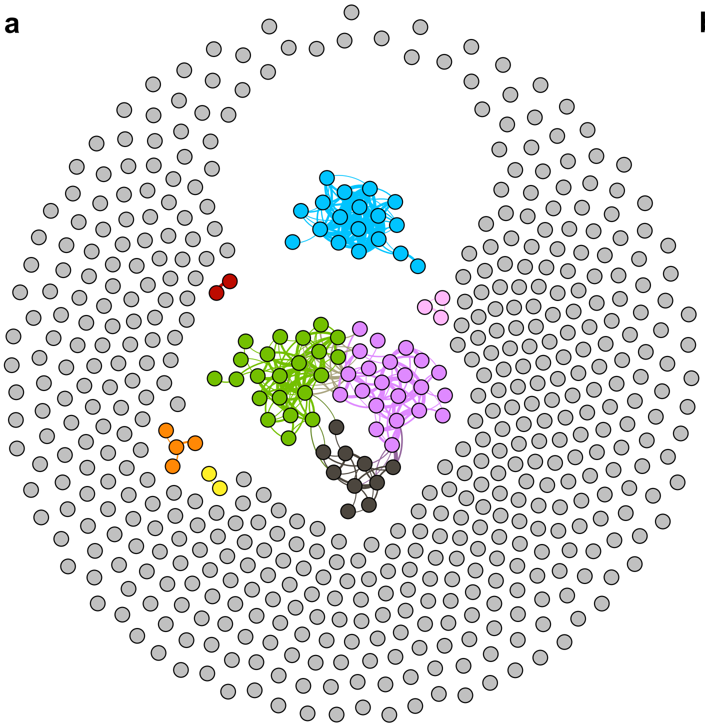

b

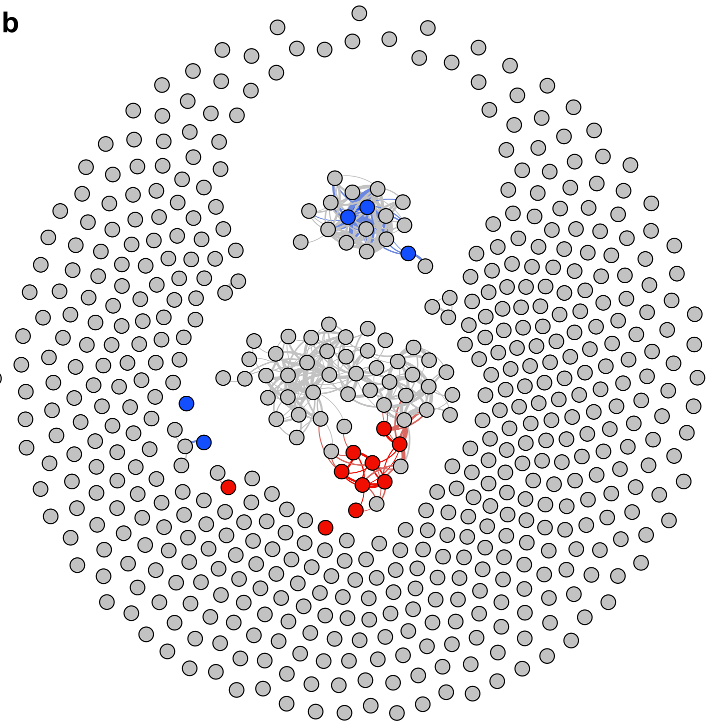

Dataset 2 Controls

a

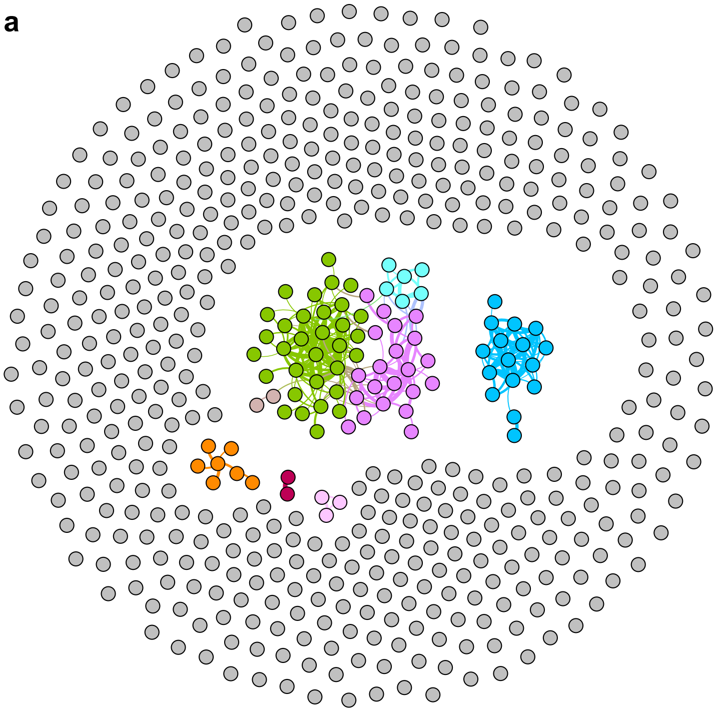

b

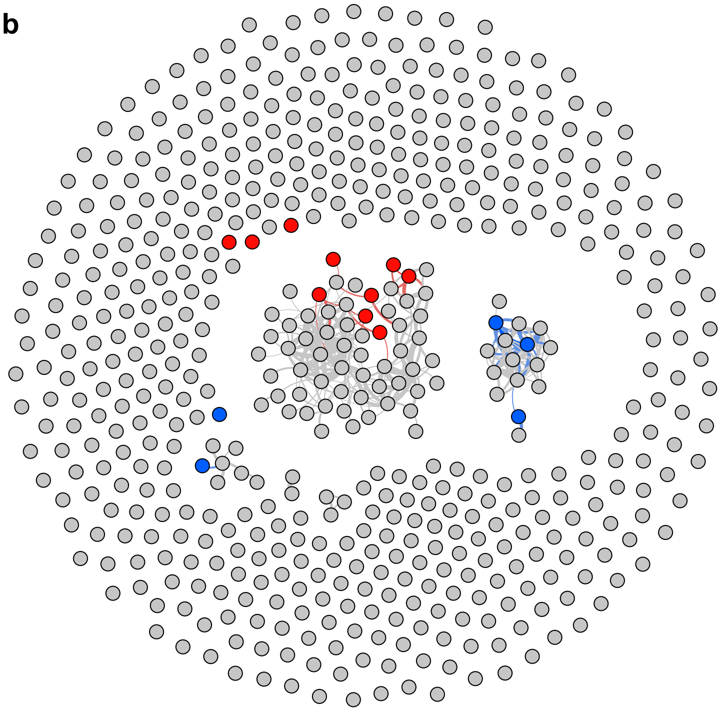

Dataset 1 Cases

a **b**

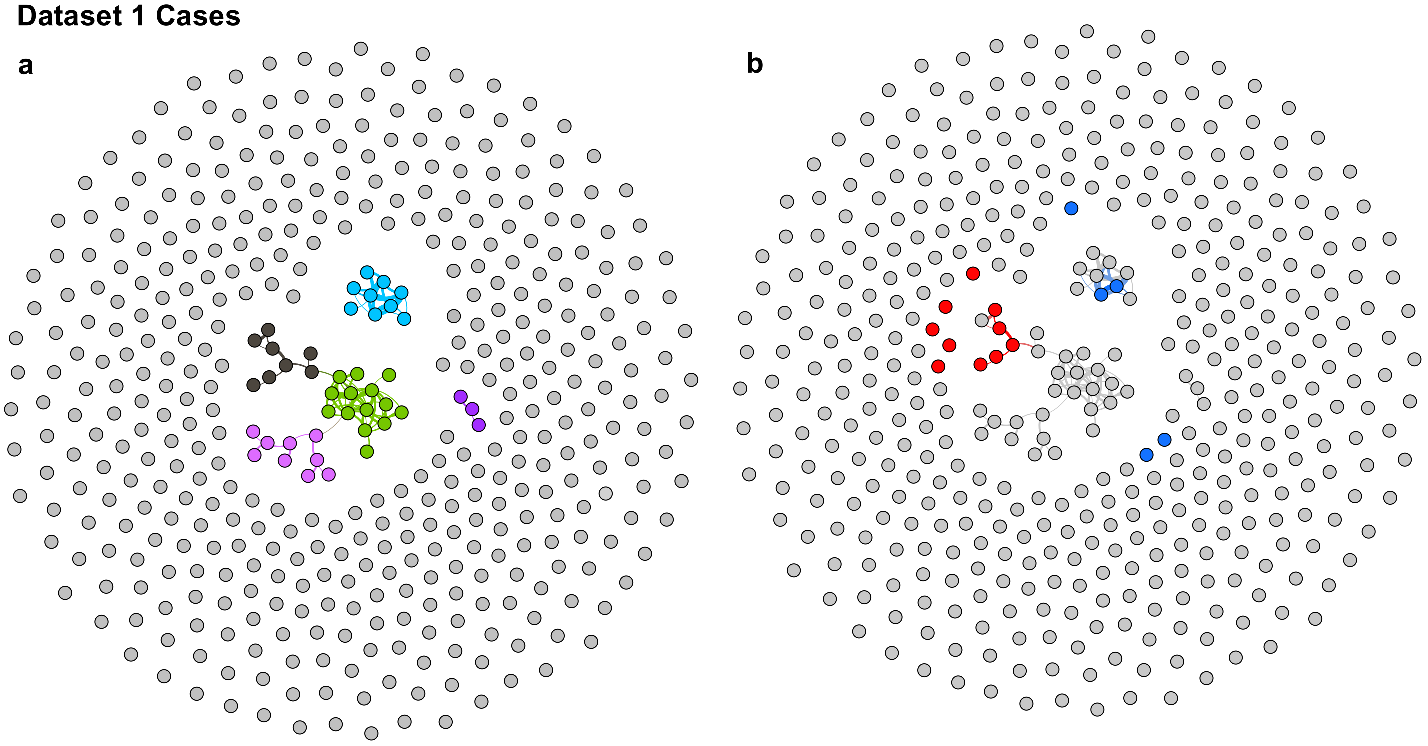

Dataset 1 Controls

a **b**

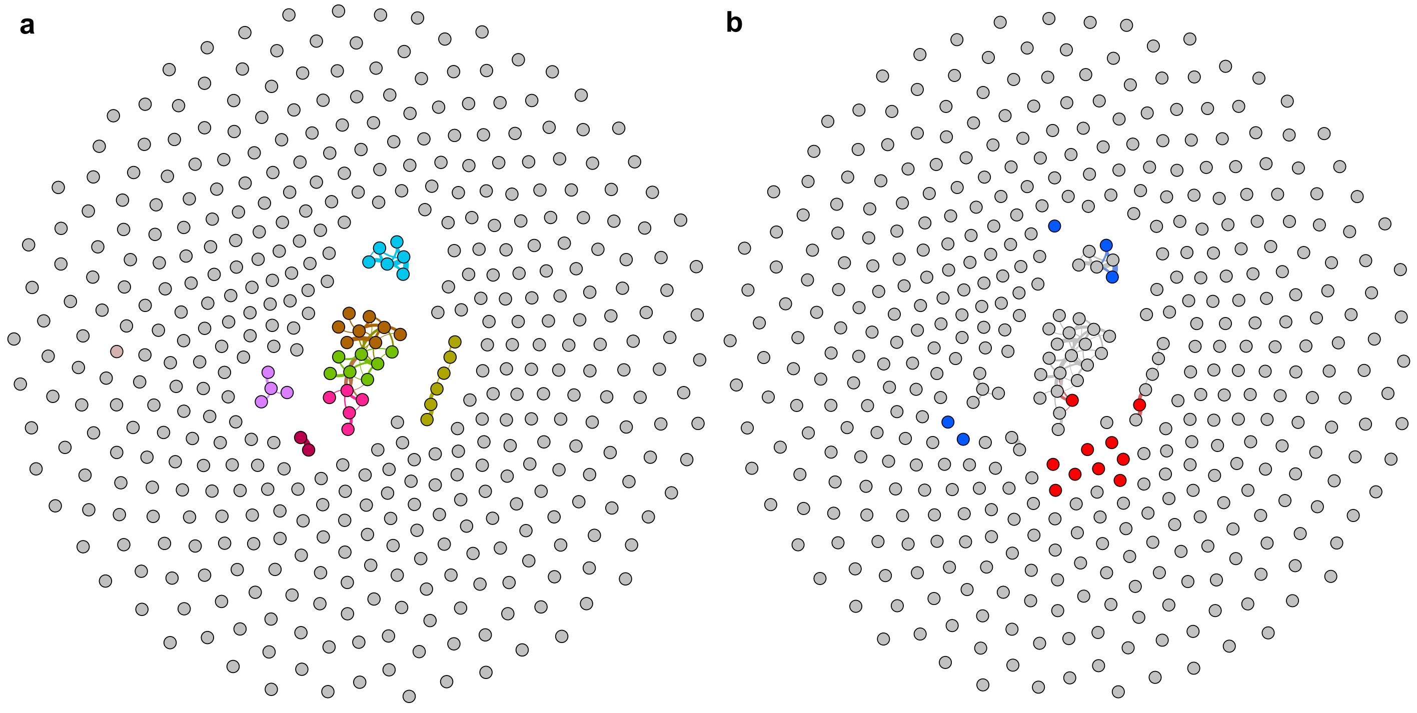

We calculated pairwise correlations in relative abundances for all genera microbiome-wide, for each dataset and in cases and controls separately. Sample size: dataset 1 cases= 201, dataset 1 controls=132, dataset 2 cases= 323, dataset controls =184. To display, we set an arbitrary threshold of correlation coefficient at r≥ |0.4| to connect genera that were correlated. At r≥ |0.4| all correlations were significant at P<3E-4 (the limit for 3,000

permutations). The graphics denoted by “a” display the algorithm-predicted clusters in different colors. Graphics denoted by “b” are identical to their “a” counterpart except algorithm generated colors are now shown in grey and PD-associated taxa are highlighted in blue (if increased in PD) or red (if decreased in PD). Dataset 2 has more power due to larger sample size, and has greater resolution due to deeper sequencing, nonetheless, the general patterns are similar in the two datasets. Generally, the 15 PD-associated genera fall in 3 clusters. As best seen in dataset 2 cases, which has the largest sample size and power, *Porphyromonas, Prevotella*, and *Corynebacterium_1* co-occur in cluster 1. Eight of the 10 in cluster 2 also connect at $r \geq |0.4|$, the other two, *Oscillospira* connects to cluster 2 at r=0.25 (P<3E-4) and *Lachnospiraceae_UCG-004* connects at r=0.35 (P<3E-4). *Lactobacillus* and *Bifidobacterium* connect to each other (cluster 3) at r=0.33 (P<3E-4).
